# Timing of intrapartum transfusion among obstetric patients, stratified by admission and discharge haemoglobin, relative to anaesthesia care: A retrospective cohort study

**DOI:** 10.1111/tme.70082

**Published:** 2026-04-26

**Authors:** Franklin Dexter, Richard H. Epstein, Unyime S. Ituk

**Affiliations:** ^1^ Department of Anesthesia University of Iowa Iowa City Iowa USA; ^2^ Department of Anesthesiology University of Miami Miami Florida USA

## Abstract

**Background:**

We previously validated and implemented criteria for auditing anaesthesiologists with intraoperative transfusion based on low median blood loss of the surgical procedure or absence of anaemia before transfusion. However, obstetrical cases were excluded during implementation, as the peripartum blood management protocol does not include haemoglobin determinations, matching American College of Obstetricians and Gynaecologists recommendations. For non‐obstetrical surgery, intraoperative transfusion is associated with an increased probability of postpartum transfusion. Because of the obstetrical transfusion approach, we hypothesized the opposite relationship between intrapartum and postpartum transfusion.

**Methods:**

The retrospective cohort study included all 30 160 patients who delivered at the University of Iowa January 2013 through March 2025.

**Results:**

Any RBC administration during anaesthesia care reduced the odds of later transfusion (odds ratio 0.14, 99% confidence interval (CI) 0.11%–0.18%). Single‐unit RBC administration during anaesthesia reduced such odds (0.09, 99% CI 0.06%–0.11%), as did multiple‐unit transfusion (0.18, 99% CI 0.13%–0.24%). McNemar tests had Bonferroni‐adjusted *p*‐values <0.0001. While antepartum anaemia (haemoglobin <11 g/dL) increased the probability of transfusion by 7.41% (standard error 0.43%) outside of anaesthesia care, the RBC transfusion decision during anaesthesia care was less influenced by anaemia, 1.52% (0.22%), *p* < 0.0001. Many (59%, 99% CI 50%–69%) RBC units started during anaesthesia care were administered to patients who were not anaemic (haemoglobin ≥11 g/dL) at admission. Most patients with single‐unit RBC transfusion and blood loss <2500 mL had stable haemodynamics.

**Conclusions:**

Unlike several categories of non‐obstetrical procedures, RBC transfusion during anaesthesia care for delivery was associated with a reduced probability of postpartum RBC transfusion. A reasonable approach for blood bank auditing of anaesthesiologists' obstetric RBC transfusion decision‐making would be to review cases when RBC were administered, there was blood loss <2500 mL, no subsequent transfusion, and discharge haemoglobin ≥9 g/dL.

## 1 INTRODUCTION

Earlier, we developed, validated, and implemented criteria for auditing charts to adjudicate intraoperative red blood cell (RBC) decision‐making based on the median blood loss of the surgical procedure (low) or the patient's haemoglobin level before transfusion (normal or high).^1^ Although audit development included caesarean delivery cases,^1^ implementation within the department has not been applied to patients receiving obstetrical anaesthesia care. Our institution's obstetric haemorrhage management protocol recommends RBC transfusion based on blood loss and haemodynamic instability but does not incorporate either the admission haemoglobin or repeated measurements during anaesthesia care (Appendix S1). This protocol matched the American College of Obstetricians and Gynaecologists' Practice Bulletin on postpartum haemorrhage that addresses the management of anaemia “after a patient has been stabilized post‐hemorrhage.”^2^

In patients undergoing the non‐obstetrical procedures of acetabular fracture repair,^3^ hip fracture repair,^4^ thoracolumbar spine surgery,^5^ percutaneous nephrolithotomy,^6^ microvascular free tissue transfer,^7^ cardiac surgery,^8^ and hepatic transplantation,^9^ intraoperative transfusion was associated with increased probability of postpartum transfusion. Mechanisms for the association include common mediators of the baseline haemoglobin and large blood loss. If the opposite relationship holds true in obstetric patients, then criteria to select cases with RBC administration during anaesthesia care for audit can validly be based on the discharge haemoglobin (e.g., ≥9 g/dL).

Our study's primary hypothesis was that RBC transfusion during anaesthesia care for delivery was associated with reduced probability of immediate postpartum RBC transfusion. (Note, everywhere when we refer to “postpartum,” we mean the immediate puerperium [postpartum] period.) Our study was novel in considering this timing of RBC transfusion, intrapartum versus postpartum (Appendix S2, our PubMed search).^10^ We hypothesized the primary association would differ between the non‐obstetrical^3^, ^4^, ^5^, ^6^, ^7^, ^8^, ^9^ and obstetrical patients because any of the following three mechanisms would result in the primary association.

- *First*, RBC transfusion decisions following our institutional obstetrical guideline (Appendix S1) would be based on blood loss but not haemoglobin, in contrast to non‐obstetric care.^1^ In patients without pre‐existing anaemia (i.e., haemoglobin ≥11 g/dL), the criteria would lead to some patients having received RBC transfusion for haemoglobin >7 g/dL.
- *Second*, during acute volume resuscitation, some anaesthesiologists may have conducted RBC transfusion without understanding that most RBC units transfused are administered postpartum. In other words, the anaesthesia team may have conceptually targeted a moderate anaemia haemoglobin ≥9 g/dL, not ≥7 g/dL.^11^, ^12^
- *Third*, transfusion practices during anaesthesia care may have targeted the prevention of antepartum anaemia (e.g., <11 g/dL) as compared with a suitable postpartum transfusion trigger such as moderate anaemia (e.g., <9 g/dL)^13^ or severe anaemia (e.g., <8 g/dL).^14^

If one or more of these three mechanisms applied, then the primary association would be expected. The obstetricians' protocol postpartum was transfusion for haemoglobins <7 g/dL, and 7–8 g/dL when patients were symptomatic.^15^

We tested our primary hypothesis in part because of the potential association of reduced transfusion with antepartum intravenous iron infusion. Like at other facilities, worldwide,^16^ our department established our hospital's anaemia management clinic,^17^, ^18^ which is used often for antenatal patients. Suppose that among patients with blood loss <2500 mL the RBC transfusion decisions by anaesthesiologists were largely made without consideration of the pre‐transfusion haemoglobin, matching its omission from the departmental guideline (Appendix S1). Then, the benefit gained from the anaesthesia department's role in antepartum treatment of anaemia would be counterbalanced by the lack of reliance on that haemoglobin when making initial RBC transfusion decisions.

## 2 METHODS

The University of Iowa approved this retrospective cohort study # 201911151 as exempt from written informed consent. The cohort included every patient who delivered at the University of Iowa university hospital January 2013 through March 2025 (Table 1).^14^, ^19^, ^20^, ^21^, ^22^ This is the only level III maternal care teaching hospital in the state (e.g., caring for approximately 70% of the patients with placenta accreta statewide).^23^ Our cohort included all caesarean and vaginal deliveries at our hospital, irrespective of the participation of an anaesthesiologist (Table 1). The study start date was chosen as the first full year in which both the obstetric and anaesthesia modules of the Epic electronic health record had been implemented at the University of Iowa (Epic Systems, Veronia, Wisconsin). The last date was just before we performed the analysis. For convenience, we refer to each delivery as representing a “patient,” but the analyses were by delivery.

**TABLE 1:** Characteristics of studied deliveries, vaginal or caesarean delivery, with or without anaesthesiologist, all patients, with subsets of patients at their haemoglobin in Appendices S3 and S4.

Variable	% (*n*/*N*) or *N*	Median [25th, 75th percentiles]	Mean (standard deviation)
Caesarean birth	35% (10 667/30160)		
Anaesthesiologist (billed) care during hospitalisation	89% (26 703/30160)		
Anaesthesiologist care at time of delivery	69% (20 887/30160)		
Maternal age, years	30 160	30 [26, 33]	29.7 (5.4)
Parity	30 084	2 [1, 3]	2.1 (1.3)
Admitted hours before delivery^a^	30 160	11.0 [4.7, 21.0]	28.5 (94.2)
Discharge hours after delivery^a^	30 160	50.2 [40.4, 65.0]	53.8 (31.8)
Any RBC transfused^b^	4.4% (1329/30160)		
Any RBC transfused during anaesthesia care^c^	0.9% (285/30160)		
≥2 units RBC transfused^b^	2.0% (605/30160)		
≥2 units RBC transfused during anaesthesia care	0.4% (110/30160)		
RBC units transfused if any	1329	1 [1,2]	1.95 (1.91)
RBC units transfused, if any, during anaesthesia care	285	1 [1,2]	1.62 (1.27)
Quantitative blood loss in mL	18 791	443 [242, 788]	599 (538)
Blood loss, quantitative and otherwise estimated	28 676	400 [250, 798]	574 (539)

*Note*: RBC represents packed red blood cells.

^a^ If >1 births during a delivery (e.g., twins), the time used was the birth of the first baby.

^b^ Among the patients with RBC transfusion, 54% (724/1329) were single‐unit RBC transfusions. In comparison, in 2018, after a period of clinician education on best blood transfusion practice, a maternity hospital in Ireland had 55% (58/105) single‐unit transfusions.^20^

^c^ The percentage of patients with at least one RBC transfusion during care of an anaesthesiologist was 21% (285/1329). Similarly, at a maternity hospital in Singapore, 23% (197/863) of deliveries with transfusion were in an “operating room,” suggesting anaesthesiologist's presence.^19^

Data for each patient comprised the timing of red blood cell transfusions, Healthcare Procedure Coding System codes (e.g., to find caesarean births), intravenous iron infusions, anaesthesia care, and haemoglobin laboratory values. The study intervals for each patient were from 9 months before the time of delivery through postpartum discharge from the hospital. We used 9 months to include all antepartum intravenous iron infusions and haemoglobin laboratory values. The cohort was divided into three overlapping groups: (i) all patients (Table 1), (ii) patients with haemoglobin measured before delivery (Appendix S3), and (iii) patients with haemoglobin measured before and after delivery (Appendix S4). All patients in Appendix S4 were included in Tables 1 and S3, but many patients in Table 1 were not in Appendix S4. The “allowable blood loss” while maintaining an estimated Hgb ≥7 g/dL was calculated from the last haemoglobin before delivery and patient weight using the formula applied by Russo et al.^12^ (Appendices S3 and S4).

Statistical coding and analyses were performed using Stata v19.5 (StataCorp, College Station, Texas). The Stata code and output log are included as Appendix S7. To test the primary hypothesis (i.e., whether RBC transfusion during anaesthesia care was associated with a reduced probability of postpartum transfusion), pairwise associations by delivery were made between RBC units transfused during anaesthesia care and not during anaesthesia care using McNemar's test with exact *p*‐value. Pairwise analyses were performed by patient because thereby there was statistical control for all potential confounding factors. These confounders included patient demographic characteristics including preexisting diseases, caesarean delivery, or antepartum treatment of anaemia. The same Stata *mcc* command gave the confidence intervals for the paired odds ratios. We would consider the sample size to have been sufficient, in retrospect, if the 99% two‐sided confidence interval fully ruled in or out an odds ratio of 1.25 or 0.8, where 0.8 = 1/1.25. Throughout the article we were conservative in treating *p* < 0.01 as statistically significant, rather than *p* < 0.05, because we have multiple tests, requiring adjustment. Specifically, the primary calculations were performed three times: overall, repeated for single unit RBC transfusions, and repeated for multiple unit transfusions. The 0.05/3 = 0.017, which we rounded down to 0.01 to mitigate against presenting a false result.

Secondary questions were used to assess (i) predictive validity of the cohort, (ii) generalizability of the results, (iii) potential managerial recommendations, and (iv) the associations of RBC transfusion with antepartum anaemia treatment. For (i), the predictive effect of anaemia at admission on RBC units transfused was evaluated using the Wilcoxon–Mann–Whitney test with exact P‐value. To assess (ii), the association between anaemia and the probability of RBC transfusion was compared between anaesthesia and non‐anaesthesia group's RBC administration using marginal comparisons after generalised linear modelling with binomial family, logit link, and robust variance estimation, with clustering by patient. This multivariable modelling was repeated replacing the binary variable of anaemia with the allowable blood loss centered at its mean, calculating the ratio of the marginal effects (i.e., slopes) using the delta method. To address (iii), the predictive effects of RBC units transfused during anaesthesia care, blood loss, and admission haemoglobin on discharge haemoglobin ≥9 g/dL were evaluated using Wilcoxon–Mann–Whitney tests. For (iv), confidence intervals for the ratios of mean units RBC transfused during anaesthesia care versus at any antepartum period were calculated using first‐order Taylor expansion (i.e., delta method; Stata *ratio* command). The confidence intervals for proportions were Clopper–Pearson intervals, which were selected because these intervals are deliberately conservative. Fisher exact tests were similarly used to compare unpaired proportions. Among these secondary analyses, there are three paragraphs with five *p*‐values whose results evaluate the three mechanisms described in the introduction. The multiple comparison adjustment, treating *p* < 0.01 as significant (i.e., 0.05/5), is suitable for these tests related to our primary analysis.

## 3 RESULTS

Table 1 shows the summary data for all *N* = 30 160 patients, 35% caesarean births. Appendix S3 shows the data for the *N* = 27 114 patients with a haemoglobin checked at the time of or soon before admission for delivery, without a follow‐up value during the hospitalisation. Appendix S4 shows the corresponding data for the *N* = 4817 patients with haemoglobin measured both on admission and with at least one before hospital discharge. The corresponding percentages of patients in these three groups receiving one or more RBC units were 4.4%, 4.7%, and 20.9%, respectively. Before delivery, 19% and 24% of the patients were anaemic (haemoglobin <11 g/dL) (Appendices S3 and S4). After delivery, 18% of the patients had moderate or severe anaemia (haemoglobin <9 mg/dL)^13^ and 6% had severe anaemia (haemoglobin <8 g/dL)^14^ (Appendix S4).

Three comparisons were made pairwise by delivery to address our primary hypothesis that patients who received RBC transfusion while under anaesthesia care had a lower incidence of transfusion during other periods compared to patients not receiving transfusion during anaesthesia care. First, any RBC administration during anaesthesia care *reduced* the odds of later transfusion (paired odds ratio 0.14, 99% CI 0.11%–0.18%). Second, single‐unit RBC administration during anaesthesia care reduced the odds of later transfusion of any RBC (0.09, 99% CI 0.06%–0.11%). Third, RBC transfusion during anaesthesia care of more than one unit reduced the odds of later transfusion of multiple RBC units (0.18, 99% CI 0.13%–0.24%). The three McNemar tests all had Bonferroni adjusted exact *p* < 0.0001. The three effect sizes were large compared with the ratios that we considered the smallest important.

### 3.1 Secondary: anaesthesiologists followed the departmental transfusion management guideline (Appendix S1)

Figure 1 shows graphically how the primary association between haemoglobin and probability of RBC transfusion depended on the group: transfusion during anaesthesia care or transfusion outside of anaesthesia care. Haemoglobin <11 g/dL (i.e., anaemia at admission) was associated with a greater probability of RBC transfusion and a greater number of RBC units transfused (both *p* < 0.0001).^24^, ^25^ For transfusion not during anaesthesia care, anaemia (<11 g/dL) increased the probability of transfusion by 7.41% (standard error 0.43%). In contrast, during anaesthesia care, the RBC transfusion decision (vertical axis) was less influenced by anaemia (i.e., horizontal axis); 1.52% (standard error 0.22%). The marginal difference between the two groups in the effect of anaemia on the transfusion decision was 5.89% (standard error 0.49%), *p* < 0.0001. These results are consistent with the first of the mechanisms described in the introduction, specifically that RBC transfusion decisions followed our institutional obstetrical guideline (i.e., the admission haemoglobin was rarely included in the transfusion decision‐making) (Appendix S1).

**FIGURE 1:**
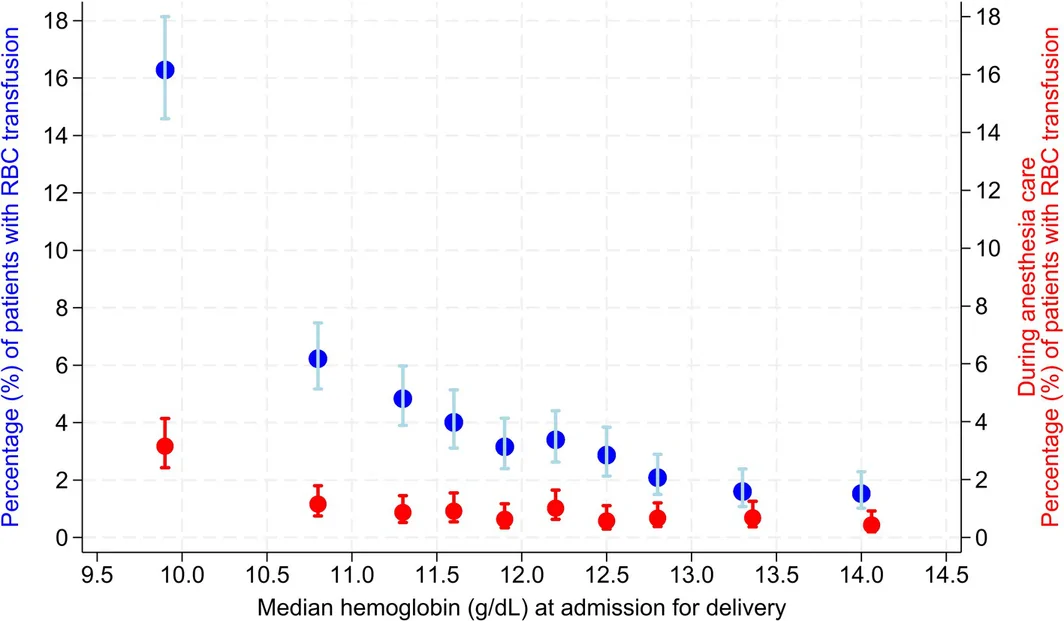
Association of haemoglobin at admission and proportion of deliveries with RBC transfusion. RBC stands for red blood cells. The estimated percentages are shown along with Wilson‐method 99% two‐sided confidence intervals, one per decile of haemoglobin. The median haemoglobin is plotted for each decile. For the two upper deciles, the red dots' median haemoglobin were jittered by +0.06 so that there would not be overlapping with the left‐axis blue all‐patient data. There were 19% of the patients with haemoglobin <11 g/dL (5203/27114). By Wilcoxon–Mann–Whitney test, these patients with antepartum anaemia had significantly more RBC units transfused (*p* < 0.0001 after Bonferroni adjustment for three comparisons), mean 0.247 (standard deviation 0.987) units versus 0.055 (0.436) units. They also had more RBC units transfused during anaesthesia care, including labour analgesia (*p* < 0.0001 after Bonferroni adjustment), with a mean of 0.036 (0.286) units versus 0.012 (0.185) units. Finally, they had more RBC units transfused not during anaesthesia care (*p* < 0.0001). The graphical relationships are the same when plotted for two or more RBC units transfused. This figure illustrates predictive validity, as an earlier study with a different cohort of patients undergoing caesarean delivery showed that, among 20 antepartum predictors of intraoperative RBC transfusion, haemoglobin had the greatest observed feature importance based on the Shapley additive explanation values.^24^ In a third cohort, haemoglobin <11 g/dL was associated with increased (2.34) odds of transfusion, with a reported lower 95% confidence limit of 1.4.^25^

Because the admission haemoglobin and allowable blood loss were highly correlated (Spearman rho = 0.99), the findings in the preceding two paragraphs can be interpreted in terms of blood volumes. The 2500 mL criteria in haemorrhage protocol (Appendix S1) matched the calculated “allowable blood loss” to keep the haemoglobin ≥7 g/dL for half the patients (Appendix S3). During anaesthesia care, each 1 L decrease in the allowable blood loss was associated with a 1.55% absolute increase in the probability of transfusion (standard error 0.15%), versus 7.38% (0.28%) without anaesthesiologists' care. Consequently, the anaesthesiologists were 4.8‐fold less likely to transfuse for each 1 L decrease in the allowable blood loss. Thus, their behaviour had *increased* the risk of haemoglobin <7 g/dL (99% CI 3.5%–6.0%, *p* < 0.0001). For example, if the calculated estimated allowable blood loss was 1000 mL, due to antepartum anaemia, using the higher blood loss threshold in the departmental guideline would have resulted in an insufficient mass of red cells to maintain ≥7 g/dL.

Among the patients having their haemoglobin checked before delivery (Appendix S3), 59% of RBC units started during anaesthesia care (268/453) were administered to patients who were not anaemic (haemoglobin ≥11 g/dL) at admission (99% CI 50%–69%). Similarly, 60% (155/258) of the patients with the first unit started during anaesthesia care were initially not anaemic (i.e., antepartum haemoglobin ≥11 g/dL). Thus, whether analysed by transfused units or by patients, transfusion practice was consistent with the anaesthesiologists following their departmental guideline (Appendix S1), without incorporation of the admission haemoglobin.

### 3.2 Secondary: departmental guideline mitigated the benefit of intravenous iron to prevent transfusion

Appendix S5 shows that there was a greater probability of RBC transfusion if the first antenatal haemoglobin was <11 g/dL (*p* < 0.0001). Appendix S6 shows that 6% (1811/30160) of obstetrical patients were treated with intravenous iron, achieving increases in their haemoglobin level by admission (*p* < 0.0001),^26^ even if the first intravenous iron treatment(s) was administered 4–6 weeks before delivery.^26^ Combining these results with those of the preceding paragraph (i.e., most RBC units transfused during anaesthesia care to patients with admission haemoglobin ≥11 g/dL), these data demonstrate that the anaesthesiologists' RBC transfusion decisions significantly offset the haemoglobin increases achieved by intravenous iron treatment.

### 3.3 Secondary: obstetricians' decision‐making appears to have been based on the haemoglobin

Among all patients in the cohort (Table 1), most RBC units were administered more than 4 h after delivery (i.e., outside of anaesthesia care, Table 2).^11^ In contrast, among the minority of RBC units administered during anaesthesia care, 90% (418/462) were started between 1 h before and ≤4 h after delivery (Table 2). Much less than half the RBC units were administered during care of an anaesthesiologist, 18% (462/2590; 99% CI 15%–21%, *p* < 0.0001).^19^, ^27^ These distributions align with transfusion decisions during anaesthesia care having been made without considering that most RBC units were administered postpartum by the obstetrical team (i.e., based on postpartum haemoglobins). The result matters because it is the second of the three potential mechanisms described in the introduction to explain our primary association.

**TABLE 2:** Timing of the start of red blood cell transfusion relative to the time of delivery^a^.

Variable	≤ − 1 h	> − 1 to ≤2 h	>2 to ≤4 h	>4 to ≤12 h	>12 to ≤24 h	>24 to ≤48 h	>48 h^b^
% (*n*) of the 1329 patients with RBC transfusion^c^	6% (78)	27% (363)	11% (148)	11% (143)	12% (166)	20% (272)	12% (159)
% (*n*) of the 1065 patients with first unit transfused not started during care of an anaesthesiologist	6% (69)	14% (146)	12% (126)	12% (130)	16% (165)	26% (272)	15% (157)
% (n) of the 264 patients with first unit transfused started during anaesthesia care	3% (9)	82% (217)^d^	8% (22)	5% (13)	0% (1)	0% (0)	1% (2)
% (*n*) of the 2590 RBC units^b^, ^e^	6% (146)	27% (702)	9% (245)	12% (303)^e^	13% (340)^e^	19% (489)^e^	14% (365)^b^, ^e^
% (*n*) of the 2128 RBC units not started during care of an anaesthesiologist	6% (134)	16% (334)	9% (195)	13% (277)	16% (336)	23% (489)	17% (363)
% (*n*) of the 462 RBC units started during anaesthesia care^f^	3% (12)^f^	80% (368)^d^, ^f^	11% (50)^f^ ^s^	6% (26)	1% (4)	0% (0)	0% (2)

*Note*: RBC represents packed red blood cells.

^a^ If >1 delivery (e.g., twins), the time used was the delivery of the first baby.

^b^ Among all births in Sweden, dates of delivery were compared with the calendar dates of RBC transfusion.^11^ As the above shows, 14% of RBC transfusion “>48 h” after delivery, they similarly observed 14% for RBC transfusions three or more calendar days following delivery.^11^

^c^ This table was produced using all data in the cohort, those in the left‐most column of data in Table 1.

^d^ These rows do not simply show that caesarean deliveries typically take approximately 2 h, although true, because many of the patients receiving RBC during anaesthesia care were undergoing management of uterine atony after vaginal delivery.

^e^ Most (58%) of RBC units were administered >4 h after delivery, where 58% = (303 + 340 + 489 + 365)/2590.

^f^ 90% of RBC units administered during anaesthesia care were started 1 h before to 4 h after delivery; 90% = (368 + 50)/462.

Among the patients with RBC units started during anaesthesia care, there was a *greater* incidence of moderate to severe anaemia (haemoglobin <9 g/dL)^13^ at discharge in the patients *without* subsequent RBC transfusion (Table 3; 55.9% vs. 14.6%, *p* < 0.0001).^15^ Thus the obstetrical team's decisions not to transfuse counterbalanced the earlier decisions to transfuse, the opposite of care during non‐obstetrical^3^, ^4^, ^5^, ^6^, ^7^, ^8^, ^9^ procedures, where intraoperative transfusion increased the probability of postpartum transfusion. Exploring further, we limited consideration to the patients receiving at least one RBC unit during anaesthesia care but no subsequent transfusion. There were 85% (111/130) of these patients discharged with haemoglobin ≥9 g/dL. Most had blood loss <2500 mL (Appendix S1). The admission haemoglobin was significantly higher among patients with haemoglobin ≥9 g/dL upon discharge, *p* = 0.0011 (mean 11.5 (1.4) g/dL [*N* = 111] vs. 10.0 (1.9) g/dL [*N* = 19]). This finding highlights the strong statistical association of the admission haemoglobin in predicting RBC transfusion decisions among patients with blood loss <2500 mL. The admission haemoglobin (i.e., and the allowable blood loss) was known for 96% of patients transfused (1279/1329), 98% of those transfused during anaesthesia care (279/285), and 99% of the patients transfused during anaesthesia care and with blood loss <2500 mL (166/168). This finding is consistent with the third mechanism. While our observational design precludes a determination of which specific mechanism(s) drove the primary association, any would be sufficient and our cohort's data were consistent with all three mechanisms.

**TABLE 3:** Contingency table (2 × 2) of the association of red blood cell transfusion with haemoglobin upon discharge and blood loss, each cell reported as: median [25th, 75th percentiles]; mean (standard deviation); % < 9 g/dL (*n*/*N*).

Units transfused during anaesthesia care?	0 units transfused before (rarely) or after (usually) anaesthesia care	≥1 units transfused before (rarely) or after (usually) anaesthesia care		
	Discharge Hgb	Blood loss	Discharge Hgb	Blood loss
0 units transfused during anaesthesia care	10.6 [9.7, 11.5]; 10.6 (1.3); 9.3% (354/3,809)^a^	900 [511, 1284]; 941 (526); *N* = 3,704	8.7 [8.0, 9.7]; 8.9 (1.3)^b^; 55.7% (418/751)^a^	1601 [1160, 2069]; 1738 (1586); *N* = 694
≥1 units transfused during anaesthesia care	10.2 [9.2, 11.1]^c^; 10.2 (1.4); 14.6% (19/130)^a^	2135 [1618, 2500]^c^; 2197 (1128); *N* = 122	8.8 [7.9, 9.7]; 8.8 (1.3)^b^; 55.9% (71/127)^a^	2500 [2021, 3200]; 2736 (1220); *N* = 127

*Note*: Hgb represents haemoglobin in g/dL.

^a^ The sum of the denominators (3809 + 751 + 127 + 130) equals 4817, the number of deliveries listed in the first row of Appendix S4, the table among patients with both admission and pre‐discharge haemoglobin measurements.

^b^ The weighted mean discharge haemoglobin among patients receiving postpartum transfusion after anaesthesia care was 8.9 (1.3) g/dL. In comparison, patients in the randomised clinical trial of symptoms after single‐unit or multiple‐unit transfusion had a mean of 8.2 (1.1) g/dL; see Section 4.^15^

^c^ See Table 4 for hemodynamic data prior to transfusion in patients receiving a single RBC unit during the anaesthesia care. Among the 130 patients in Table 3, there were 30 with discharge haemoglobin ≥9 g/dL, caesarean delivery for continuous monitoring, single unit RBC transfusion, and blood loss <2500 mL, accounting for 1.7% of total transfused RBC units.

### 3.4 Secondary: most patients with blood loss <2500 mL lacked haemodynamic compromise

Among the 20,887 obstetrical patients with anaesthesia care, there were 0.4% (74) with RBC transfusion during anaesthesia care and zero afterwards, blood loss <2500 mL, and discharge haemoglobin ≥9 g/dL. There were one unit transfused for 71% (56), two units for 27% (21), and three or four units for 2% (2). We examined haemodynamic and vasopressor administration in the 30 patients who underwent caesarean delivery and had single units transfused (Table 4). During the 15 min before the start of RBC transfusion, some patients clearly had severe hemodynamic instability, a criterion for transfusion (Appendix S1). For example, among the 2 (7%) patients with median systolic blood pressure <90 mmHg, the lowest systolic blood pressures were 68 and 74 mmHg in 2 patients (7%) (Table 4). There were seven patients (23%) who received >1000 mcg phenylephrine over the 15 min (Table 4). However, most patients had normal haemodynamics and unremarkable pressor doses (Table 4). Among these patients, the second mechanism described above would explain the primary association: transfusion decisions made without accounting for the fact that most RBC units are administered postpartum by the obstetrical team. The third mechanism could also apply, specifically anaesthesia transfusion practices during obstetric care targeting the prevention of antepartum anaemia (e.g., <11 g/dL) as compared with a suitable postpartum transfusion trigger.

**TABLE 4:** Haemodynamics and vasopressors administered during the 15 min before the start of transfusion among patients who underwent caesarean delivery, had total blood loss <2500 mL, received a single red blood cell unit, had both admission and pre‐discharge haemoglobin measurements, and had discharge haemoglobin ≥9 g/dL.

Variable	% (*N*)
Blood loss ≥1500 mL	77% (23/30)
Blood loss ≥allowable blood loss, from Russo et al.^12^	32% (9/28)
Median heart rate >100 per minute	27% (8/30)
Median heart rate >120 per minute	7% (2/30)
Median systolic blood pressure <110 mmHg	33% (10/30)
Median systolic blood pressure <90 mmHg	7% (2/30)
Phenylephrine >500 mcg	30% (9/30)
Phenylephrine >1000 mcg	23% (7/30)
Vasopressin >0 units	3% (1/30)
Vasopressin >1 unit	0/30
Epinephrine, ephedrine, or norepinephrine	0/30

## 4 DISCUSSION

Figure 2 provides a conceptual summary of our study, illustrating potential mechanisms driving the primary association between RBC transfusion during anaesthesia care and postpartum transfusion (left), the primary association including among subpopulations (middle), and the resulting managerial and clinical implications (right). Given that there was an admission haemoglobin (i.e., known allowable blood loss)^12^ for 99% (166/168) of the patients transfused during anaesthesia care and with blood loss <2500 mL, our focus was the implications for choosing which cases to be audited.^1^ The objective of the audit is to determine if the transfusion decision was justifiable or not. Table 1 shows that, with the use of anaemia management clinics offering intravenous iron infusion,^18^, ^26^ such as at the studied hospital's obstetric program,^17^ the vast majority of patients had haemoglobin ≥11 g/dL at admission for delivery, including those receiving RBC transfusion during anaesthesia care. Table 2 shows that most RBC units transfused intrapartum (58% (1497/2590 units)) were administered >4 h after delivery, and Table 3 shows that most of those patients (56% (489/878 patients)) were discharged with haemoglobin <9 g/dL. Consequently, RBC transfusion during anaesthesia care was associated with significantly *lower* odds of postpartum transfusion, even though the blood loss was greater among the patients receiving postpartum transfusion (Table 3). This contrasts with the findings in non‐parturient surgical patients.^3^, ^4^, ^5^, ^6^, ^7^, ^8^, ^9^ The main managerial implication is that audit criteria for cases with RBC transfusion during care of an anaesthesiologist can validly rely on a discharge haemoglobin ≥9 g/dL. Such audit would occur for only ≈0.4% of obstetric patients with anaesthesia care (i.e., practical).

**FIGURE 2:**
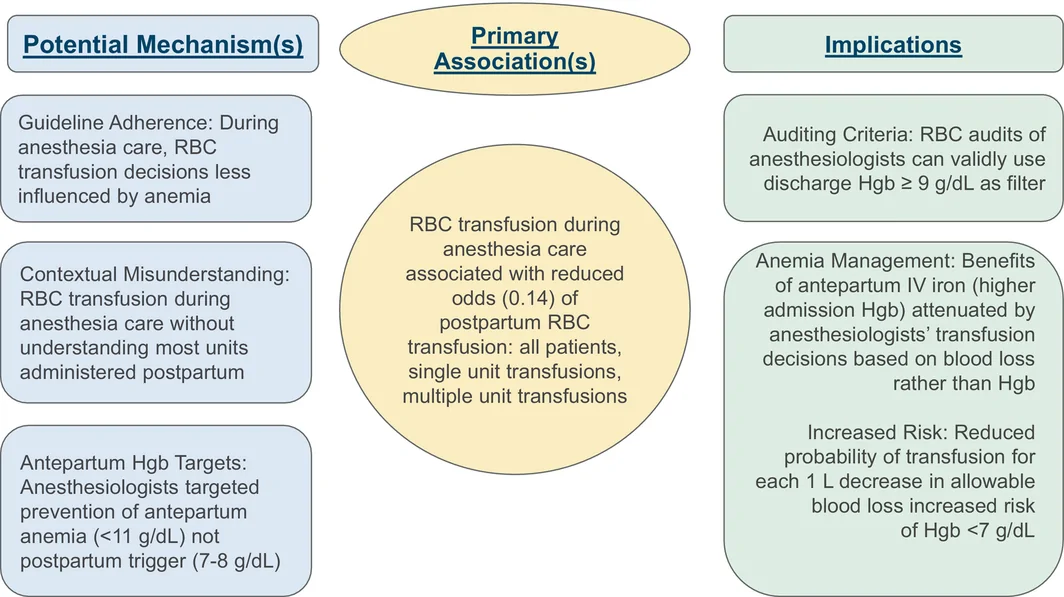
Mechanisms (left), primary association (center), and implications (right) of our retrospective cohort study. The multiple results paragraphs are represented visually. The attenuation of the benefit of antepartum anaemia management and greater risk of severe anaemia are combined to the right because they are the same consequence of reduced sensitivity to the admission haemoglobin. In the figure, Hgb is abbreviation for haemoglobin.

A second implication for anaesthesia departments involved in antepartum anaemia management (Appendices S5 and S6) is that the direct^21^ benefit of increasing the admission haemoglobin to reduce the probability of RBC transfusion (Figure 1) was attenuated by RBC transfusion during anaesthesia care when blood loss was <2500 mL (Appendix S1). A counterargument could be that transfusion during anaesthesia care resulted in a few unnecessary units but provided a greater benefit of preventing severe anaemia in some patients. That argument is *invalid* based on the multivariable analyses showing the *opposite* applied (*p* < 0.0001), specifically the absence of transfusion among patients with allowable blood loss^12^ <2500 mL. Another counterargument could be that RBC transfusion during anaesthesia care had no net negative effect because it prevented comparable units from being transfused postpartum. That argument is also *invalid* based on patients without RBC transfusion during anaesthesia care having *lower* discharge haemoglobins (*p* < 0.0001).

### 4.1 Validity and generalizability

Six associations from earlier studies^11^, ^19^, ^20^, ^21^, ^22^, ^23^, ^24^, ^25^ suggest the predictive validity of our cohort. First, our observed incidence of at least one RBC unit transfused during anaesthesia care was comparable to that reported in “operating rooms” elsewhere (Table 1).^19^ Second, our incidence of single‐unit RBC transfusion was comparable to that observed after a period of clinician education on choosing wisely (Table 1).^20^ Third, there was an inverse association between haemoglobin at admission before delivery and blood loss during delivery as reported earlier (Appendix S3).^21^ Fourth, there was a positive association between admission and discharge haemoglobin (Appendix S4).^14^, ^22^ Fifth, there was a positive association between haemoglobin at admission before delivery and the frequency of RBC transfusion (Figure 1).^24^, ^25^ Finally, approximately 14% of RBC units were transfused three or more days after delivery, similar to earlier observations (Table 2).^11^

Several observations also show external validity (generalizability) of the cohort. Some earlier studies have reported greater incidences of intrapartum RBC transfusion than our observed 4.4% of deliveries (e.g., 7.4% at a university hospital in Nigeria).^28^ Conversely, some studies have reported substantially lesser incidences of RBC transfusion (e.g., 1.5% at a maternity hospital in Ireland^20^ and 1.2% among all deliveries in a hospital in Germany over 9 years^29^). Some other studies reported rates more similar to ours (e.g., 3.2% at a tertiary obstetric hospital in Singapore and 4.9% in India).^19^, ^30^ Substantial variability in the incidence of intrapartum transfusions among hospitals throughout British Columbia has been reported previously.^31^ Anaesthesiologists working at hospitals with much lower incidences of transfusion than ours should consider even greater hesitancy for transfusion during delivery until the postpartum haemoglobin is known (i.e., for such hospitals, our results are even more reliable).

### 4.2 Limitations

Our study's single center had RBC transfusion practices that differed from some cohorts. For example, at a university hospital in Turkey, 9% (20/213) of intrapartum patients received RBC transfusion “during the delivery.”^27^ Regardless of how defined, this would be a smaller percentage of patients than in our cohort (Table 2). Their 79% of patients with transfusion postpartum was also larger than our cohort.^27^ For anaesthesiologists practicing in such a setting, the implications would be of even *greater* importance: when managing obstetrical haemorrhage, the antenatal haemoglobin should be considered when making transfusion decisions, unlike our department's original policy (Appendix S1). Potentially, the policy could be revised so that the allowable blood loss is calculated based on the admission haemoglobin and discussed at the pre‐procedure briefing or timeout.^12^

An earlier randomised trial of single‐unit versus multiple‐unit RBC transfusions administered postpartum showed that, with single‐unit transfusion, patient discharge with severe anaemia was not associated with significantly greater tachycardia, lightheadedness, fatigue, or maternal length of stay.^15^ The mean haemoglobin from the cohort with single unit RBC transfusion (7.8 g/dL) was less than the discharge haemoglobin among the patients in the current study (Table 3). This result further supports the safety of waiting for a haemoglobin as low as 7 g/dL postpartum before RBC transfusion (i.e., the bias in our retrospective cohort was toward RBC transfusion during anaesthesia care, not vice versa).

## 5 CONCLUSIONS

Our study was novel in having a large dataset of both the times of antepartum and postpartum RBC transfusion and the times of anaesthesia care for delivery (Appendix S2). Unlike for several categories of non‐obstetrical^3^, ^4^, ^5^, ^6^, ^7^, ^8^, ^9^ procedures, RBC transfusion during anaesthesia care for delivery was associated with reduced probability of postpartum RBC transfusion. The association applied not only overall but for single and multiple RBC units transfused. Although several explanations were consistent with the data, and may have each applied somewhat, the consequences would be the same. Any one or a combination of the three mechanisms studied would result in the direct benefits of antepartum anaemia treatment in increasing admission haemoglobin and lowering RBC transfusion risk to be mitigated by resuscitation practices for haemorrhage during anaesthesia care. Furthermore, the omissions of admission haemoglobins from transfusion decisions were associated with anaesthesiologists missing the identification of multiple patients with severe anaemia and low allowable blood loss. The earlier findings, for both non‐obstetric and obstetric patients, held, specifically that decisions to audit anaesthesiologists' RBC transfusion decisions can reasonably be based not only on the blood loss (<2500 mL) but also the discharge haemoglobin (e.g., ≥9 g/dL).^1^

## AUTHOR CONTRIBUTIONS

F.D. helped with conceptualization, methodology, software, validation, formal analysis, investigation, data curation, writing original draft, writing review & editing, and visualisation. R.H.E. helped with validation, writing review & editing, and visualisation. U.S.I. helped with conceptualization, methodology, investigation, resources, writing review & editing, and project administration.

## FUNDING INFORMATION

No funding was received for this research.

## CONFLICT OF INTEREST STATEMENT

The authors declare no conflicts of interest.

## Supporting information


**APPENDIX S1:** University of Iowa Obstetric haemorrhage management guide, version 2.0 updated October 2022.
**APPENDIX S2:** PubMed search evaluating earlier studies on the timing of intrapartum RBC transfusion.
**APPENDIX S3:** Characteristics of studied deliveries, vaginal or caesarean delivery, with or without an anaesthesiologist, among patients with haemoglobin determinations on admission or within 1 week before admission.
**APPENDIX S4:** Characteristics of studied deliveries, vaginal or caesarean delivery, with or without an anaesthesiologist, among patients with haemoglobin determinations both on admission and before discharge.
**APPENDIX S5:** First antenatal haemoglobin <11 g/dL was associated with greater probability of RBC transfusion during the hospitalisation that included the delivery.
**APPENDIX S6:** Intravenous iron infusions starting even 4–6 weeks before delivery were associated with significantly reduced incidences of anaemia at admission for delivery.

**APPENDIX S7:** S7 Stata code and log v45.pdf.

## Data Availability

We have included supplemental file “S7 Stata code and log v45.pdf”. The DTA data files are available after data use agreement with the University of Iowa.
