# Supplementary material for: Timing of intrapartum transfusion among obstetric patients, stratified by admission and discharge haemoglobin, relative to anaesthesia care: A retrospective cohort study
Source: Transfus Med. 2026 Apr 26;36(5):475–83. doi: 10.1111/tme.70082 (PMC13670405; doi:10.1111/tme.70082)
Supplement: Supplementary file 1 [file TME-36-475-s001.pdf]

```
log using     "01 OB anemia clinic Transfusion Log"    , replace

version 19.5
set more off



*** Table 1 *** (all patients) ***********************************************************************************
use "Temp_Table 1.dta", clear

describe, fullnames

summarize MaternalAge, detail
summarize Parity     , detail

generate bCesarean = 0
replace  bCesarean = 1 if AnesHCPCS == 59510 | AnesHCPCS == 59514 | AnesHCPCS == 59515 | ///
                          AnesHCPCS == 59525 | AnesHCPCS == 59620 | AnesHCPCS == 59622
tabulate bCesarean // many of the "0" are without anesthesia clinician

generate double HoursBeforeDelivery = (Delivery - Hosp_Adm)/ (1000*60*60)
summarize       HoursBeforeDelivery, detail

generate double HoursAfterDelivery  = (Hosp_DC - Delivery) / (1000*60*60)
summarize       HoursAfterDelivery, detail

codebook AnesHCPCS if bAnesDeliv == 1 & AnesHCPCS == .
codebook AnesHCPCS if bAnesDeliv == 0
tabulate AnesHCPCS if AnesHCPCS == ., missing
display %4.0f 100*(1-r(N)/_N) "% (" _N-r(N) "/" _N ")"

tabulate bAnesDeliv

tabulate Any_RBC_units // use manually for eighth paragraph of results, % units admission hemoglobin known or not
tabulate Any_RBC_Anes
tabulate Any_RBC_Anes if Blood_loss < 2500 // last sentence eighth paragraph Results

tabulate RBC_GE2
tabulate RBC_GE2_Anes

summarize N_units      if Any_RBC_units > 0, detail
summarize N_units_Anes if Any_RBC_Anes > 0, detail

summarize QuanBloodLoss, detail
summarize Blood_loss   , detail



*** Table S3 = Table 1 version B, patients with initial (admission) Hgb ******************************************
use "Temp_Table S3.dta", clear

describe, fullnames

```

```
generate long  Blood_loss = .
replace        Blood_loss = QuanBloodLoss if QuanBloodLoss != .
replace        Blood_loss = EstBloodLoss  if QuanBloodLoss == . & EstBloodLoss != .
label variable Blood_loss "Quantitative else estimated blood loss at delivery, mL"

summarize MaternalAge, detail
summarize Parity     , detail

generate bCesarean = 0
replace  bCesarean = 1 if AnesHCPCS == 59510 | AnesHCPCS == 59514 | AnesHCPCS == 59515 | ///
                          AnesHCPCS == 59525 | AnesHCPCS == 59620 | AnesHCPCS == 59622
tabulate bCesarean

generate double HoursBeforeDelivery = (Delivery - Hosp_Adm)/ (1000*60*60)
summarize       HoursBeforeDelivery, detail

generate double HoursAfterDelivery  = (Hosp_DC - Delivery) / (1000*60*60)
summarize       HoursAfterDelivery, detail

codebook AnesHCPCS if bAnesDeliv == 1 & AnesHCPCS == .
codebook AnesHCPCS if bAnesDeliv == 0
tabulate AnesHCPCS if AnesHCPCS == ., missing
display %4.0f 100*(1-r(N)/_N) "% (" _N-r(N) "/" _N ")"

tabulate bAnesDeliv

tabulate Any_RBC_units // used manually for eighth paragraph of results, % units admission hemoglobin known or not
tabulate Any_RBC_Anes
tabulate Any_RBC_Anes if Blood_loss < 2500 // last sentence eighth paragraph Results

tabulate RBC_GE2
tabulate RBC_GE2_Anes

summarize N_units      if Any_RBC_units > 0, detail
summarize N_units_Anes if Any_RBC_Anes  > 0, detail

summarize QuanBloodLoss , detail
summarize Blood_loss    , detail
summarize AllowBloodLoss, detail

summarize Hgb_Adm      , detail

generate byte bHgb_Adm_GT_9 = .
replace       bHgb_Adm_GT_9 = 1 if Hgb_Adm > 9 & Hgb_Adm != .
replace       bHgb_Adm_GT_9 = 0 if Hgb_Adm <=9 & Hgb_Adm != .
tabulate      bHgb_Adm_GT_9 // Table S3 (1B), footnote d last sentence; 2nd to last paragraph discussion

generate byte  bAnemic = 0
replace        bAnemic = 1 if Hgb_Adm < 11
label variable bAnemic  "1 if Hgb_Adm < 11 g/dL, 0 otherwise"
tabulate       bAnemic

by bAnemic, sort : summarize Blood_loss // Table S3, footnote c; anemic patients had greater blood loss
```

```
ranksum Blood_loss, by(bAnemic) porder



*** Table S4 = Table 1 version C, patients with initial (admission) and final (discharge) Hgb ********************
use "Temp_Table S4.dta", clear

describe, fullnames

summarize MaternalAge, detail
summarize Parity     , detail

generate bCesarean = 0
replace  bCesarean = 1 if AnesHCPCS == 59510 | AnesHCPCS == 59514 | AnesHCPCS == 59515 | ///
                          AnesHCPCS == 59525 | AnesHCPCS == 59620 | AnesHCPCS == 59622
tabulate bCesarean

generate double HoursBeforeDelivery = (Delivery - Hosp_Adm)/ (1000*60*60)
summarize       HoursBeforeDelivery, detail

generate double HoursAfterDelivery  = (Hosp_DC - Delivery) / (1000*60*60)
summarize       HoursAfterDelivery, detail

codebook AnesHCPCS if bAnesDeliv == 1 & AnesHCPCS == .
codebook AnesHCPCS if bAnesDeliv == 0
tabulate AnesHCPCS if AnesHCPCS == ., missing
display %4.0f 100*(1-r(N)/_N) "% (" _N-r(N) "/" _N ")"

tabulate bAnesDeliv

tabulate Any_RBC_units
tabulate Any_RBC_Anes

tabulate RBC_GE2
tabulate RBC_GE2_Anes

summarize N_units      if Any_RBC_units > 0, detail
summarize N_units_Anes if Any_RBC_Anes > 0, detail

summarize QuanBloodLoss , detail
summarize Blood_loss    , detail
summarize AllowBloodLoss, detail

summarize Hgb_Adm, detail

generate byte bAnemic_Adm = 0
replace       bAnemic_Adm = 1 if Hgb_Adm < 11
tabulate bAnemic_Adm

summarize Hgb_DC , detail

generate byte bAnemic_DC = 0
replace       bAnemic_DC = 1 if Hgb_DC <9
```

```
tabulate bAnemic_DC

generate byte bAnemic_Severe_DC = 0
replace       bAnemic_Severe_DC = 1 if Hgb_DC < 8
tabulate      bAnemic_Severe_DC

cs bAnemic_DC bAnemic_Adm, or exact level(99)  // Aghajanian S et al. 2024 prediction, Table S4 footnote d



*** First paragraph of Results, using same file as for Table 1 ***************************************************
use "Temp_Table 1.dta", clear

describe, fullnames

keep PAT_MRN_ID Delivery
sort PAT_MRN_ID Delivery
bysort PAT_MRN_ID: egen first_delivery = min(Delivery)
bysort PAT_MRN_ID: egen last_delivery  = max(Delivery)
generate double delivery_diff_years = (last_delivery - first_delivery)/ (1000 * 60 * 60 * 24 * 365)
replace         delivery_diff_years = . if PAT_MRN_ID == PAT_MRN_ID[_n-1]
format %4.1f    delivery_diff_years

* codebook       PAT_MRN_ID                // for sentence in Results
quietly levelsof PAT_MRN_ID, local(levels) // same, but without displaying sample observations
local ndistinct : word count `levels'
display "Distinct values in PAT_MRN_ID: `ndistinct'"

summarize       delivery_diff_years if delivery_diff_years > 0, detail



*** Second paragraph of the Results ******************************************************************************
use "Temp_Table 1.dta", clear

ratio (N_units_Anes/N_units) if N_units > 0, level(99) // also seventh paragraph Results
display "Among all peripartum RBC units, " %4.1f 100*r(table)[1,1] "% are transfused during anesthesia care."
summarize N_units_Anes       if N_units > 0
display "Total for numerator of fraction = "   r(N)*r(mean)
summarize N_units            if N_units > 0
display "Total for denominator of fraction = " r(N)*r(mean)

clonevar Any_RBC_Not_Anes = Any_RBC_units
assert   N_units - N_units_Anes >= 0
replace  Any_RBC_Not_Anes = 0 if N_units - N_units_Anes == 0
tabulate Any_RBC_Anes Any_RBC_Not_Anes
mcc      Any_RBC_Anes Any_RBC_Not_Anes, level(99) // RBC during anesthesia care reduces probability subsequent transfusion
display %5.4f 3*r(p_exact)

clonevar RBC_1_Anes = Any_RBC_Anes
replace  RBC_1_Anes = 0 if RBC_GE2_Anes == 1
tabulate RBC_1_Anes Any_RBC_Not_Anes
mcc      RBC_1_Anes Any_RBC_Not_Anes, level(99) // applies to one unit by anesthesia
```

```
display %5.4f 3*r(p_exact)

clonevar RBC_GE2_Not_Anes = RBC_GE2
replace  RBC_GE2_Not_Anes = 0 if N_units - N_units_Anes < 2
tabulate RBC_GE2_Anes RBC_GE2_Not_Anes
mcc      RBC_GE2_Anes RBC_GE2_Not_Anes, level(99) // and to two or more units
display %5.4f 3*r(p_exact)



*** Third paragraph of the Results *******************************************************************************
use "Temp_Table S3.dta", clear

describe, fullnames

generate byte bAnemic_Adm = 0
replace       bAnemic_Adm = 1 if Hgb_Adm < 11
tabulate bAnemic_Adm // Figure 1 legend

generate int N_units_not_Anes = N_units - N_units_Anes

ranksum                          N_units,          by(bAnemic_Adm) porder
ranksum                          N_units_not_Anes, by(bAnemic_Adm) porder // Figure 1 legend
by bAnemic_Adm, sort : summarize N_units
ranksum                          N_units_Anes,     by(bAnemic_Adm) porder // Figure 1 legend
by bAnemic_Adm, sort : summarize N_units_Anes

keep    Any_RBC_units PAT_MRN_ID                   Any_RBC_Anes bAnemic_Adm
assert (Any_RBC_units == 0 | Any_RBC_units ==1) & (Any_RBC_Anes == 0 | Any_RBC_Anes == 1)

generate int identifier_admission = _n // not using PAT_MRN_ID because >1 delivery for some patients

generate byte Any_RBC_notAnes = 0
replace       Any_RBC_notAnes = 1 if Any_RBC_units == 1 & Any_RBC_Anes == 0
drop                                 Any_RBC_units

reshape long Any_RBC_, i(identifier_admission) j(group) string
rename       Any_RBC_ bAny_RBC

encode group, gen(group_num)
codebook          group_num // 1 Anes

sort  PAT_MRN_ID identifier_admission group_num  // Results, secondary analyses, paragraph 1
xtgee bAny_RBC i.bAnemic_Adm##i.group_num, family(binomial) link(logit) corr(exchangeable) i(PAT_MRN_ID) robust
margins, dydx(bAnemic_Adm) over(group_num)                    // probabilities for text
margins, dydx(bAnemic_Adm) over(group_num) pwcompare(effects) // P-value



*** Fourth paragraph of the Results, second secondary analyses paragraph *****************************************
use "Temp_Table S3.dta", clear

spearman AllowBloodLoss Hgb_Adm
```

```

generate byte Any_RBC_NotAnes = 0
replace       Any_RBC_NotAnes = 1 if N_units > N_units_Anes

summarize AllowBloodLoss
generate double ABL_Centered = ( AllowBloodLoss - r(mean) )/1000
label var       ABL_Centered "ABL (Liters) centered at mean"

generate int identifier_admission = _n

keep PAT_MRN_ID Any_RBC_Anes Any_RBC_NotAnes ABL_Centered identifier_admission

reshape long Any_RBC_, i(identifier_admission) j(group) string
rename       Any_RBC_ bRBC
encode group, gen(group_num) // 1 = Anes, 2 = NotAnes

sort  PAT_MRN_ID identifier_admission group_num
xtgee bRBC i.group_num##c.ABL_Centered, family(binomial) link(logit) corr(exchangeable) i(PAT_MRN_ID) robust nolog

margins, dydx(ABL_Centered) over(group_num) post // slopes per 1 liter allowable blood loss
nlcom (Fold_Difference: _b[2.group_num] / _b[1.group_num]), level(99)
matrix list r(table) // for the P-value



*** Fifth paragraph of the Results, fourth paragraph of the secondary analyses ***********************************
use "Temp_Table S3.dta", clear

drop if  Any_RBC_units == 0

generate byte bAnemic = 0
replace       bAnemic = 1 if Hgb_Adm < 11

clonevar N_units_Anes_Not_Anemic = N_units_Anes
replace  N_units_Anes_Not_Anemic = 0 if bAnemic == 1
ratio (N_units_Anes_Not_Anemic/N_units_Anes), level(99)
display "Among all peripartum RBC units started during anesthesia care, for " %4.1f 100*r(table)[1,1] ///
        "% of units, the patient was not anemic (Hgb ≥ 11 g/dL) upon admission."
summarize N_units_Anes_Not_Anemic
display "Total for numerator of fraction = "   r(N)*r(mean)
summarize N_units_Anes
display "Total for denominator of fraction = " r(N)*r(mean)

tabulate bAnesTrans bAnemic, row



*** Table 2, timing of RBC transfusion among all patients, seventh paragraph results  ****************************
use "Temp_Table 2.dta", clear

describe, fullnames

assert _N == 30591 // deliveries
```

```
joinby PAT_MRN_ID using "Temp_Transfusions", unmatched(both) // RBC transfusion data
drop if    _merge == 1

assert RBCtime != . // because have deleted the patients without transfusion
drop if (RBCtime < Hosp_Adm | RBCtime > Hosp_DC) // records joined and from different admissions (deliveries)

sort PAT_MRN_ID Hosp_Adm RBCtime
generate double RBCtimeHr = (RBCtime - Delivery)/ (1000*60*60)
label variable  RBCtimeHr "RBC unit transfused relative to time of delivery"

recode RBCtimeHr (min/-1 = 1) (-1/2 = 2) (2/4 = 3) (4/12 = 4) (12/24 = 5) (24/48 = 6) (48/max = 7), gen(RBCHrCat)
label define RBCHrCat_labels  ///
    1 "≤-1 hr before"         ///
    2 ">-1 to  ≤2 hr"         ///
    3 ">2 to  ≤4 hr"          ///
    4 ">4 to ≤12 hr"          ///
    5 ">12 to ≤24 hr"         ///
    6 ">24 to ≤48 hr"         ///
    7 ">48 hr after", modify   // labels with modify to prevent error that already defined
label values RBCHrCat RBCHrCat_labels
tabulate     RBCHrCat // units, not patients
tabulate     RBCHrCat if bAnesTrans == 0
tabulate     RBCHrCat if bAnesTrans == 1

generate byte  bLE1BeforeORGT4After = 0 if RBCHrCat != .
replace        bLE1BeforeORGT4After = 1 if RBCHrCat != . & (RBCHrCat == 1 | RBCHrCat > 3)
ci proportions bLE1BeforeORGT4After, level(99)

display "Among all peripartum RBC units, " %4.0f 100*r(proportion) "% (" r(proportion)*r(N) ///
        "/" r(N) ")" _n "are transfused ≤-1 hr before or >4 hr after delivery."              // _n, line break

ci proportions bLE1BeforeORGT4After if bAnesTrans == 1, level(99) // Table 2 footnote e
display "Among all peripartum RBC units during anesthesia care, " %4.0f 100*(1-r(proportion)) "% ("  ///
        (1-r(proportion))*r(N) "/" r(N) ")" _n "are transfused ≤-1 hr before or >4 hr after delivery."
    * seventh paragraph Results (Table 2)

sort PAT_MRN_ID Hosp_Adm RBCtime // repeating by patient rather than preceding by unit
                                 // however in the table presented as patients first
replace bLE1BeforeORGT4After = . if PAT_MRN_ID == PAT_MRN_ID[_n-1] & Hosp_Adm == Hosp_Adm[_n-1]
replace              RBCHrCat  = . if PAT_MRN_ID == PAT_MRN_ID[_n-1] & Hosp_Adm == Hosp_Adm[_n-1]
replace              RBCtimeHr = . if PAT_MRN_ID == PAT_MRN_ID[_n-1] & Hosp_Adm == Hosp_Adm[_n-1]
label variable       RBCtimeHr "RBC first unit transfused relative to time of delivery"

tabulate     RBCHrCat                     // patients
tabulate     RBCHrCat if bAnesTrans == 0
tabulate     RBCHrCat if bAnesTrans == 1 // counts not same as Table 1 because using first unit

ci proportions bLE1BeforeORGT4After, level(99)
display "Among peripartum patients receiving ≥1 RBC unit, " %4.1f 100*r(proportion) ///
        "% (" r(proportion)*r(N) "/" r(N) ")"                                       ///
        _n "receive their first (or only) unit ≤-1 hr before or >4 hr after delivery"

ci proportions bLE1BeforeORGT4After if bAnesTrans == 1, exact level(99)
```

```
display "Among peripartum patients receiving ≥1 RBC unit during anesthesia care, " %4.1f 100*r(proportion) ///
        "% (" r(proportion)*r(N)                                                                    ///
        "/" r(N) ")" _n "receive their first (or only) unit ≤-1 hr before or >4 hr after delivery"



*** Table 3, patients with Hgb initially and after delivery, eighth paragraph results  ***************************
use "Temp_Table S4.dta", clear

describe, fullnames

clonevar Any_RBC_Not_Anes = Any_RBC_units
assert   N_units - N_units_Anes >= 0
replace  Any_RBC_Not_Anes = 0 if N_units - N_units_Anes == 0

generate byte bHgb_DC_LT_9 = 0
replace       bHgb_DC_LT_9 = 1 if Hgb_DC <9

summarize Hgb_Adm      if Any_RBC_Anes == 0 & Any_RBC_Not_Anes == 0, detail
summarize Hgb_DC       if Any_RBC_Anes == 0 & Any_RBC_Not_Anes == 0, detail
tabulate  bHgb_DC_LT_9 if Any_RBC_Anes == 0 & Any_RBC_Not_Anes == 0
summarize Blood_loss   if Any_RBC_Anes == 0 & Any_RBC_Not_Anes == 0, detail

summarize Hgb_Adm      if Any_RBC_Anes == 0 & Any_RBC_Not_Anes == 1, detail
summarize Hgb_DC       if Any_RBC_Anes == 0 & Any_RBC_Not_Anes == 1, detail
tabulate  bHgb_DC_LT_9 if Any_RBC_Anes == 0 & Any_RBC_Not_Anes == 1
summarize Blood_loss   if Any_RBC_Anes == 0 & Any_RBC_Not_Anes == 1, detail

summarize Hgb_Adm      if Any_RBC_Anes == 1 & Any_RBC_Not_Anes == 1, detail
summarize Hgb_DC       if Any_RBC_Anes == 1 & Any_RBC_Not_Anes == 1, detail
tabulate  bHgb_DC_LT_9 if Any_RBC_Anes == 1 & Any_RBC_Not_Anes == 1
summarize Blood_loss   if Any_RBC_Anes == 1 & Any_RBC_Not_Anes == 1, detail

summarize Hgb_Adm      if Any_RBC_Anes == 1 & Any_RBC_Not_Anes == 0, detail
summarize Hgb_DC       if Any_RBC_Anes == 1 & Any_RBC_Not_Anes == 0, detail
tabulate  bHgb_DC_LT_9 if Any_RBC_Anes == 1 & Any_RBC_Not_Anes == 0
summarize Blood_loss   if Any_RBC_Anes == 1 & Any_RBC_Not_Anes == 0, detail

display "Sum of denominators 3809 + 751 + 127 + 130 = " 3809 + 751 + 127 + 130 " = 4817 = " _N // footnote a

summarize Hgb_DC if Any_RBC_Not_Anes == 1 // footnote to compare with Hamm RF et al. 2021, Footnote b

generate byte bHgb_DC_GE_9 = . // first sentence of results, eighth paragraph
replace       bHgb_DC_GE_9 = 1 if Hgb_DC >= 9 & Hgb_DC != .
replace       bHgb_DC_GE_9 = 0 if Hgb_DC <   9 & Hgb_DC != .
tabulate Any_RBC_Not_Anes bHgb_DC_GE_9 if Any_RBC_Anes == 1, exact row

capture drop  bHgb_DC_GE_9
generate byte bHgb_DC_GE_9 = . // Variable used in the Results section
replace       bHgb_DC_GE_9 = 0 if Any_RBC_Anes == 1 & Any_RBC_Not_Anes == 0
replace       bHgb_DC_GE_9 = 1 if Any_RBC_Anes == 1 & Any_RBC_Not_Anes == 0 & Hgb_DC >= 9
tabulate      bHgb_DC_GE_9                          // results eighth paragraph
```

```
ranksum  N_units_Anes, by(bHgb_DC_GE_9) porder
tabulate N_units_Anes if  bHgb_DC_GE_9 == 0
tabulate N_units_Anes if  bHgb_DC_GE_9 == 1         // larger % single unit (i.e., cannot explain)

ranksum   Blood_loss, by (bHgb_DC_GE_9) porder
summarize Blood_loss if   bHgb_DC_GE_9 == 0, detail // larger
summarize Blood_loss if   bHgb_DC_GE_9 == 1, detail

ranksum   Hgb_Adm, by(bHgb_DC_GE_9) porder          // results eighth paragraph "the admission hemoglobin"
summarize Hgb_Adm if  bHgb_DC_GE_9 == 0, detail
summarize Hgb_Adm if  bHgb_DC_GE_9 == 1, detail     // explains, and matters more becuase infusion clinic

generate bCesarean = 0
replace  bCesarean = 1 if AnesHCPCS == 59510 | AnesHCPCS == 59514 | AnesHCPCS == 59515 | ///
                          AnesHCPCS == 59525 | AnesHCPCS == 59620 | AnesHCPCS == 59622

tabulate bCesarean if Any_RBC_Anes == 1 & Any_RBC_Not_Anes == 0
tabulate bCesarean if Any_RBC_Anes == 1 & Any_RBC_Not_Anes == 0 & bHgb_DC_GE_9 == 1
tabulate bCesarean if Any_RBC_Anes == 1 & Any_RBC_Not_Anes == 0 & bHgb_DC_GE_9 == 1 & ///
                      Blood_loss < 2500 & Blood_loss != . & N_units == 1 // eighth paragraph results
summarize N_units
display r(mean)*r(N)
summarize N_units if bCesarean == 1 & Any_RBC_Anes == 1 & Any_RBC_Not_Anes == 0 & bHgb_DC_GE_9 == 1 & ///
                     Blood_loss < 2500 & Blood_loss != . & N_units == 1
display r(mean)*r(N)
display %4.1f 100*36/2065 "% (36/2065) units"

* Repeating among patients with discharge hemoglobin >= 11
tabulate bCesarean if Any_RBC_Anes == 1 & Any_RBC_Not_Anes == 0 & Hgb_DC >= 11
tabulate bCesarean if Any_RBC_Anes == 1 & Any_RBC_Not_Anes == 0 & Hgb_DC >= 11 & ///
                      Blood_loss < 2500 & Blood_loss != . & N_units == 1



*** Ninth paragraph of Results, audit criteria *******************************************************************
use "Temp_Table 1.dta" , clear
tabulate bAnesDeliv // because audit analysis is proportion of cases

use "Temp_Table S4.dta", clear
assert Hgb_Adm != . & Hgb_DC != .

tabulate bAnesDeliv if (N_units > 0) & (N_units_Anes == N_units) & (Hgb_DC >= 9)
display %4.1f 100* 104/20887 "% (104/20,887)"

tabulate bAnesDeliv if (N_units > 0) & (N_units_Anes == N_units) & (Hgb_DC >= 9) & (Blood_loss < 2500)
display %4.1f 100* 74/20887 "% (74/20,887)"
tabulate N_units    if (N_units > 0) & (N_units_Anes == N_units) & (Hgb_DC >= 9) & (Blood_loss < 2500)



*** Figure 1 and legend *******************************************************************************************
use "Temp_Table S3.dta", clear
```

```
capture drop Perc - CI_high_perc
generate double Perc = .          // percentage any transfusion
generate double Logit = .
generate double CI_low_perc  = .
generate double CI_high_perc = .
forvalues i = 1/10 {
    quietly ci proportions Any_RBC_units if Hgb_Decile == `i', wilson level(99)
    quietly replace Perc = 100*   r(proportion)  if Hgb_Decile == `i' & Hgb_Decile != Hgb_Decile[_n-1]
    quietly replace Logit = logit(r(proportion)) if Hgb_Decile == `i' & Hgb_Decile != Hgb_Decile[_n-1]
    quietly replace CI_low_perc  = 100*r(lb)     if Hgb_Decile == `i' & Hgb_Decile != Hgb_Decile[_n-1]
    quietly replace CI_high_perc = 100*r(ub)     if Hgb_Decile == `i' & Hgb_Decile != Hgb_Decile[_n-1]
}

twoway (scatter Perc MedianHgb, msymbol(O) mcolor(blue) msize(large))                           ///
       (rcap CI_low_perc CI_high_perc MedianHgb, lcolor(ltblue) lwidth(medthick))               ///
       (scatter Logit MedianHgb, msymbol(o) mfcolor(red%50) mlcolor(red) yaxis(2) msize(large)) ///
       (lfit Logit MedianHgb, lcolor(red) lwidth(medium) yaxis(2)),                             ///
       ylabel(0(2)18) xlabel(9.5(0.5)14.5, format(%4.1f)) legend(off)                           ///
       ytitle("Percentage (%) of patients with RBC transfusion", color(blue))                   ///
       xtitle("Median hemoglobin (g/dL) at admission for delivery")                             ///
       ytitle("Logit of proportion of patients with RBC transfusion", color(red) axis(2))       ///
       ylabel(-5.0(0.5)-1.5, format(%4.1f) axis(2))
graph export  "02 OB anemia clinic Transfusion Log.pdf", as(pdf) name("Graph") replace // currently not in paper

capture log close
translate     "01 OB anemia clinic Transfusion Log.smcl" ///
              "01 OB anemia clinic Transfusion Log.pdf", replace
capture erase "01 OB anemia clinic Transfusion Log.smcl"
log using     "03 OB anemia clinic Transfusion Log"    , replace

* Graph that shows population percentage on left, as above, with % during anesthesia care on right
capture drop Perc - CI_high_perc
generate double Perc  = .
generate double PercA = .
generate double CI_low_perc   = .
generate double CI_low_percA  = .
generate double CI_high_perc  = .
generate double CI_high_percA = .
forvalues i = 1/10 {

    quietly ci proportions Any_RBC_units if Hgb_Decile == `i', wilson level(99) // note Any_RBC_units
    quietly replace Perc  = 100*   r(proportion)  if Hgb_Decile == `i' & Hgb_Decile != Hgb_Decile[_n-1]
    quietly replace CI_low_perc   = 100*r(lb)     if Hgb_Decile == `i' & Hgb_Decile != Hgb_Decile[_n-1]
    quietly replace CI_high_perc  = 100*r(ub)     if Hgb_Decile == `i' & Hgb_Decile != Hgb_Decile[_n-1]

    quietly ci proportions Any_RBC_Anes  if Hgb_Decile == `i', wilson level(99) // note Any_RBC_Anes, anesthesia units
    quietly replace PercA = 100*   r(proportion)  if Hgb_Decile == `i' & Hgb_Decile != Hgb_Decile[_n-1]
    quietly replace CI_low_percA  = 100*r(lb)     if Hgb_Decile == `i' & Hgb_Decile != Hgb_Decile[_n-1]
    quietly replace CI_high_percA = 100*r(ub)     if Hgb_Decile == `i' & Hgb_Decile != Hgb_Decile[_n-1]
}

clonevar MedianHgbA = MedianHgb
replace  MedianHgbA = MedianHgb + 0.06 if MedianHgb > 13.0
```

```

twoway (scatter Perc MedianHgb, msymbol(O) mcolor(blue) msize(large))                              ///
       (rcap CI_low_perc  CI_high_perc  MedianHgb, lcolor(ltblue) lwidth(medthick))               ///
       (scatter PercA MedianHgbA, msymbol(o) mfcolor(red%50) mlcolor(red) yaxis(2) msize(vlarge))  ///
       (rcap CI_low_percA CI_high_percA MedianHgbA, lcolor(red) yaxis(2) lwidth(medthick)),        ///
       ylabel(0(2)18) xlabel(9.5(0.5)14.5, format(%4.1f)) legend(off)                              ///
       ytitle("Percentage (%) of patients with RBC transfusion", color(blue))                      ///
       xtitle("Median hemoglobin (g/dL) at admission for delivery")                                ///
       ytitle("During anesthesia care" "Percentage (%) of patients with RBC transfusion",          ///
              color(red) axis(2)) ylabel(0(2)18, axis(2))
graph export "Out_Figure 1 v45.jpg"    , as(jpg) name("Graph") replace width(3600) height(2160) quality(100)
graph export  "04 Figure 1 Export.pdf" , as(pdf) name("Graph") replace

capture log close
translate     "03 OB anemia clinic Transfusion Log.smcl" ///
              "03 OB anemia clinic Transfusion Log.pdf", replace
capture erase "03 OB anemia clinic Transfusion Log.smcl"
log using     "05 OB anemia clinic Transfusion Log"    , replace

capture drop Perc - CI_high_perc
generate double Perc = .    // percentage transfusion 2+ units
generate double Logit = .
generate double CI_low_perc  = .
generate double CI_high_perc = .
forvalues i = 1/10 {
    quietly ci proportions RBC_GE2 if Hgb_Decile == `i', wilson level(99)
    quietly replace Perc = 100*   r(proportion)  if Hgb_Decile == `i' & Hgb_Decile != Hgb_Decile[_n-1]
    quietly replace Logit = logit(r(proportion)) if Hgb_Decile == `i' & Hgb_Decile != Hgb_Decile[_n-1]
    quietly replace CI_low_perc  = 100*r(lb)     if Hgb_Decile == `i' & Hgb_Decile != Hgb_Decile[_n-1]
    quietly replace CI_high_perc = 100*r(ub)     if Hgb_Decile == `i' & Hgb_Decile != Hgb_Decile[_n-1]
}

twoway (scatter Perc MedianHgb, msymbol(O) mcolor(blue) msize(large))                             ///
       (rcap CI_low_perc CI_high_perc MedianHgb, lcolor(ltblue) lwidth(medthick))                 ///
       (scatter Logit MedianHgb, msymbol(o) mcolor(red) yaxis(2) msize(large))                    ///
       (lfit Logit MedianHgb, lcolor(red) lwidth(medium) yaxis(2)),                               ///
       ylabel(0(2)14) xlabel(9.5(0.5)14.5, format(%4.1f)) legend(off)                             ///
       ytitle("Percentage (%) of patients with {&ge}2 RBC transfusion", color(blue))              ///
       xtitle("Median hemoglobin (g/dL) at admission for delivery")                               ///
       ytitle("Logit of proportion of patients with {&ge}2 RBC transfusion", color(red) axis(2))  ///
       ylabel(-5.5(0.5)-2.0, format(%4.1f) axis(2))
graph export  "06 OB anemia clinic Transfusion Log.pdf", as(pdf) name("Graph") replace // currently not in paper

capture log close
translate     "05 OB anemia clinic Transfusion Log.smcl" ///
              "05 OB anemia clinic Transfusion Log.pdf", replace
capture erase "05 OB anemia clinic Transfusion Log.smcl"
log using     "07 OB anemia clinic Transfusion Log"    , replace



*** Appendix S5 (Figure C), Initial antenatal Hgb associated with greater Prob(RBC transfusion during delivery) **
use PAT_MRN_ID Hosp_Adm Hosp_DC Delivery using "Temp_Table 2.dta", clear
```

```
display _N " records for later"

* merge with the Hgb file for every possible combination
joinby PAT_MRN_ID using "Temp_Hemoglobins", unmatched(both) // using unmatched to get counts

codebook _merge
drop if  _merge != 3

generate double WkBeforeDeliv = (Delivery - HgbTime)/ (1000*60*60*24*7)
label variable  WkBeforeDeliv "Weeks before delivery"
drop if         WkBeforeDeliv < 0             // because prior delivery
drop if         WkBeforeDeliv > 365*(9/12)/7 //  in data specification plan but was not applied
gsort   PAT_MRN_ID                      Hosp_Adm -WkBeforeDeliv // saving earliest date (i.e., "Hgb" first antepartum)
drop if PAT_MRN_ID == PAT_MRN_ID[_n-1] & Hosp_Adm == Hosp_Adm[_n-1]

drop _merge Delivery HgbTime

joinby PAT_MRN_ID using "Temp_Transfusions", unmatched(both)
codebook _merge
* codebook       PAT_MRN_ID if _merge == 2                // RBC units transfused among patients withOUT Hgb measured
quietly levelsof PAT_MRN_ID if _merge == 2, local(levels) // without displaying some
local ndistinct : word count `levels'
display "Distinct values in PAT_MRN_ID: `ndistinct'"

drop if _merge == 2

drop if (RBCtime < Hosp_Adm | RBCtime > Hosp_DC) & RBCtime != .
sort            PAT_MRN_ID Hosp_Adm RBCtime
by              PAT_MRN_ID Hosp_Adm, sort : egen int N_units = count(RBCtime)
duplicates drop PAT_MRN_ID Hosp_Adm, force

label variable N_units "RBC units transfused among patients with Hgb measured"
assert         N_units != . & N_units >= 0

generate byte Any_RBC_units = 0
replace       Any_RBC_units = 1 if N_units > 0

generate byte RBC_GE2 = 0
replace       RBC_GE2 = 1 if N_units > 1

ranksum Hgb, by(Any_RBC_units) porder // sixth paragraph Results first sentence

xtile Hgb_Decile = Hgb, nq(10)
egen double MedianHgb = median(Hgb), by(Hgb_Decile)
sort        MedianHgb N_units

capture drop Perc - CI_high_perc
generate double Perc = .
generate double Logit = .
generate double CI_low_perc  = .
generate double CI_high_perc = .
forvalues i = 1/10 {
    quietly ci proportions Any_RBC_units if Hgb_Decile == `i', wilson level(99)
```

```
        quietly replace Perc = 100*    r(proportion)  if Hgb_Decile == `i' & Hgb_Decile != Hgb_Decile[_n-1]
        quietly replace Logit = logit(r(proportion)) if Hgb_Decile == `i' & Hgb_Decile != Hgb_Decile[_n-1]
        quietly replace CI_low_perc  = 100*r(lb)     if Hgb_Decile == `i' & Hgb_Decile != Hgb_Decile[_n-1]
        quietly replace CI_high_perc = 100*r(ub)     if Hgb_Decile == `i' & Hgb_Decile != Hgb_Decile[_n-1]
    }

    twoway (scatter Perc MedianHgb, msymbol(O) mcolor(blue) msize(large))                    ///
           (rcap CI_low_perc CI_high_perc MedianHgb, lcolor(ltblue) lwidth(medthick))         ///
           (scatter Logit MedianHgb, msymbol(o) mcolor(red) yaxis(2) msize(large))            ///
           (lfit Logit MedianHgb, lcolor(red) lwidth(medium) yaxis(2)),                       ///
           ylabel(0(2)14) xlabel(9.5(0.5)14.5, format(%4.1f)) legend(off)                     ///
           ytitle("Percentage (%) of patients with RBC transfusion", color(blue))             ///
           xtitle("Median hemoglobin (g/dL) at first antenatal visit")                        ///
           ytitle("Logit of proportion of patients with RBC transfusion", color(red) axis(2)) ///
           ylabel(-4.5(0.5)-1.0, format(%4.1f) axis(2))
    graph export "Out_Figure C v22.jpg"    , as(jpg) name("Graph") replace width(3600) height(2160) quality(100)
    graph export  "08 Figure C Export.pdf", as(pdf) name("Graph") replace

    capture log close
    translate     "07 OB anemia clinic Transfusion Log.smcl" ///
                  "07 OB anemia clinic Transfusion Log.pdf", replace
    capture erase "07 OB anemia clinic Transfusion Log.smcl"
    log using     "09 OB anemia clinic Transfusion Log"    , replace

    summarize WkBeforeDeliv, detail // Supplemental S5 legend, weeks before when first measure Hgb; not infuse, measure Hgb
    spearman  WkBeforeDeliv Hgb     // Supplemental S5 legend comment that correlated

    capture drop Perc - CI_high_perc // Additional figure not in the article
    generate double Perc = .
    generate double Logit = .
    generate double CI_low_perc  = .
    generate double CI_high_perc = .
    forvalues i = 1/10 {
        quietly ci proportions RBC_GE2 if Hgb_Decile == `i', wilson level(99)
        quietly replace Perc = 100*    r(proportion)  if Hgb_Decile == `i' & Hgb_Decile != Hgb_Decile[_n-1]
        quietly replace Logit = logit(r(proportion)) if Hgb_Decile == `i' & Hgb_Decile != Hgb_Decile[_n-1]
        quietly replace CI_low_perc  = 100*r(lb)     if Hgb_Decile == `i' & Hgb_Decile != Hgb_Decile[_n-1]
        quietly replace CI_high_perc = 100*r(ub)     if Hgb_Decile == `i' & Hgb_Decile != Hgb_Decile[_n-1]
    }

    twoway (scatter Perc MedianHgb, msymbol(O) mcolor(blue) msize(large))                            ///
           (rcap CI_low_perc CI_high_perc MedianHgb, lcolor(ltblue) lwidth(medthick))                 ///
           (scatter Logit MedianHgb, msymbol(o) mcolor(red) yaxis(2) msize(large))                    ///
           (lfit Logit MedianHgb, lcolor(red) lwidth(medium) yaxis(2)),                               ///
           ylabel(0(2)18) xlabel(9.5(0.5)14.5, format(%4.1f)) legend(off)                             ///
           ytitle("Percentage (%) of patients with {&ge}2 RBC transfusion", color(blue))              ///
           xtitle("Median hemoglobin (g/dL) at first antenatal visit")                                ///
           ytitle("Logit of proportion of patients with {&ge}2 RBC transfusion", color(red) axis(2)) ///
           ylabel(-5.0(0.5)-1.5, format(%4.1f) axis(2))
    graph export  "10 OB anemia clinic Transfusion Log.pdf", as(pdf) name("Graph") replace // currently not in paper

    capture log close
    translate     "09 OB anemia clinic Transfusion Log.smcl" ///
```

```
                 "09 OB anemia clinic Transfusion Log.pdf", replace
capture erase "09 OB anemia clinic Transfusion Log.smcl"
log using     "11 OB anemia clinic Transfusion Log"    , replace



* Supplemental S6 (Figure D), Sufficient time for Rx at anemia clinic, since only need a few weeks ***************
summarize WkBeforeDeliv, detail

use "Temp_Table S3.dta", clear
joinby PAT_MRN_ID using "Temp_Anemia clinic", unmatched(both)
codebook _merge
drop if  _merge != 3 // data in master and from anemia clinic

generate double FeWkBeforeDeliv = (Delivery - FeTime)/(1000*60*60*24*7)
label variable  FeWkBeforeDeliv "Weeks (double) from Fe infusion to delivery"
drop if         FeWkBeforeDeliv > DeliveryGestAge/7 //  in data specification plan but was not applied
drop if         FeWkBeforeDeliv < 0                 //  another pregnancy
assert          FeWkBeforeDeliv != 0

generate double FeDay = dofc(FeTime)
format          FeDay %td
sort PAT_MRN_ID Hosp_Adm FeDay // required sequence for next command
by   PAT_MRN_ID Hosp_Adm FeDay: generate byte is_unique_Fe_day = (_n == 1)
by PAT_MRN_ID Hosp_Adm, sort: egen n_visits = total(is_unique_Fe_day)
label variable                     n_visits "Count of anemia clinic visits for IV Fe infusion"

by PAT_MRN_ID Hosp_Adm, sort : egen int Total_ml_IV_Fe = total(IV_Fe_mL)
label variable                          Total_ml_IV_Fe "Total ml IV Fe at anemia clinic"

gsort PAT_MRN_ID Hosp_Adm -FeWkBeforeDeliv
drop if PAT_MRN_ID == PAT_MRN_ID[_n-1] & Hosp_Adm == Hosp_Adm[_n-1]

generate double FeStartWkPostConcep = DeliveryGestAge/7 - FeWkBeforeDeliv
format %17.2f   FeStartWkPostConcep
label variable  FeStartWkPostConcep "Fe infusion started these weeks post conception"

drop _merge FeTime IV_Fe_mL DeliveryGestAge FeDay is_unique_Fe_day

summarize n_visits FeWkBeforeDeliv FeStartWkPostConcep, detail // Legend S6 second sentence
* Manually checked the case with smallest week post conception and confirmed (FD 07/19/25)

generate byte  HgbGE11 = 0
replace        HgbGE11 = 1 if Hgb_Adm >= 11
label variable HgbGE11 "Hgb_Adm >=11 g/DL upon admission for delivery & patient Rx IV Fe infusion"

xtile Wk_Quin = FeStartWkPostConcep, nq(5) // Quintile of week post-conception
sort  Wk_Quin   FeStartWkPostConcep
egen double MedianWkPost    = median(FeStartWkPostConcep), by(Wk_Quin)
egen double MedianWkBeforeDeliv = median(FeWkBeforeDeliv), by(Wk_Quin)
replace     MedianWkPost        = . if Wk_Quin == Wk_Quin[_n-1]
replace     MedianWkPost        = round(MedianWkPost, 0.001) // for use in figure upper label below
replace     MedianWkBeforeDeliv = . if Wk_Quin == Wk_Quin[_n-1]
```

```

capture drop Perc - CI_high_erc
generate double Perc = .
generate double Logit = .
generate double CI_low_perc  = .
generate double CI_high_perc = .
forvalues i = 1/5 {
    quietly ci proportions HgbGE11 if Wk_Quin == `i', wilson level(99)
    quietly replace Perc = 100*   r(proportion)  if Wk_Quin == `i' & Wk_Quin != Wk_Quin[_n-1]
    quietly replace Logit = logit(r(proportion)) if Wk_Quin == `i' & Wk_Quin != Wk_Quin[_n-1]
    quietly replace CI_low_perc  = 100*r(lb)     if Wk_Quin == `i' & Wk_Quin != Wk_Quin[_n-1]
    quietly replace CI_high_perc = 100*r(ub)     if Wk_Quin == `i' & Wk_Quin != Wk_Quin[_n-1]
}

preserve // to create temporary data file for the Supplemental S6, Figure D
keep MedianWkPost-CI_high_perc
drop if MedianWkPost == .
save "Figure D.dta", replace
restore

* Figure version currently not used
twoway (scatter Perc MedianWkPost, msymbol(O) mcolor(blue) msize(large))                     ///
       (rcap CI_low_perc CI_high_perc MedianWkPost, lcolor(ltblue) lwidth(medthick))         ///
       (scatter Logit MedianWkPost, msymbol(o) mcolor(red) yaxis(2) msize(large))            ///
       (lfit Logit MedianWkPost, lcolor(red) lwidth(medium) yaxis(2)),                       ///
       ylabel(45(5)85) xscale(reverse) legend(off) ylabel(0(0.2)1.6, format(%4.1f) axis(2))  ///
       ytitle("% patients Hgb {&ge}11 g/dL, upon admission", color(blue))                    ///
       ytitle("Logit of proportion of patients Hgb {&ge}11 g/dL", color(red) axis(2))        ///
       xtitle("First IV Iron infusion date as weeks post-conception" "(as weeks before delivery)") ///
       xlabel(24 "24 (13.7)" 26 "26 (12.0)" 28 "28 (10.2)" 30 "30 (8.4)" 32 "32 (6.7)"       ///
              34 "34 (4.9)" 36 "36 (3.1)" 38 "38 (1.3)") plotregion(margin(r+5))

* Revised S6 Figure from RHE v20250824-1306, primary and secondary horizontal axes' different reverse sequence order
twoway (scatter Perc MedianWkPost, msymbol(O) mcolor(blue) msize(large))           ///
       (rcap CI_low_perc CI_high_perc MedianWkPost, lcolor(blue) lwidth(medium))   ///
       (scatter Logit MedianWkPost, msymbol(o) mcolor(red) yaxis(2) msize(large))  ///
       (lfit Logit MedianWkPost, lcolor(red) lwidth(medium) yaxis(2))              ///
       (pci 85 38 86 38, lcolor(black) lwidth(thin) lpattern(solid))               ///
       (pci 85 36 86 36, lcolor(black) lwidth(thin) lpattern(solid))               ///
       (pci 85 34 86 34, lcolor(black) lwidth(thin) lpattern(solid))               ///
       (pci 85 32 86 32, lcolor(black) lwidth(thin) lpattern(solid))               ///
       (pci 85 30 86 30, lcolor(black) lwidth(thin) lpattern(solid))               ///
       (pci 85 28 86 28, lcolor(black) lwidth(thin) lpattern(solid))               ///
       (pci 85 26 86 26, lcolor(black) lwidth(thin) lpattern(solid))               ///
       (pci 85 24 86 24, lcolor(black) lwidth(thin) lpattern(solid)),              ///
       ylabel(45"45" 50"50" 55"55" 60"60" 65"65" 70"70" 75"75" 80"80" 85"85" 90" ",     ///
              labsize(small)) xscale(reverse) legend(off)                          ///
       ylabel(0.0"0.0" 0.2"0.2" 0.4"0.4" 0.6"0.6" 0.8"0.8" 1.0"1.0" 1.2"1.2" 1.4"1.4" 1.6"1.6" 1.8" ", ///
              labsize(small) format(%4.1f) axis(2))                                ///
       ytitle("% patients Hgb {&ge}11 g/dL, upon admission", color(blue))          ///
       ytitle("Logit of proportion of patients Hgb {&ge}11 g/dL", color(red) axis(2))  ///
       yline(85, lcolor(black) lwidth(thin) lpattern(solid) )                      ///
       xlabel(38(-2)24, labcolor(black) labsize(small))                            ///
```

```
        xtitle("First IV Iron infusion date as weeks post-conception", color(black) size(medium)) ///
        yscale(range(45(5)85) noextend)                  ///
        yscale(range(0(0.2)1.6) axis(2) noextend)        ///
        text(88 37.95 "1.3" , color(black) size(small) ) ///
        text(88 35.95 "3.1" , color(black) size(small) ) ///
        text(88 33.95 "4.9" , color(black) size(small) ) ///
        text(88 31.95 "6.7" , color(black) size(small) ) ///
        text(88 29.95 "8.4" , color(black) size(small) ) ///
        text(88 27.95 "10.2", color(black) size(small) ) ///
        text(88 25.95 "12.0", color(black) size(small) ) ///
        text(88 23.95 "13.7", color(black) size(small) ) ///
        title("First IV Iron infusion date as weeks before delivery", color(black) size(medium)) ///
        plotregion(margin(r+5))
graph export "Out_Figure D v28.jpg"   , as(jpg) name("Graph") replace width(3600) height(2160) quality(100)
graph export  "12 Figure D Export.pdf", as(pdf) name("Graph") replace

capture log close
capture erase "Figure D.dta"
translate     "11 OB anemia clinic Transfusion Log.smcl" ///
              "11 OB anemia clinic Transfusion Log.pdf", replace
capture erase "11 OB anemia clinic Transfusion Log.smcl"
log using     "13 OB anemia clinic Transfusion Log"    , replace

spearman FeWkBeforeDeliv     Hgb_Adm // Supplemental S6 legend and sixth paragraph results second sentence
display "P = " %6.5f r(p)
spearman FeStartWkPostConcep Hgb_Adm
display "P = " %6.5f 4*r(p)

ranksum FeWkBeforeDeliv,     by(HgbGE11) porder // Supplemental S6 legend
display "P = " %6.5f 4*r(p)
ranksum FeStartWkPostConcep, by(HgbGE11) porder
display "P = " %6.5f 4*r(p)

capture log close
translate     "13 OB anemia clinic Transfusion Log.smcl" ///
              "13 OB anemia clinic Transfusion Log.pdf", replace
capture erase "13 OB anemia clinic Transfusion Log.smcl"

```

```
.
. version 19.5

. set more off

.
.
.
. *** Table 1 *** (all patients) ********************************************************************************
. use "Temp_Table 1.dta", clear

.
. describe, fullnames

Contains data from Temp_Table 1.dta
 Observations:        30,160
    Variables:            19                  17 Feb 2026 17:23
------------------------------------------------------------------------------------------------------------------------------------
Variable      Storage   Display    Value
    name         type    format    label      Variable label
------------------------------------------------------------------------------------------------------------------------------------
PAT_MRN_ID      long    %14.0g                Medical record number
Hosp_Adm        double  %tc                   Hospital admission date time
Hosp_DC         double  %tc                   Hospital discharge date time
Delivery        double  %tc                   Delivery date/ time
MaternalAge     byte    %10.0g                Maternal age (years)
Parity          byte    %10.0g                Parity
QuanBloodLoss   long    %12.0g                Quantitative blood loss (ml)
EstBloodLoss    long    %12.0g                Estimated blood loss (ml)
AnesHCPCS       long    %14.0f                CPT for billing anesthesia care
bAnesDeliv      int     %10.0f                Anesthesia care for delivery, 0=No anesthesia clinician
RBCtime         double  %tc                   Date/Time start RBC transfusion
bAnesTrans      int     %10.0f                Anesthesia care when transfusion started
N_units         int     %8.0g                 RBC units transfused
N_units_Anes    int     %8.0g                 RBC units transfused during anesthesia care
Any_RBC_units   byte    %8.0g                 0 or 1 = RBC
Any_RBC_Anes    byte    %8.0g                 0 or 1 = RBC started during anesthesia care
RBC_GE2         byte    %8.0g                 0 or 1 = 2+ units RBC
RBC_GE2_Anes    byte    %8.0g                 0 or 1 = 2+ units RBC started during anesthesia care
Blood_loss      long    %12.0g                Quantitative else estimated blood loss at delivery, mL
------------------------------------------------------------------------------------------------------------------------------------
Sorted by: PAT_MRN_ID  Hosp_Adm  RBCtime

.
. summarize MaternalAge, detail

                    Maternal age (years)
-------------------------------------------------------------
      Percentiles      Smallest
 1%           18             13
 5%           20             14
10%           22             14       Obs              30,160
25%           26             14       Sum of wgt.      30,160

50%           30                      Mean           29.68084
                        Largest       Std. dev.      5.445947
75%           33             51
90%           37             52       Variance       29.65833
95%           39             52       Skewness       .0432921
99%           42             57       Kurtosis       2.792033

. summarize Parity     , detail

                           Parity
-------------------------------------------------------------
      Percentiles      Smallest
 1%            1              1
 5%            1              1
10%            1              1       Obs              30,084
25%            1              1       Sum of wgt.      30,084

50%            2                      Mean           2.130701
                        Largest       Std. dev.       1.27735
75%            3             13
90%            4             13       Variance       1.631622
95%            5             13       Skewness       1.670952
99%            6             14       Kurtosis       7.634932

.
. generate bCesarean = 0

. replace  bCesarean = 1 if AnesHCPCS == 59510 | AnesHCPCS == 59514 | AnesHCPCS == 59515 | ///
>                           AnesHCPCS == 59525 | AnesHCPCS == 59620 | AnesHCPCS == 59622
(10,667 real changes made)

. tabulate bCesarean // many of the "0" are without anesthesia clinician

  bCesarean |      Freq.     Percent        Cum.
------------+-----------------------------------
          0 |     19,493       64.63       64.63
          1 |     10,667       35.37      100.00
------------+-----------------------------------
      Total |     30,160      100.00
```

```
.
. generate double HoursBeforeDelivery = (Delivery - Hosp_Adm)/ (1000*60*60)

. summarize       HoursBeforeDelivery, detail

                     HoursBeforeDelivery
-------------------------------------------------------------
      Percentiles      Smallest
 1%          .15       6.78e-11
 5%     1.683333       6.78e-11
10%     2.577361       2.78e-07       Obs              30,160
25%     4.683333       2.78e-07       Sum of wgt.      30,160

50%     11.03778                      Mean           28.52806
                        Largest       Std. dev.      94.21927
75%     20.95403       1883.367
90%     40.64556       1893.934       Variance       8877.271
95%     71.20833           2023       Skewness       9.640737
99%     472.0667       2115.879       Kurtosis       122.1326

.
. generate double HoursAfterDelivery  = (Hosp_DC - Delivery) / (1000*60*60)

. summarize       HoursAfterDelivery, detail

                     HoursAfterDelivery
-------------------------------------------------------------
      Percentiles      Smallest
 1%     4.216389            1.1
 5%     25.75889       1.133333
10%     30.74833       1.233056       Obs              30,160
25%     40.43333       1.241667       Sum of wgt.      30,160

50%     50.16111                      Mean           53.82158
                        Largest       Std. dev.       31.8233
75%     64.96667       894.2833
90%     76.73306         1010.3       Variance       1012.722
95%     83.64972       1345.833       Skewness        36.9967
99%     127.7925        3161.45       Kurtosis        3182.47

.
. codebook AnesHCPCS if bAnesDeliv == 1 & AnesHCPCS == .

-------------------------------------------------------------------------------------------------------------------
AnesHCPCS                                                                            CPT for billing anesthesia care
-------------------------------------------------------------------------------------------------------------------

                  Type: Numeric (long)

                 Range: [.,.]                        Units: .
         Unique values: 0                        Missing .: 4/4

            Tabulation: Freq.  Value
                            4  .

. codebook AnesHCPCS if bAnesDeliv == 0

-------------------------------------------------------------------------------------------------------------------
AnesHCPCS                                                                            CPT for billing anesthesia care
-------------------------------------------------------------------------------------------------------------------

                  Type: Numeric (long)

                 Range: [12001,59871]                Units: 1
         Unique values: 27                       Missing .: 3,453/9,273

                  Mean: 59387.8
             Std. dev.: 712.003

           Percentiles:     10%       25%       50%       75%       90%
                          59409     59409     59409     59409     59514

. tabulate AnesHCPCS if AnesHCPCS == ., missing

    CPT for |
    billing |
 anesthesia |
       care |      Freq.     Percent        Cum.
------------+-----------------------------------
          . |      3,457      100.00      100.00
------------+-----------------------------------
      Total |      3,457      100.00

. display %4.0f 100*(1-r(N)/_N) "% (" _N-r(N) "/" _N ")"
  89% (26703/30160)

.
. tabulate bAnesDeliv

 Anesthesia |
   care for |
  delivery, |
      0=No  |
 anesthesia |
  clinician |      Freq.     Percent        Cum.
------------+-----------------------------------
          0 |      9,273       30.75       30.75
          1 |     20,887       69.25      100.00
------------+-----------------------------------
      Total |     30,160      100.00
```

```
.
. tabulate Any_RBC_units // use manually for eighth paragraph of results, % units admission hemoglobin known or not

   0 or 1 = |
        RBC |      Freq.     Percent        Cum.
------------+-----------------------------------
          0 |     28,831       95.59       95.59
          1 |      1,329        4.41      100.00
------------+-----------------------------------
      Total |     30,160      100.00

. tabulate Any_RBC_Anes

   0 or 1 = |
RBC started |
     during |
 anesthesia |
       care |      Freq.     Percent        Cum.
------------+-----------------------------------
          0 |     29,875       99.06       99.06
          1 |        285        0.94      100.00
------------+-----------------------------------
      Total |     30,160      100.00

. tabulate Any_RBC_Anes if Blood_loss < 2500 // last sentence eighth paragraph Results

   0 or 1 = |
RBC started |
     during |
 anesthesia |
       care |      Freq.     Percent        Cum.
------------+-----------------------------------
          0 |     28,290       99.41       99.41
          1 |        168        0.59      100.00
------------+-----------------------------------
      Total |     28,458      100.00

.
. tabulate RBC_GE2

0 or 1 = 2+ |
  units RBC |      Freq.     Percent        Cum.
------------+-----------------------------------
          0 |     29,555       97.99       97.99
          1 |        605        2.01      100.00
------------+-----------------------------------
      Total |     30,160      100.00

. tabulate RBC_GE2_Anes

0 or 1 = 2+ |
  units RBC |
    started |
     during |
 anesthesia |
       care |      Freq.     Percent        Cum.
------------+-----------------------------------
          0 |     30,050       99.64       99.64
          1 |        110        0.36      100.00
------------+-----------------------------------
      Total |     30,160      100.00

.
. summarize N_units      if Any_RBC_units > 0, detail

                     RBC units transfused
-------------------------------------------------------------
      Percentiles      Smallest
 1%            1              1
 5%            1              1
10%            1              1       Obs               1,329
25%            1              1       Sum of wgt.       1,329

50%            1                      Mean           1.948834
                        Largest       Std. dev.      1.909969
75%            2             14
90%            3             17       Variance       3.647982
95%            5             25       Skewness       5.570068
99%           10             28       Kurtosis       54.01014

. summarize N_units_Anes if Any_RBC_Anes > 0, detail

          RBC units transfused during anesthesia care
-------------------------------------------------------------
      Percentiles      Smallest
 1%            1              1
 5%            1              1
10%            1              1       Obs                 285
25%            1              1       Sum of wgt.         285

50%            1                      Mean           1.621053
                        Largest       Std. dev.      1.265848
75%            2              7
90%            2              8       Variance       1.602372
95%            3              8       Skewness       4.954163
99%            8             14       Kurtosis       39.43678
```

```
.
. summarize QuanBloodLoss, detail

                Quantitative blood loss (ml)
-------------------------------------------------------------
      Percentiles      Smallest
 1%           45              2
 5%          100              3
10%          140              5       Obs              18,791
25%          242              5       Sum of wgt.      18,791

50%          443                      Mean           598.6977
                        Largest       Std. dev.      537.5862
75%          788           6836
90%         1247           7169       Variance       288998.9
95%         1615           8121       Skewness       2.894303
99%         2477           9755       Kurtosis       21.69786

. summarize Blood_loss   , detail

          Quantitative else estimated blood loss at
                        delivery, mL
-------------------------------------------------------------
      Percentiles      Smallest
 1%           50              1
 5%          104              1
10%          152              1       Obs              28,676
25%          250              1       Sum of wgt.      28,676

50%          400                      Mean           573.7696
                        Largest       Std. dev.       538.552
75%          798           9755
90%         1140          10000       Variance       290038.2
95%         1500          10000       Skewness       11.71203
99%         2331          35000       Kurtosis       604.7861

.
.
.
. *** Table S3 = Table 1 version B, patients with initial (admission) Hgb ******************************************
. use "Temp_Table S3.dta", clear

.
. describe, fullnames

Contains data from Temp_Table S3.dta
 Observations:        27,114
    Variables:            27                  17 Feb 2026 17:23
-----------------------------------------------------------------------------------------------------------------------------------
Variable      Storage   Display    Value
    name         type    format    label      Variable label
-----------------------------------------------------------------------------------------------------------------------------------
PAT_MRN_ID      long    %14.0g                Medical record number
Hosp_Adm        double  %tc                   Hospital admission date time
Hosp_DC         double  %tc                   Hospital discharge date time
Delivery        double  %tc                   Delivery date/ time
MaternalAge     byte    %10.0g                Maternal age (years)
Parity          byte    %10.0g                Parity
QuanBloodLoss   long    %12.0g                Quantitative blood loss (ml)
EstBloodLoss    long    %12.0g                Estimated blood loss (ml)
AnesHCPCS       long    %14.0f                CPT for billing anesthesia care
bAnesDeliv      int     %10.0f                Anesthesia care for delivery, 0=No anesthesia clinician
Hgb_Adm         double  %10.1f                Hemoglobin g/dl
AnesStartBeforeAtHgb
                double  %tc                   Case Start Datetime, but only if before/at time of Hgb check
AnesEndPlus2Hr  double  %tc                   Case Stop Datetime + 2 hr; Dexter F 18 page 1655 last paragraph
HrsDifference   double  %10.0g                Hgb Time checked in hours after (+) or before(-) admission
RBCtime         double  %tc                   Date/Time start RBC transfusion
bAnesTrans      int     %10.0f                Anesthesia care when transfusion started
N_units         int     %8.0g                 RBC units transfused among patients with Hgb measured
N_units_Anes    int     %8.0g                 RBC units transfused during anesthesia among patients with Hgb measured
Any_RBC_units   byte    %8.0g                 0 or 1 = RBC
Any_RBC_Anes    byte    %8.0g                 0 or 1 = RBC started during anesthesia care
RBC_GE2         byte    %8.0g                 0 or 1 = 2+ units RBC
RBC_GE2_Anes    byte    %8.0g                 0 or 1 = 2+ units RBC started during anesthesia care
RBCtimeHr       double  %10.0g                RBC first unit, if any, transfused relative to time of delivery
Hgb_Decile      byte    %8.0g                 10 quantiles of Hgb_Adm
MedianHgb       double  %10.0g                Median Hgb g/dl of decile
Weight_kg       double  %6.1f                 Weight (kg) most recent before delivery
AllowBloodLoss  double  %10.0g                Allowable Blood Loss (mL) - Russo Formula
-----------------------------------------------------------------------------------------------------------------------------------
Sorted by: PAT_MRN_ID  Hosp_Adm

.
. generate long  Blood_loss = .
(27,114 missing values generated)

. replace        Blood_loss = QuanBloodLoss if QuanBloodLoss != .
(18,167 real changes made)

. replace        Blood_loss = EstBloodLoss  if QuanBloodLoss == . & EstBloodLoss != .
(7,723 real changes made)

. label variable Blood_loss "Quantitative else estimated blood loss at delivery, mL"

.
```

```
. summarize MaternalAge, detail

                     Maternal age (years)
-------------------------------------------------------------
      Percentiles      Smallest
 1%           18             13
 5%           21             14
10%           22             14       Obs              27,114
25%           26             14       Sum of wgt.      27,114

50%           30                      Mean           29.73604
                        Largest       Std. dev.       5.45535
75%           33             51
90%           37             52       Variance       29.76085
95%           39             52       Skewness       .0445427
99%           42             57       Kurtosis       2.802017

. summarize Parity     , detail

                            Parity
-------------------------------------------------------------
      Percentiles      Smallest
 1%            1              1
 5%            1              1
10%            1              1       Obs              27,048
25%            1              1       Sum of wgt.      27,048

50%            2                      Mean           2.120859
                        Largest       Std. dev.      1.278094
75%            3             13
90%            4             13       Variance       1.633524
95%            5             13       Skewness       1.686806
99%            6             14       Kurtosis       7.736836

.
. generate bCesarean = 0

. replace  bCesarean = 1 if AnesHCPCS == 59510 | AnesHCPCS == 59514 | AnesHCPCS == 59515 | ///
>                           AnesHCPCS == 59525 | AnesHCPCS == 59620 | AnesHCPCS == 59622
(10,155 real changes made)

. tabulate bCesarean

  bCesarean |      Freq.     Percent        Cum.
------------+-----------------------------------
          0 |     16,959       62.55       62.55
          1 |     10,155       37.45      100.00
------------+-----------------------------------
      Total |     27,114      100.00

.
. generate double HoursBeforeDelivery = (Delivery - Hosp_Adm)/ (1000*60*60)

. summarize       HoursBeforeDelivery, detail

                     HoursBeforeDelivery
-------------------------------------------------------------
      Percentiles      Smallest
 1%     1.033611       2.78e-07
 5%     2.283333       2.78e-07
10%     2.833333       2.78e-07       Obs              27,114
25%     5.283333       2.78e-07       Sum of wgt.      27,114

50%     11.68361                      Mean           30.42295
                        Largest       Std. dev.      98.22261
75%     21.91694       1883.367
90%     42.68361       1893.934       Variance       9647.681
95%     77.33333           2023       Skewness       9.252552
99%     501.8336       2115.879       Kurtosis       112.6652

.
. generate double HoursAfterDelivery  = (Hosp_DC - Delivery) / (1000*60*60)

. summarize       HoursAfterDelivery, detail

                     HoursAfterDelivery
-------------------------------------------------------------
      Percentiles      Smallest
 1%     4.416389            1.1
 5%         25.9       1.133333
10%        31.15       1.233056       Obs              27,114
25%        40.65       1.241667       Sum of wgt.      27,114

50%     50.48333                      Mean           54.25223
                        Largest       Std. dev.       26.6981
75%     66.26639       806.9153
90%        77.15       894.2833       Variance       712.7887
95%        84.15         1010.3       Skewness       11.16553
99%     128.9664       1345.833       Kurtosis       370.1939

.
. codebook AnesHCPCS if bAnesDeliv == 1 & AnesHCPCS == .

-------------------------------------------------------------------------------------------------
AnesHCPCS                                                         CPT for billing anesthesia care
-------------------------------------------------------------------------------------------------

                  Type: Numeric (long)

                 Range: [.,.]                         Units: .
         Unique values: 0                         Missing .: 4/4

            Tabulation: Freq.  Value
                            4  .
```

```
. codebook AnesHCPCS if bAnesDeliv == 0

-------------------------------------------------------------------------------
AnesHCPCS                                      CPT for billing anesthesia care
-------------------------------------------------------------------------------

                  Type: Numeric (long)

                 Range: [12001,59871]                Units: 1
         Unique values: 27                       Missing .: 2,316/7,270

                  Mean:   59387
             Std. dev.: 765.564

           Percentiles:     10%       25%       50%       75%       90%
                          59409     59409     59409     59409     59514

. tabulate AnesHCPCS if AnesHCPCS == ., missing

    CPT for |
    billing |
 anesthesia |
       care |      Freq.     Percent        Cum.
------------+-----------------------------------
          . |      2,320      100.00      100.00
------------+-----------------------------------
      Total |      2,320      100.00

. display %4.0f 100*(1-r(N)/_N) "% (" _N-r(N) "/" _N ")"
  91% (24794/27114)

.
. tabulate bAnesDeliv

 Anesthesia |
   care for |
  delivery, |
       0=No |
 anesthesia |
  clinician |      Freq.     Percent        Cum.
------------+-----------------------------------
          0 |      7,270       26.81       26.81
          1 |     19,844       73.19      100.00
------------+-----------------------------------
      Total |     27,114      100.00

.
. tabulate Any_RBC_units // used manually for eighth paragraph of results, % units admission hemoglobin known or not

   0 or 1 = |
        RBC |      Freq.     Percent        Cum.
------------+-----------------------------------
          0 |     25,835       95.28       95.28
          1 |      1,279        4.72      100.00
------------+-----------------------------------
      Total |     27,114      100.00

. tabulate Any_RBC_Anes

   0 or 1 = |
RBC started |
     during |
 anesthesia |
       care |      Freq.     Percent        Cum.
------------+-----------------------------------
          0 |     26,835       98.97       98.97
          1 |        279        1.03      100.00
------------+-----------------------------------
      Total |     27,114      100.00

. tabulate Any_RBC_Anes if Blood_loss < 2500 // last sentence eighth paragraph Results

   0 or 1 = |
RBC started |
     during |
 anesthesia |
       care |      Freq.     Percent        Cum.
------------+-----------------------------------
          0 |     25,512       99.35       99.35
          1 |        166        0.65      100.00
------------+-----------------------------------
      Total |     25,678      100.00

.
. tabulate RBC_GE2

0 or 1 = 2+ |
  units RBC |      Freq.     Percent        Cum.
------------+-----------------------------------
          0 |     26,538       97.88       97.88
          1 |        576        2.12      100.00
------------+-----------------------------------
      Total |     27,114      100.00

. tabulate RBC_GE2_Anes

0 or 1 = 2+ |
  units RBC |
    started |
     during |
 anesthesia |
       care |      Freq.     Percent        Cum.
------------+-----------------------------------
          0 |     27,007       99.61       99.61
          1 |        107        0.39      100.00
------------+-----------------------------------
      Total |     27,114      100.00
```

```
.
. summarize N_units      if Any_RBC_units > 0, detail

        RBC units transfused among patients with Hgb
                          measured
-------------------------------------------------------------
      Percentiles      Smallest
 1%            1              1
 5%            1              1
10%            1              1       Obs               1,279
25%            1              1       Sum of wgt.       1,279

50%            1                      Mean           1.949179
                        Largest       Std. dev.      1.930664
75%            2             14
90%            3             17       Variance       3.727462
95%            5             25       Skewness       5.574967
99%           10             28       Kurtosis       53.66831

. summarize N_units_Anes if Any_RBC_Anes  > 0, detail

        RBC units transfused during anesthesia among
                 patients with Hgb measured
-------------------------------------------------------------
      Percentiles      Smallest
 1%            1              1
 5%            1              1
10%            1              1       Obs                 279
25%            1              1       Sum of wgt.         279

50%            1                      Mean           1.623656
                        Largest       Std. dev.      1.277199
75%            2              7
90%            2              8       Variance       1.631237
95%            4              8       Skewness       4.922371
99%            8             14       Kurtosis       38.83678

.
. summarize QuanBloodLoss , detail

                Quantitative blood loss (ml)
-------------------------------------------------------------
      Percentiles      Smallest
 1%           48              2
 5%          100              3
10%          141              5       Obs              18,167
25%          244              5       Sum of wgt.      18,167

50%          449                      Mean           603.5034
                        Largest       Std. dev.      540.4414
75%          794           6836
90%         1258           7169       Variance       292076.9
95%         1620           8121       Skewness       2.898869
99%         2484           9755       Kurtosis       21.76798

. summarize Blood_loss    , detail

          Quantitative else estimated blood loss at
                        delivery, mL
-------------------------------------------------------------
      Percentiles      Smallest
 1%           50              1
 5%          104              1
10%          152              1       Obs              25,890
25%          250              1       Sum of wgt.      25,890

50%          427                      Mean           588.6325
                        Largest       Std. dev.      553.2194
75%          800           9755
90%       1175.5          10000       Variance       306051.7
95%         1500          10000       Skewness       11.85456
99%         2381          35000       Kurtosis       600.1656

. summarize AllowBloodLoss, detail

          Allowable Blood Loss (mL) - Russo Formula
-------------------------------------------------------------
      Percentiles      Smallest
 1%     1249.773              0
 5%     1789.798              0
10%       1997.5              0       Obs              26,588
25%      2291.25              0       Sum of wgt.      26,588

50%     2545.833                      Mean           2488.881
                        Largest       Std. dev.      395.6515
75%     2768.594       3651.954
90%     2941.852       3720.615       Variance       156540.1
95%     3033.022       3733.889       Skewness      -1.034129
99%     3200.476       4217.522       Kurtosis       5.485761

.
. summarize Hgb_Adm      , detail

                       Hemoglobin g/dl
-------------------------------------------------------------
      Percentiles      Smallest
 1%          8.8            4.1
 5%          9.9            5.5
10%         10.4            6.1       Obs              27,114
25%         11.2            6.5       Sum of wgt.      27,114

50%           12                      Mean           11.97541
                        Largest       Std. dev.      1.230448
75%         12.8           17.4
90%         13.5           17.9       Variance       1.514003
95%         13.9             18       Skewness        -.18385
99%         14.7           22.6       Kurtosis        3.66535
```

```
.
. generate byte bHgb_Adm_GT_9 = .
(27,114 missing values generated)

. replace       bHgb_Adm_GT_9 = 1 if Hgb_Adm > 9 & Hgb_Adm != .
(26,753 real changes made)

. replace       bHgb_Adm_GT_9 = 0 if Hgb_Adm <=9 & Hgb_Adm != .
(361 real changes made)

. tabulate      bHgb_Adm_GT_9 // Table S3 (1B), footnote d last sentence; 2nd to last paragraph discussion

bHgb_Adm_GT |
         _9 |      Freq.     Percent        Cum.
------------+-----------------------------------
          0 |        361        1.33        1.33
          1 |     26,753       98.67      100.00
------------+-----------------------------------
      Total |     27,114      100.00

.
. generate byte  bAnemic = 0

. replace        bAnemic = 1 if Hgb_Adm < 11
(5,203 real changes made)

. label variable bAnemic  "1 if Hgb_Adm < 11 g/dL, 0 otherwise"

. tabulate       bAnemic

      1 if |
 Hgb_Adm < |
11 g/dL, 0 |
 otherwise |      Freq.     Percent        Cum.
------------+-----------------------------------
          0 |     21,911       80.81       80.81
          1 |      5,203       19.19      100.00
------------+-----------------------------------
      Total |     27,114      100.00

.
. by bAnemic, sort : summarize Blood_loss // Table S3, footnote c; anemic patients had greater blood loss

-------------------------------------------------------------------------------------------------------------------------------------
-> bAnemic = 0

    Variable |        Obs        Mean    Std. dev.       Min        Max
-------------+---------------------------------------------------------
  Blood_loss |     20,925    576.8995    546.2562          1      35000

-------------------------------------------------------------------------------------------------------------------------------------
-> bAnemic = 1

    Variable |        Obs        Mean    Std. dev.       Min        Max
-------------+---------------------------------------------------------
  Blood_loss |      4,965    638.0812    579.1012          1      10000


. ranksum Blood_loss, by(bAnemic) porder

Two-sample Wilcoxon rank-sum (Mann-Whitney) test

     bAnemic |      Obs    Rank sum    Expected
-------------+---------------------------------
           0 |    20925   2.681e+08   2.709e+08
           1 |     4965    67081154    64274408
-------------+---------------------------------
    Combined |    25890   3.352e+08   3.352e+08

Unadjusted variance   2.242e+11
Adjustment for ties   -57602593
                     ----------
Adjusted variance     2.241e+11

H0: Blood_~s(bAnemic==0) = Blood_~s(bAnemic==1)
         z = -5.929
Prob > |z| = 0.0000

P{Blood_~s(bAnemic==0) > Blood_~s(bAnemic==1)} = 0.473

.
.
.
. *** Table S4 = Table 1 version C, patients with initial (admission) and final (discharge) Hgb ********************
. use "Temp_Table S4.dta", clear

.
. describe, fullnames

Contains data from Temp_Table S4.dta
 Observations:         4,817
    Variables:            26                  17 Feb 2026 17:23
-------------------------------------------------------------------------------------------------------------------------------------
Variable      Storage   Display    Value
    name         type    format    label      Variable label
-------------------------------------------------------------------------------------------------------------------------------------
PAT_MRN_ID      long    %14.0g                Medical record number
Hosp_Adm        double  %tc                   Hospital admission date time
Hosp_DC         double  %tc                   Hospital discharge date time
Delivery        double  %tc                   Delivery date/ time
MaternalAge     byte    %10.0g                Maternal age (years)
Parity          byte    %10.0g                Parity
QuanBloodLoss   long    %12.0g                Quantitative blood loss (ml)
EstBloodLoss    long    %12.0g                Estimated blood loss (ml)
AnesHCPCS       long    %14.0f                CPT for billing anesthesia care
bAnesDeliv      int     %10.0f                Anesthesia care for delivery, 0=No anesthesia clinician
Hgb_DC          double  %10.1f                Hemoglobin g/dl
AnesStartBeforeAtHgb
```

```
                  double  %tc                       Case Start Datetime, but only if before/at time of Hgb check
AnesEndPlus2Hr    double  %tc                       Case Stop Datetime + 2 hr; Dexter F 18 page 1655 last paragraph
Hgb_Adm           double  %10.1f                    Hemoglobin g/dl
HrsDifference     double  %10.0g
N_units           int     %8.0g                     RBC units transfused among patients with Hgb measured
N_units_Anes      int     %8.0g                     RBC units transfused during anesthesia care among patients with Hgb measured
Blood_loss        long    %12.0g                    Quantitative else estimated blood loss at delivery, mL
Any_RBC_units     byte    %8.0g                     0 or 1 = RBC
Any_RBC_Anes      byte    %8.0g                     0 or 1 = RBC started during anesthesia care
RBC_GE2           byte    %8.0g                     0 or 1 = 2+ units RBC
RBC_GE2_Anes      byte    %8.0g                     0 or 1 = 2+ units RBC started during anesthesia care
Hgb_Decile_DC     byte    %8.0g                     10 quantiles of Hgb_DC
MedianHgb         double  %10.0g                    Median Hgb g/dl at discharge of decile
Weight_kg         double  %6.1f                     Weight (kg) most recent before delivery
AllowBloodLoss    double  %10.0g                    Allowable Blood Loss (mL) - Russo Formula
--------------------------------------------------------------------------------------------------------------------------------
Sorted by: PAT_MRN_ID  Hosp_Adm

.
. summarize MaternalAge, detail

                     Maternal age (years)
-------------------------------------------------------------
      Percentiles      Smallest
 1%           18             14
 5%           21             14
10%           23             14       Obs               4,817
25%           26             15       Sum of wgt.       4,817

50%           30                      Mean           30.33714
                        Largest       Std. dev.       5.72023
75%           34             49
90%           38             50       Variance       32.72103
95%           40             51       Skewness        .018266
99%           43             52       Kurtosis       2.684325

. summarize Parity     , detail

                            Parity
-------------------------------------------------------------
      Percentiles      Smallest
 1%            1              1
 5%            1              1
10%            1              1       Obs               4,810
25%            1              1       Sum of wgt.       4,810

50%            2                      Mean           2.086279
                        Largest       Std. dev.      1.363548
75%            3             11
90%            4             12       Variance       1.859263
95%            5             13       Skewness       1.889475
99%            7             13       Kurtosis        8.51548

.
. generate bCesarean = 0

. replace  bCesarean = 1 if AnesHCPCS == 59510 | AnesHCPCS == 59514 | AnesHCPCS == 59515 | ///
>                           AnesHCPCS == 59525 | AnesHCPCS == 59620 | AnesHCPCS == 59622
(2,934 real changes made)

. tabulate bCesarean

  bCesarean |      Freq.     Percent        Cum.
------------+-----------------------------------
          0 |      1,883       39.09       39.09
          1 |      2,934       60.91      100.00
------------+-----------------------------------
      Total |      4,817      100.00

.
. generate double HoursBeforeDelivery = (Delivery - Hosp_Adm)/ (1000*60*60)

. summarize       HoursBeforeDelivery, detail

                      HoursBeforeDelivery
-------------------------------------------------------------
      Percentiles      Smallest
 1%          .85       2.78e-07
 5%     2.386944       2.78e-07
10%     2.866944       2.78e-07       Obs               4,817
25%     5.546111       2.78e-07       Sum of wgt.       4,817

50%     16.32917                      Mean           47.55729
                        Largest       Std. dev.      132.3472
75%         33.8       1584.867
90%     76.51667        1652.65       Variance       17515.79
95%     163.9669       1858.749       Skewness       6.890344
99%     733.3117       2115.879       Kurtosis       63.99195

.
. generate double HoursAfterDelivery  = (Hosp_DC - Delivery) / (1000*60*60)

. summarize       HoursAfterDelivery, detail

                      HoursAfterDelivery
-------------------------------------------------------------
      Percentiles      Smallest
 1%     21.98333       1.241667
 5%     34.54972       1.454722
10%     41.86111       3.199722       Obs               4,817
25%     51.43333       3.666389       Sum of wgt.       4,817
```

```
50%     66.95444                      Mean           69.14721
                        Largest       Std. dev.      36.59193
75%     78.03333       588.4333
90%        93.55       606.6167       Variance        1338.97
95%     112.7333       806.9153       Skewness       8.906022
99%     184.1164         1010.3       Kurtosis       164.8612

.
. codebook AnesHCPCS if bAnesDeliv == 1 & AnesHCPCS == .

-------------------------------------------------------------------------------------------
AnesHCPCS                                                   CPT for billing anesthesia care
-------------------------------------------------------------------------------------------

                  Type: Numeric (long)

                 Range: [.,.]                        Units: .
         Unique values: 0                        Missing .: 1/1

           Tabulation: Freq.  Value
                           1  .

. codebook AnesHCPCS if bAnesDeliv == 0

-------------------------------------------------------------------------------------------
AnesHCPCS                                                   CPT for billing anesthesia care
-------------------------------------------------------------------------------------------

                  Type: Numeric (long)

                 Range: [37244,59871]                Units: 1
         Unique values: 21                       Missing .: 366/907

                  Mean: 59332.1
             Std. dev.: 1007.32

           Percentiles:     10%       25%       50%       75%       90%
                          59409     59409     59409     59409     59514

. tabulate AnesHCPCS if AnesHCPCS == ., missing

    CPT for |
    billing |
 anesthesia |
       care |      Freq.     Percent        Cum.
------------+-----------------------------------
          . |        367      100.00      100.00
------------+-----------------------------------
      Total |        367      100.00

. display %4.0f 100*(1-r(N)/_N) "% (" _N-r(N) "/" _N ")"
  92% (4450/4817)

.
. tabulate bAnesDeliv

 Anesthesia |
   care for |
  delivery, |
       0=No |
 anesthesia |
  clinician |      Freq.     Percent        Cum.
------------+-----------------------------------
          0 |        907       18.83       18.83
          1 |      3,910       81.17      100.00
------------+-----------------------------------
      Total |      4,817      100.00

.
. tabulate Any_RBC_units

     0 or 1 = |
          RBC |      Freq.     Percent        Cum.
------------+-----------------------------------
          0 |      3,809       79.07       79.07
          1 |      1,008       20.93      100.00
------------+-----------------------------------
      Total |      4,817      100.00

. tabulate Any_RBC_Anes

     0 or 1 = |
  RBC started |
       during |
   anesthesia |
         care |      Freq.     Percent        Cum.
------------+-----------------------------------
          0 |      4,560       94.66       94.66
          1 |        257        5.34      100.00
------------+-----------------------------------
      Total |      4,817      100.00

.
. tabulate RBC_GE2

0 or 1 = 2+ |
  units RBC |      Freq.     Percent        Cum.
------------+-----------------------------------
          0 |      4,327       89.83       89.83
          1 |        490       10.17      100.00
------------+-----------------------------------
      Total |      4,817      100.00
```

```
. tabulate RBC_GE2_Anes

0 or 1 = 2+ |
  units RBC |
    started |
     during |
 anesthesia |
       care |      Freq.     Percent        Cum.
------------+-----------------------------------
          0 |      4,716       97.90       97.90
          1 |        101        2.10      100.00
------------+-----------------------------------
      Total |      4,817      100.00

.
. summarize N_units      if Any_RBC_units > 0, detail

        RBC units transfused among patients with Hgb
                          measured
-------------------------------------------------------------
      Percentiles      Smallest
 1%            1              1
 5%            1              1
10%            1              1       Obs               1,008
25%            1              1       Sum of wgt.       1,008

50%            1                      Mean           2.048611
                        Largest       Std. dev.      1.967617
75%            2             14
90%            4             14       Variance       3.871517
95%            5             17       Skewness       4.865456
99%           11             28       Kurtosis       43.16743

. summarize N_units_Anes if Any_RBC_Anes > 0, detail

      RBC units transfused during anesthesia care among
                 patients with Hgb measured
-------------------------------------------------------------
      Percentiles      Smallest
 1%            1              1
 5%            1              1
10%            1              1       Obs                 257
25%            1              1       Sum of wgt.         257

50%            1                      Mean           1.649805
                        Largest       Std. dev.      1.317672
75%            2              7
90%            3              8       Variance        1.73626
95%            4              8       Skewness       4.810613
99%            8             14       Kurtosis       36.84508

.
. summarize QuanBloodLoss , detail

                Quantitative blood loss (ml)
-------------------------------------------------------------
      Percentiles      Smallest
 1%           92             12
 5%          214             19
10%          334             30       Obs               3,857
25%          630             32       Sum of wgt.       3,857

50%         1077                      Mean           1165.702
                        Largest       Std. dev.      768.7074
75%         1534           6836
90%         2035           7169       Variance         590911
95%         2431           8121       Skewness       2.106038
99%         3672           9755       Kurtosis       14.42219

. summarize Blood_loss    , detail

          Quantitative else estimated blood loss at
                       delivery, mL
-------------------------------------------------------------
      Percentiles      Smallest
 1%           92              5
 5%          200              5
10%          311             12       Obs               4,647
25%          600             19       Sum of wgt.       4,647

50%         1024                      Mean           1142.292
                        Largest       Std. dev.      923.8397
75%         1500           9755
90%         2000          10000       Variance       853479.8
95%         2419          10000       Skewness       12.09847
99%         3615          35000       Kurtosis        398.322

. summarize AllowBloodLoss, detail

          Allowable Blood Loss (mL) - Russo Formula
-------------------------------------------------------------
      Percentiles      Smallest
 1%       763.75              0
 5%         1560              0
10%         1833              0       Obs               4,680
25%     2186.147              0       Sum of wgt.       4,680

50%     2485.424                      Mean           2408.515
                        Largest       Std. dev.       460.239
75%     2715.556       3517.879
90%     2894.211        3608.83       Variance       211819.9
95%     2988.102       3733.889       Skewness      -1.266946
99%     3180.548       4217.522       Kurtosis       5.942245
```

```
.
. summarize Hgb_Adm, detail

                       Hemoglobin g/dl
-------------------------------------------------------------
      Percentiles      Smallest
 1%            8            5.5
 5%          9.4            6.9
10%           10            6.9       Obs               4,817
25%         10.9            6.9       Sum of wgt.       4,817

50%         11.8                      Mean           11.73631
                        Largest       Std. dev.      1.345342
75%         12.6           17.1
90%         13.3           17.1       Variance       1.809946
95%         13.8             18       Skewness      -.2028406
99%         14.6           22.6       Kurtosis       4.483862

.
. generate byte bAnemic_Adm = 0

. replace       bAnemic_Adm = 1 if Hgb_Adm < 11
(1,225 real changes made)

. tabulate bAnemic_Adm

bAnemic_Adm |      Freq.     Percent        Cum.
------------+-----------------------------------
          0 |      3,592       74.57       74.57
          1 |      1,225       25.43      100.00
------------+-----------------------------------
      Total |      4,817      100.00

.
. summarize Hgb_DC , detail

                       Hemoglobin g/dl
-------------------------------------------------------------
      Percentiles      Smallest
 1%          6.9            4.2
 5%          7.8            4.4
10%          8.4            4.5       Obs               4,817
25%          9.3            5.1       Sum of wgt.       4,817

50%         10.3                      Mean           10.28453
                        Largest       Std. dev.       1.47513
75%         11.3           15.1
90%         12.1           15.2       Variance       2.176009
95%         12.7           15.8       Skewness       .0141038
99%         13.6           20.6       Kurtosis       3.492554

.
. generate byte bAnemic_DC = 0

. replace       bAnemic_DC = 1 if Hgb_DC <9
(862 real changes made)

. tabulate bAnemic_DC

 bAnemic_DC |      Freq.     Percent        Cum.
------------+-----------------------------------
          0 |      3,955       82.11       82.11
          1 |        862       17.89      100.00
------------+-----------------------------------
      Total |      4,817      100.00

.
. generate byte bAnemic_Severe_DC = 0

. replace       bAnemic_Severe_DC = 1 if Hgb_DC < 8
(286 real changes made)

. tabulate      bAnemic_Severe_DC

bAnemic_Sev |
     ere_DC |      Freq.     Percent        Cum.
------------+-----------------------------------
          0 |      4,531       94.06       94.06
          1 |        286        5.94      100.00
------------+-----------------------------------
      Total |      4,817      100.00

.
. cs bAnemic_DC bAnemic_Adm, or exact level(99)  // Aghajanian S et al. 2024 prediction, Table S4 footnote d

                 |      bAnemic_Adm        |
                 |   Exposed   Unexposed   |      Total
-----------------+-------------------------+-----------
           Cases |       508         354   |        862
        Noncases |       717        3238   |       3955
-----------------+-------------------------+-----------
           Total |      1225        3592   |       4817
                 |                         |
            Risk |  .4146939    .0985523   |   .1789496
                 |                         |
                 |      Point estimate     |    [99% conf. interval]
                 |-------------------------+-----------------------
 Risk difference |         .3161415        |   .2776871     .354596
      Risk ratio |         4.207854        |   3.597719    4.921462
 Attr. frac. ex. |         .7623492        |   .7220461    .7968083
 Attr. frac. pop |         .4492731        |
      Odds ratio |         6.480644        |   5.266452    7.974781 (Cornfield)
                 +-------------------------------------------------
                               1-sided Fisher's exact P = 0.0000
                               2-sided Fisher's exact P = 0.0000
```

```
.
.
.
. *** First paragraph of Results, using same file as for Table 1 ***************************************************
. use "Temp_Table 1.dta", clear

.
. describe, fullnames

Contains data from Temp_Table 1.dta
 Observations:        30,160
    Variables:            19                  17 Feb 2026 17:23
------------------------------------------------------------------------------------------------------------------------------------
Variable      Storage   Display    Value
    name         type    format    label      Variable label
------------------------------------------------------------------------------------------------------------------------------------
PAT_MRN_ID      long    %14.0g                Medical record number
Hosp_Adm        double  %tc                   Hospital admission date time
Hosp_DC         double  %tc                   Hospital discharge date time
Delivery        double  %tc                   Delivery date/ time
MaternalAge     byte    %10.0g                Maternal age (years)
Parity          byte    %10.0g                Parity
QuanBloodLoss   long    %12.0g                Quantitative blood loss (ml)
EstBloodLoss    long    %12.0g                Estimated blood loss (ml)
AnesHCPCS       long    %14.0f                CPT for billing anesthesia care
bAnesDeliv      int     %10.0f                Anesthesia care for delivery, 0=No anesthesia clinician
RBCtime         double  %tc                   Date/Time start RBC transfusion
bAnesTrans      int     %10.0f                Anesthesia care when transfusion started
N_units         int     %8.0g                 RBC units transfused
N_units_Anes    int     %8.0g                 RBC units transfused during anesthesia care
Any_RBC_units   byte    %8.0g                 0 or 1 = RBC
Any_RBC_Anes    byte    %8.0g                 0 or 1 = RBC started during anesthesia care
RBC_GE2         byte    %8.0g                 0 or 1 = 2+ units RBC
RBC_GE2_Anes    byte    %8.0g                 0 or 1 = 2+ units RBC started during anesthesia care
Blood_loss      long    %12.0g                Quantitative else estimated blood loss at delivery, mL
------------------------------------------------------------------------------------------------------------------------------------
Sorted by: PAT_MRN_ID  Hosp_Adm  RBCtime

.
. keep PAT_MRN_ID Delivery

. sort PAT_MRN_ID Delivery

. bysort PAT_MRN_ID: egen first_delivery = min(Delivery)

. bysort PAT_MRN_ID: egen last_delivery  = max(Delivery)

. generate double delivery_diff_years = (last_delivery - first_delivery)/ (1000 * 60 * 60 * 24 * 365)

. replace         delivery_diff_years = . if PAT_MRN_ID == PAT_MRN_ID[_n-1]
(7,021 real changes made, 7,021 to missing)

. format %4.1f    delivery_diff_years

.
. * codebook       PAT_MRN_ID                // for sentence in Results
. quietly levelsof PAT_MRN_ID, local(levels) // same, but without displaying sample observations

. local ndistinct : word count `levels'

. display "Distinct values in PAT_MRN_ID: `ndistinct'"
Distinct values in PAT_MRN_ID: 23139

.
. summarize       delivery_diff_years if delivery_diff_years > 0, detail

                     delivery_diff_years
-------------------------------------------------------------
      Percentiles      Smallest
 1%     .9384391       .5701067
 5%     1.269394       .6535686
10%     1.539219        .676266       Obs               5,605
25%     2.042493       .7435726       Sum of wgt.       5,605

50%     2.949191                      Mean           3.574287
                        Largest       Std. dev.      2.063972
75%     4.617778       11.69539
90%     6.538151       11.91559       Variance       4.259982
95%     7.847146       12.00146       Skewness        1.23874
99%     10.19493       12.00914       Kurtosis       4.266459

.
.
.
. *** Second paragraph of the Results ******************************************************************************
. use "Temp_Table 1.dta", clear

.
. ratio (N_units_Anes/N_units) if N_units > 0, level(99) // also seventh paragraph Results

Ratio estimation                        Number of obs = 1,329

     _ratio_1: N_units_Anes/N_units

--------------------------------------------------------------
             |             Linearized
             |      Ratio   std. err.     [99% conf. interval]
-------------+------------------------------------------------
    _ratio_1 |   .1783784    .0118269      .1478704    .2088863
--------------------------------------------------------------
```

```
. display "Among all peripartum RBC units, " %4.1f 100*r(table)[1,1] "% are transfused during anesthesia care."
Among all peripartum RBC units, 17.8% are transfused during anesthesia care.

. summarize N_units_Anes       if N_units > 0

    Variable |        Obs        Mean    Std. dev.       Min        Max
-------------+---------------------------------------------------------
N_units_Anes |      1,329    .3476298     .886391          0         14

. display "Total for numerator of fraction = "   r(N)*r(mean)
Total for numerator of fraction = 462

. summarize N_units            if N_units > 0

    Variable |        Obs        Mean    Std. dev.       Min        Max
-------------+---------------------------------------------------------
     N_units |      1,329    1.948834    1.909969          1         28

. display "Total for denominator of fraction = " r(N)*r(mean)
Total for denominator of fraction = 2590

.
. clonevar Any_RBC_Not_Anes = Any_RBC_units

. assert   N_units - N_units_Anes >= 0

. replace  Any_RBC_Not_Anes = 0 if N_units - N_units_Anes == 0
(148 real changes made)

. tabulate Any_RBC_Anes Any_RBC_Not_Anes

  0 or 1 = |
       RBC |
   started |
    during |
anesthesia |     0 or 1 = RBC
      care |         0          1 |     Total
-----------+----------------------+----------
         0 |    28,831      1,044 |    29,875 
         1 |       148        137 |       285 
-----------+----------------------+----------
     Total |    28,979      1,181 |    30,160 

. mcc      Any_RBC_Anes Any_RBC_Not_Anes, level(99) // RBC during anesthesia care reduces probability subsequent transfusion

                 |        Controls        |
Cases            |   Exposed   Unexposed  |      Total
-----------------+------------------------+-----------
         Exposed |       137         148  |        285
       Unexposed |      1044       28831  |      29875
-----------------+------------------------+-----------
           Total |      1181       28979  |      30160

McNemar's chi2(1) =    673.50    Prob > chi2 = 0.0000
Exact McNemar significance probability       = 0.0000

Proportion with factor
        Cases       .0094496
        Controls    .0391578     [99% conf. interval]
                   ---------     --------------------
        difference -.0297082     -.0326569  -.0267595
        ratio       .2413209       .207025   .2812983
        rel. diff. -.0309189     -.0340348   -.027803

        odds ratio  .1417625      .1119584   .1774396   (exact)

. display %5.4f 3*r(p_exact)
0.0000

.
. clonevar RBC_1_Anes = Any_RBC_Anes

. replace  RBC_1_Anes = 0 if RBC_GE2_Anes == 1
(110 real changes made)

. tabulate RBC_1_Anes Any_RBC_Not_Anes

  0 or 1 = |
       RBC |
   started |
    during |
anesthesia |     0 or 1 = RBC
      care |         0          1 |     Total
-----------+----------------------+----------
         0 |    28,884      1,101 |    29,985 
         1 |        95         80 |       175 
-----------+----------------------+----------
     Total |    28,979      1,181 |    30,160 

. mcc      RBC_1_Anes Any_RBC_Not_Anes, level(99) // applies to one unit by anesthesia

                 |        Controls        |
Cases            |   Exposed   Unexposed  |      Total
-----------------+------------------------+-----------
         Exposed |        80          95  |        175
       Unexposed |      1101       28884  |      29985
-----------------+------------------------+-----------
           Total |      1181       28979  |      30160

McNemar's chi2(1) =    846.18    Prob > chi2 = 0.0000
Exact McNemar significance probability       = 0.0000
```

```
Proportion with factor
        Cases       .0058024
        Controls    .0391578     [99% conf. interval]
                    ---------    --------------------
        difference -.0333554     -.0363005  -.0304104
        ratio       .1481795      .1218118   .1802548
        rel. diff. -.0347148     -.0378417  -.0315879

        odds ratio  .0862852      .0644907   .1132283   (exact)

. display %5.4f 3*r(p_exact)
0.0000

.
. clonevar RBC_GE2_Not_Anes = RBC_GE2

. replace  RBC_GE2_Not_Anes = 0 if N_units - N_units_Anes < 2
(129 real changes made)

. tabulate RBC_GE2_Anes RBC_GE2_Not_Anes

  0 or 1 = |
  2+ units |
       RBC |
   started |
    during |
anesthesia | 0 or 1 = 2+ units RBC
      care |         0          1 |     Total
-----------+----------------------+----------
         0 |    29,604        446 |    30,050
         1 |        80         30 |       110
-----------+----------------------+----------
     Total |    29,684        476 |    30,160

. mcc      RBC_GE2_Anes RBC_GE2_Not_Anes, level(99) // and to two or more units

                 |        Controls        |
Cases            |   Exposed   Unexposed  |      Total
-----------------+------------------------+-----------
         Exposed |        30          80  |        110
       Unexposed |       446       29604  |      30050
-----------------+------------------------+-----------
           Total |       476       29684  |      30160

McNemar's chi2(1) =     254.67    Prob > chi2 = 0.0000
Exact McNemar significance probability       = 0.0000

Proportion with factor
        Cases       .0036472
        Controls    .0157825     [99% conf. interval]
                    ---------    --------------------
        difference -.0121353     -.0141189  -.0101517
        ratio       .2310924      .1785101   .2991635
        rel. diff. -.0123299     -.0143323  -.0103275

        odds ratio  .1793722       .128829   .2445862   (exact)

. display %5.4f 3*r(p_exact)
0.0000

.
.
.
. *** Third paragraph of the Results ********************************************************************************
. use "Temp_Table S3.dta", clear

.
. describe, fullnames

Contains data from Temp_Table S3.dta
 Observations:        27,114
    Variables:            27                  17 Feb 2026 17:23
-------------------------------------------------------------------------------------------------------------------------------------
Variable      Storage   Display    Value
    name         type    format    label      Variable label
-------------------------------------------------------------------------------------------------------------------------------------
PAT_MRN_ID      long    %14.0g                Medical record number
Hosp_Adm        double  %tc                   Hospital admission date time
Hosp_DC         double  %tc                   Hospital discharge date time
Delivery        double  %tc                   Delivery date/ time
MaternalAge     byte    %10.0g                Maternal age (years)
Parity          byte    %10.0g                Parity
QuanBloodLoss   long    %12.0g                Quantitative blood loss (ml)
EstBloodLoss    long    %12.0g                Estimated blood loss (ml)
AnesHCPCS       long    %14.0f                CPT for billing anesthesia care
bAnesDeliv      int     %10.0f                Anesthesia care for delivery, 0=No anesthesia clinician
Hgb_Adm         double  %10.1f                Hemoglobin g/dl
AnesStartBeforeAtHgb
                double  %tc                   Case Start Datetime, but only if before/at time of Hgb check
AnesEndPlus2Hr  double  %tc                   Case Stop Datetime + 2 hr; Dexter F 18 page 1655 last paragraph
HrsDifference   double  %10.0g                Hgb Time checked in hours after (+) or before(-) admission
RBCtime         double  %tc                   Date/Time start RBC transfusion
bAnesTrans      int     %10.0f                Anesthesia care when transfusion started
N_units         int     %8.0g                 RBC units transfused among patients with Hgb measured
N_units_Anes    int     %8.0g                 RBC units transfused during anesthesia among patients with Hgb measured
Any_RBC_units   byte    %8.0g                 0 or 1 = RBC
Any_RBC_Anes    byte    %8.0g                 0 or 1 = RBC started during anesthesia care
RBC_GE2         byte    %8.0g                 0 or 1 = 2+ units RBC
RBC_GE2_Anes    byte    %8.0g                 0 or 1 = 2+ units RBC started during anesthesia care
RBCtimeHr       double  %10.0g                RBC first unit, if any, transfused relative to time of delivery
Hgb_Decile      byte    %8.0g                 10 quantiles of Hgb_Adm
MedianHgb       double  %10.0g                Median Hgb g/dl of decile
Weight_kg       double  %6.1f                 Weight (kg) most recent before delivery
AllowBloodLoss  double  %10.0g                Allowable Blood Loss (mL) - Russo Formula
-------------------------------------------------------------------------------------------------------------------------------------
Sorted by: PAT_MRN_ID  Hosp_Adm
```

```
.
. generate byte bAnemic_Adm = 0

. replace       bAnemic_Adm = 1 if Hgb_Adm < 11
(5,203 real changes made)

. tabulate bAnemic_Adm // Figure 1 legend

bAnemic_Adm |      Freq.     Percent        Cum.
------------+-----------------------------------
          0 |     21,911       80.81       80.81
          1 |      5,203       19.19      100.00
------------+-----------------------------------
      Total |     27,114      100.00

.
. generate int N_units_not_Anes = N_units - N_units_Anes

.
. ranksum                          N_units,          by(bAnemic_Adm) porder

Two-sample Wilcoxon rank-sum (Mann-Whitney) test

 bAnemic_Adm |      Obs    Rank sum    Expected
-------------+---------------------------------
           0 |    21911   2.920e+08   2.971e+08
           1 |     5203    75619733    70539673
-------------+---------------------------------
    Combined |    27114   3.676e+08   3.676e+08

Unadjusted variance   2.576e+11
Adjustment for ties  -2.228e+11
                     ----------
Adjusted variance     3.476e+10

H0: N_units(bAnemi~m==0) = N_units(bAnemi~m==1)
         z = -27.249
Prob > |z| =  0.0000

P{N_units(bAnemi~m==0) > N_units(bAnemi~m==1)} = 0.455

. ranksum                          N_units_not_Anes, by(bAnemic_Adm) porder // Figure 1 legend

Two-sample Wilcoxon rank-sum (Mann-Whitney) test

 bAnemic_Adm |      Obs    Rank sum    Expected
-------------+---------------------------------
           0 |    21911   2.924e+08   2.971e+08
           1 |     5203    75238720    70539673
-------------+---------------------------------
    Combined |    27114   3.676e+08   3.676e+08

Unadjusted variance   2.576e+11
Adjustment for ties  -2.266e+11
                     ----------
Adjusted variance     3.098e+10

H0: N~t_Anes(bAnemi~m==0) = N~t_Anes(bAnemi~m==1)
         z = -26.696
Prob > |z| =  0.0000

P{N~t_Anes(bAnemi~m==0) > N~t_Anes(bAnemi~m==1)} = 0.459

. by bAnemic_Adm, sort : summarize N_units

-------------------------------------------------------------------------------------------------------------------------------------
-> bAnemic_Adm = 0

    Variable |        Obs        Mean    Std. dev.       Min        Max
-------------+---------------------------------------------------------
     N_units |     21,911    .0551321    .4361254          0         25

-------------------------------------------------------------------------------------------------------------------------------------
-> bAnemic_Adm = 1

    Variable |        Obs        Mean    Std. dev.       Min        Max
-------------+---------------------------------------------------------
     N_units |      5,203    .2469729    .9873907          0         28


. ranksum                          N_units_Anes,     by(bAnemic_Adm) porder // Figure 1 legend

Two-sample Wilcoxon rank-sum (Mann-Whitney) test

 bAnemic_Adm |      Obs    Rank sum    Expected
-------------+---------------------------------
           0 |    21911   2.962e+08   2.971e+08
           1 |     5203    71399996    70539673
-------------+---------------------------------
    Combined |    27114   3.676e+08   3.676e+08

Unadjusted variance   2.576e+11
Adjustment for ties  -2.497e+11
                     ----------
Adjusted variance     7.870e+09

H0: N~s_Anes(bAnemi~m==0) = N~s_Anes(bAnemi~m==1)
         z = -9.698
Prob > |z| = 0.0000

P{N~s_Anes(bAnemi~m==0) > N~s_Anes(bAnemi~m==1)} = 0.492
```

```
. by bAnemic_Adm, sort : summarize N_units_Anes

-> bAnemic_Adm = 0

    Variable |        Obs        Mean    Std. dev.       Min        Max
-------------+---------------------------------------------------------
N_units_Anes |     21,911    .0122313    .1853515          0         14

-> bAnemic_Adm = 1

    Variable |        Obs        Mean    Std. dev.       Min        Max
-------------+---------------------------------------------------------
N_units_Anes |      5,203    .0355564    .2863088          0          8

.
. keep    Any_RBC_units PAT_MRN_ID                   Any_RBC_Anes bAnemic_Adm

. assert (Any_RBC_units == 0 | Any_RBC_units ==1) & (Any_RBC_Anes == 0 | Any_RBC_Anes == 1)

.
. generate int identifier_admission = _n // not using PAT_MRN_ID because >1 delivery for some patients

.
. generate byte Any_RBC_notAnes = 0

. replace       Any_RBC_notAnes = 1 if Any_RBC_units == 1 & Any_RBC_Anes == 0
(1,000 real changes made)

. drop                                   Any_RBC_units

.
. reshape long Any_RBC_, i(identifier_admission) j(group) string
(j = Anes notAnes)

Data                                   Wide   ->   Long
-----------------------------------------------------------------------------
Number of observations               27,114   ->   54,228
Number of variables                       5   ->   5
j variable (2 values)                         ->   group
xij variables:
           Any_RBC_Anes Any_RBC_notAnes   ->   Any_RBC_
-----------------------------------------------------------------------------

. rename       Any_RBC_ bAny_RBC

.
. encode group, gen(group_num)

. codebook          group_num // 1 Anes

-------------------------------------------------------------------------------------------------------------------------------
group_num                                                                                                          (unlabeled)
-------------------------------------------------------------------------------------------------------------------------------

                  Type: Numeric (long)
                 Label: group_num

                 Range: [1,2]                         Units: 1
         Unique values: 2                         Missing .: 0/54,228

            Tabulation: Freq.   Numeric  Label
                       27,114         1  Anes
                       27,114         2  notAnes

.
. sort  PAT_MRN_ID identifier_admission group_num  // Results, secondary analyses, paragraph 1

. xtgee bAny_RBC i.bAnemic_Adm##i.group_num, family(binomial) link(logit) corr(exchangeable) i(PAT_MRN_ID) robust

Iteration 1:  Tolerance = .0063206
Iteration 2:  Tolerance = .00005439
Iteration 3:  Tolerance = 1.814e-06
Iteration 4:  Tolerance = 4.671e-08

GEE population-averaged model                       Number of obs    =  54,228
Group variable: PAT_MRN_ID                          Number of groups =  21,304
Family: Binomial                                    Obs per group:
Link:   Logit                                                    min =       2
Correlation: exchangeable                                        avg =     2.5
                                                                 max =      14
                                                    Wald chi2(3)     = 1031.88
Scale parameter = 1                                 Prob > chi2      =  0.0000

                                         (Std. err. adjusted for clustering on PAT_MRN_ID)
---------------------------------------------------------------------------------
                |               Robust
       bAny_RBC | Coefficient  std. err.      z    P>|z|     [95% conf. interval]
----------------+----------------------------------------------------------------
  1.bAnemic_Adm |   1.114089   .1206124     9.24   0.000     .8776931    1.350485
                |
      group_num |
        notAnes |    1.12609   .0887055    12.69   0.000     .9522305     1.29995
                |
bAnemic_Adm#group_num |
      1#notAnes |   .4011889   .1379286     2.91   0.004     .1308539    .6715239
                |
          _cons |  -4.870367   .0772862   -63.02   0.000    -5.021845   -4.718889
---------------------------------------------------------------------------------
```

```
. margins, dydx(bAnemic_Adm) over(group_num)                    // probabilities for text

Average marginal effects                                Number of obs = 54,228
Model VCE: Robust

Expression: Pr(bAny_RBC != 0), predict()
dy/dx wrt:  1.bAnemic_Adm
Over:       group_num

------------------------------------------------------------------------------
             |            Delta-method
             |      dy/dx   std. err.      z    P>|z|     [95% conf. interval]
-------------+----------------------------------------------------------------
0.bAnemic_Adm|  (base outcome)
-------------+----------------------------------------------------------------
1.bAnemic_Adm|
   group_num |
        Anes |   .0152247   .0021543     7.07   0.000     .0110023    .0194471
     notAnes |   .0740702   .0043076    17.20   0.000     .0656276    .0825129
------------------------------------------------------------------------------
Note: dy/dx for factor levels is the discrete change from the base level.

. margins, dydx(bAnemic_Adm) over(group_num) pwcompare(effects) // P-value

Pairwise comparisons of average marginal effects

Model VCE: Robust                                       Number of obs = 54,228

Expression: Pr(bAny_RBC != 0), predict()
dy/dx wrt:  1.bAnemic_Adm
Over:       group_num

--------------------------------------------------------------------------------
                |   Contrast Delta-method    Unadjusted           Unadjusted
                |      dy/dx   std. err.      z    P>|z|     [95% conf. interval]
----------------+---------------------------------------------------------------
0.bAnemic_Adm   |  (base outcome)
----------------+---------------------------------------------------------------
1.bAnemic_Adm   |
      group_num |
notAnes vs Anes |   .0588455   .0048626    12.10   0.000      .049315     .068376
--------------------------------------------------------------------------------
Note: dy/dx for factor levels is the discrete change from the base level.

.
.
.
. *** Fourth paragraph of the Results, second secondary analyses paragraph *****************************************
. use "Temp_Table S3.dta", clear

.
. spearman AllowBloodLoss Hgb_Adm

Number of observations = 26,588
        Spearman's rho = 0.9861

Test of H0: AllowBloodLoss and Hgb_Adm are independent
                  Prob = 0.0000

.
. generate byte Any_RBC_NotAnes = 0

. replace       Any_RBC_NotAnes = 1 if N_units > N_units_Anes
(1,134 real changes made)

.
. summarize AllowBloodLoss

    Variable |        Obs        Mean    Std. dev.       Min        Max
-------------+---------------------------------------------------------
AllowBlood~s |     26,588    2488.881    395.6515          0   4217.522

. generate double ABL_Centered = ( AllowBloodLoss - r(mean) )/1000
(526 missing values generated)

. label var       ABL_Centered "ABL (Liters) centered at mean"

.
. generate int identifier_admission = _n

.
. keep PAT_MRN_ID Any_RBC_Anes Any_RBC_NotAnes ABL_Centered identifier_admission

.
. reshape long Any_RBC_, i(identifier_admission) j(group) string
(j = Anes NotAnes)

Data                               Wide   ->   Long
-----------------------------------------------------------------------------
Number of observations           27,114   ->   54,228
Number of variables                   5   ->   5
j variable (2 values)                     ->   group
xij variables:
           Any_RBC_Anes Any_RBC_NotAnes   ->   Any_RBC_
-----------------------------------------------------------------------------
```

```
. rename       Any_RBC_ bRBC

. encode group, gen(group_num) // 1 = Anes, 2 = NotAnes

.
. sort  PAT_MRN_ID identifier_admission group_num

. xtgee bRBC i.group_num##c.ABL_Centered, family(binomial) link(logit) corr(exchangeable) i(PAT_MRN_ID) robust nolog

GEE population-averaged model                       Number of obs    =  53,176
Group variable: PAT_MRN_ID                          Number of groups =  20,782
Family: Binomial                                    Obs per group:
Link:   Logit                                                    min =       2
Correlation: exchangeable                                        avg =     2.6
                                                                 max =      14
                                                    Wald chi2(3)     = 1473.03
Scale parameter = 1                                 Prob > chi2      =  0.0000

                                        (Std. err. adjusted for clustering on PAT_MRN_ID)
```

bRBC	Coefficient	Robust std. err.	z	P>|z|	[95% conf.	interval]
group_num						
NotAnes	1.29163	.0659561	19.58	0.000	1.162359	1.420902
ABL_Centered	-1.409353	.1171882	-12.03	0.000	-1.639037	-1.179668
group_num#c.ABL_Centered						
NotAnes	-.4662996	.1231654	-3.79	0.000	-.7076993	-.2249
_cons	-4.664333	.0676786	-68.92	0.000	-4.796981	-4.531685

```
.
. margins, dydx(ABL_Centered) over(group_num) post // slopes per 1 liter allowable blood loss

Average marginal effects                               Number of obs = 53,176
Model VCE: Robust

Expression: Pr(bRBC != 0), predict()
dy/dx wrt:  ABL_Centered
Over:       group_num
```

	dy/dx	Delta-method std. err.	z	P>|z|	[95% conf.	interval]
ABL_Centered						
group_num						
Anes	-.0155072	.0015037	-10.31	0.000	-.0184544	-.01256
NotAnes	-.0738002	.002827	-26.11	0.000	-.0793411	-.0682593

```
. nlcom (Fold_Difference: _b[2.group_num] / _b[1.group_num]), level(99)

Fold_Diffe~e: _b[2.group_num] / _b[1.group_num]
```

	Coefficient	Std. err.	z	P>|z|	[99% conf.	interval]
Fold_Difference	4.75909	.4729658	10.06	0.000	3.540811	5.977369

```
. matrix list r(table) // for the P-value

r(table)[9,1]
        Fold_Diffe~e
     b     4.7590898
    se     .47296576
     z     10.062229
pvalue     8.114e-24
    ll     3.5408108
    ul     5.9773689
    df             .
  crit     2.5758293
 eform             0

.
.
.
. *** Fifth paragraph of the Results, fourth paragraph of the secondary analyses ***********************************
. use "Temp_Table S3.dta", clear

.
. drop if  Any_RBC_units == 0
(25,835 observations deleted)

.
. generate byte bAnemic = 0

. replace       bAnemic = 1 if Hgb_Adm < 11
(619 real changes made)

.
. clonevar N_units_Anes_Not_Anemic = N_units_Anes
```

```
. replace  N_units_Anes_Not_Anemic = 0 if bAnemic == 1
(117 real changes made)

. ratio (N_units_Anes_Not_Anemic/N_units_Anes), level(99)

Ratio estimation                        Number of obs = 1,279

     _ratio_1: N_units_Anes_Not_Anemic/N_units_Anes

--------------------------------------------------------------
             |             Linearized
             |      Ratio   std. err.     [99% conf. interval]
-------------+------------------------------------------------
    _ratio_1 |   .5916115   .0367969      .4966872    .6865358
--------------------------------------------------------------

. display "Among all peripartum RBC units started during anesthesia care, for " %4.1f 100*r(table)[1,1] ///
>         "% of units, the patient was not anemic (Hgb ≥ 11 g/dL) upon admission."
Among all peripartum RBC units started during anesthesia care, for 59.2% of units, the patient was not anemic (Hgb ≥ 11 g/dL) upon a
> dmission.

. summarize N_units_Anes_Not_Anemic

    Variable |        Obs        Mean    Std. dev.       Min        Max
-------------+---------------------------------------------------------
N_units_An~c |      1,279    .2095387    .7400057          0         14

. display "Total for numerator of fraction = "   r(N)*r(mean)
Total for numerator of fraction = 268

. summarize N_units_Anes

    Variable |        Obs        Mean    Std. dev.       Min        Max
-------------+---------------------------------------------------------
N_units_Anes |      1,279     .354183    .8971155          0         14

. display "Total for denominator of fraction = " r(N)*r(mean)
Total for denominator of fraction = 453

.
. tabulate bAnesTrans bAnemic, row

+----------------+
| Key            |
|----------------|
|   frequency    |
| row percentage |
+----------------+

Anesthesia |
 care when |
transfusio |       bAnemic
 n started |         0          1 |     Total
-----------+----------------------+----------
         0 |       505        516 |     1,021 
           |     49.46      50.54 |    100.00 
-----------+----------------------+----------
         1 |       155        103 |       258 
           |     60.08      39.92 |    100.00 
-----------+----------------------+----------
     Total |       660        619 |     1,279 
           |     51.60      48.40 |    100.00 

.
.
.
. *** Table 2, timing of RBC transfusion among all patients, seventh paragraph results  ****************************
. use "Temp_Table 2.dta", clear

.
. describe, fullnames

Contains data from Temp_Table 2.dta
 Observations:        30,591
    Variables:            10                  17 Feb 2026 17:23
------------------------------------------------------------------------------------------------------------------------------------
Variable      Storage   Display    Value
    name         type    format    label      Variable label
------------------------------------------------------------------------------------------------------------------------------------
PAT_MRN_ID      long    %14.0g                Medical record number
Hosp_Adm        double  %tc                   Hospital admission date time
Hosp_DC         double  %tc                   Hospital discharge date time
Delivery        double  %tc                   Delivery date/ time
MaternalAge     byte    %10.0g                Maternal age (years)
Parity          byte    %10.0g                Parity
QuanBloodLoss   long    %12.0g                Quantitative blood loss (ml)
EstBloodLoss    long    %12.0g                Estimated blood loss (ml)
AnesHCPCS       long    %14.0f                CPT for billing anesthesia care
bAnesDeliv      int     %10.0f                Anesthesia care for delivery, 0=No anesthesia clinician
------------------------------------------------------------------------------------------------------------------------------------
Sorted by: PAT_MRN_ID  Hosp_Adm

.
. assert _N == 30591 // deliveries

. joinby PAT_MRN_ID using "Temp_Transfusions", unmatched(both) // RBC transfusion data
```

```
. drop if    _merge == 1
(28,831 observations deleted)

.
. assert RBCtime != . // because have deleted the patients without transfusion

. drop if (RBCtime < Hosp_Adm | RBCtime > Hosp_DC) // records joined and from different admissions (deliveries)
(900 observations deleted)

.
. sort PAT_MRN_ID Hosp_Adm RBCtime

. generate double RBCtimeHr = (RBCtime - Delivery)/ (1000*60*60)

. label variable  RBCtimeHr "RBC unit transfused relative to time of delivery"

.
. recode RBCtimeHr (min/-1 = 1) (-1/2 = 2) (2/4 = 3) (4/12 = 4) (12/24 = 5) (24/48 = 6) (48/max = 7), gen(RBCHrCat)
(2,588 differences between RBCtimeHr and RBCHrCat)

. label define RBCHrCat_labels  ///
>     1 "≤-1 hr before"         ///
>     2 ">-1 to  ≤2 hr"         ///
>     3 ">2 to  ≤4 hr"          ///
>     4 ">4 to ≤12 hr"          ///
>     5 ">12 to ≤24 hr"         ///
>     6 ">24 to ≤48 hr"         ///
>     7 ">48 hr after", modify   // labels with modify to prevent error that already defined

. label values RBCHrCat RBCHrCat_labels

. tabulate     RBCHrCat // units, not patients
```

RECODE of RBCtimeHr (RBC unit transfused relative to time of delivery)	Freq.	Percent	Cum.
≤-1 hr before	146	5.64	5.64
>-1 to ≤2 hr	702	27.10	32.74
>2 to ≤4 hr	245	9.46	42.20
>4 to ≤12 hr	303	11.70	53.90
>12 to ≤24 hr	340	13.13	67.03
>24 to ≤48 hr	489	18.88	85.91
>48 hr after	365	14.09	100.00
Total	2,590	100.00	

```
. tabulate     RBCHrCat if bAnesTrans == 0
```

RECODE of RBCtimeHr (RBC unit transfused relative to time of delivery)	Freq.	Percent	Cum.
≤-1 hr before	134	6.30	6.30
>-1 to ≤2 hr	334	15.70	21.99
>2 to ≤4 hr	195	9.16	31.16
>4 to ≤12 hr	277	13.02	44.17
>12 to ≤24 hr	336	15.79	59.96
>24 to ≤48 hr	489	22.98	82.94
>48 hr after	363	17.06	100.00
Total	2,128	100.00	

```
. tabulate     RBCHrCat if bAnesTrans == 1
```

RECODE of RBCtimeHr (RBC unit transfused relative to time of delivery)	Freq.	Percent	Cum.
≤-1 hr before	12	2.60	2.60
>-1 to ≤2 hr	368	79.65	82.25
>2 to ≤4 hr	50	10.82	93.07
>4 to ≤12 hr	26	5.63	98.70
>12 to ≤24 hr	4	0.87	99.57
>48 hr after	2	0.43	100.00
Total	462	100.00	

```
.
. generate byte  bLE1BeforeORGT4After = 0 if RBCHrCat != .

. replace        bLE1BeforeORGT4After = 1 if RBCHrCat != . & (RBCHrCat == 1 | RBCHrCat > 3)
(1,643 real changes made)
```

```
. ci proportions bLE1BeforeORGT4After, level(99)

                                                         Binomial exact
    Variable |        Obs  Proportion    Std. err.       [99% conf. interval]
-------------+---------------------------------------------------------------
bLE1Before~r |      2,590    .6343629    .0094633        .6095623    .6586651

.
. display "Among all peripartum RBC units, " %4.0f 100*r(proportion) "% (" r(proportion)*r(N) ///
>         "/" r(N) ")" _n "are transfused ≤-1 hr before or >4 hr after delivery."              // _n, line break
Among all peripartum RBC units,   63% (1643/2590)
are transfused ≤-1 hr before or >4 hr after delivery.

.
. ci proportions bLE1BeforeORGT4After if bAnesTrans == 1, level(99) // Table 2 footnote e

                                                         Binomial exact
    Variable |        Obs  Proportion    Std. err.       [99% conf. interval]
-------------+---------------------------------------------------------------
bLE1Before~r |        462    .0952381    .0136569        .0633117    .1357962

. display "Among all peripartum RBC units during anesthesia care, " %4.0f 100*(1-r(proportion)) "% ("  ///
>          (1-r(proportion))*r(N) "/" r(N) ")" _n "are transfused ≤-1 hr before or >4 hr after delivery."
Among all peripartum RBC units during anesthesia care,   90% (418/462)
are transfused ≤-1 hr before or >4 hr after delivery.

.     * seventh paragraph Results (Table 2)
.
. sort PAT_MRN_ID Hosp_Adm RBCtime // repeating by patient rather than preceding by unit

.                                  // however in the table presented as patients first
. replace bLE1BeforeORGT4After = . if PAT_MRN_ID == PAT_MRN_ID[_n-1] & Hosp_Adm == Hosp_Adm[_n-1]
(1,261 real changes made, 1,261 to missing)

. replace            RBCHrCat  = . if PAT_MRN_ID == PAT_MRN_ID[_n-1] & Hosp_Adm == Hosp_Adm[_n-1]
(1,261 real changes made, 1,261 to missing)

. replace            RBCtimeHr = . if PAT_MRN_ID == PAT_MRN_ID[_n-1] & Hosp_Adm == Hosp_Adm[_n-1]
(1,261 real changes made, 1,261 to missing)

. label variable     RBCtimeHr "RBC first unit transfused relative to time of delivery"

.
. tabulate     RBCHrCat                    // patients

    RECODE of |
    RBCtimeHr |
    (RBC unit |
   transfused |
  relative to |
      time of |
    delivery) |      Freq.     Percent        Cum.
--------------+-----------------------------------
≤-1 hr before |         78        5.87        5.87
>-1 to  ≤2 hr |        363       27.31       33.18
 >2 to  ≤4 hr |        148       11.14       44.32
 >4 to ≤12 hr |        143       10.76       55.08
>12 to ≤24 hr |        166       12.49       67.57
>24 to ≤48 hr |        272       20.47       88.04
 >48 hr after |        159       11.96      100.00
--------------+-----------------------------------
        Total |      1,329      100.00

. tabulate     RBCHrCat if bAnesTrans == 0

    RECODE of |
    RBCtimeHr |
    (RBC unit |
   transfused |
  relative to |
      time of |
    delivery) |      Freq.     Percent        Cum.
--------------+-----------------------------------
≤-1 hr before |         69        6.48        6.48
>-1 to  ≤2 hr |        146       13.71       20.19
 >2 to  ≤4 hr |        126       11.83       32.02
 >4 to ≤12 hr |        130       12.21       44.23
>12 to ≤24 hr |        165       15.49       59.72
>24 to ≤48 hr |        272       25.54       85.26
 >48 hr after |        157       14.74      100.00
--------------+-----------------------------------
        Total |      1,065      100.00

. tabulate     RBCHrCat if bAnesTrans == 1 // counts not same as Table 1 because using first unit

    RECODE of |
    RBCtimeHr |
    (RBC unit |
   transfused |
  relative to |
      time of |
    delivery) |      Freq.     Percent        Cum.
--------------+-----------------------------------
≤-1 hr before |          9        3.41        3.41
>-1 to  ≤2 hr |        217       82.20       85.61
 >2 to  ≤4 hr |         22        8.33       93.94
 >4 to ≤12 hr |         13        4.92       98.86
>12 to ≤24 hr |          1        0.38       99.24
 >48 hr after |          2        0.76      100.00
--------------+-----------------------------------
        Total |        264      100.00
```

```
.
. ci proportions bLE1BeforeORGT4After, level(99)

                                                        Binomial exact
    Variable |        Obs  Proportion    Std. err.       [99% conf. interval]
-------------+---------------------------------------------------------------
bLE1Before~r |      1,329    .6155004    .0133444        .5803816    .6497814

. display "Among peripartum patients receiving ≥1 RBC unit, " %4.1f 100*r(proportion) ///
>         "% (" r(proportion)*r(N) "/" r(N) ")"                                       ///
>         _n "receive their first (or only) unit ≤-1 hr before or >4 hr after delivery"
Among peripartum patients receiving ≥1 RBC unit, 61.6% (818/1329)
receive their first (or only) unit ≤-1 hr before or >4 hr after delivery

.
. ci proportions bLE1BeforeORGT4After if bAnesTrans == 1, exact level(99)

                                                        Binomial exact
    Variable |        Obs  Proportion    Std. err.       [99% conf. interval]
-------------+---------------------------------------------------------------
bLE1Before~r |        264     .094697    .0180203        .0540563    .1505842

. display "Among peripartum patients receiving ≥1 RBC unit during anesthesia care, " %4.1f 100*r(proportion) ///
>         "% (" r(proportion)*r(N)                                                                        ///
>         "/" r(N) ")" _n "receive their first (or only) unit ≤-1 hr before or >4 hr after delivery"
Among peripartum patients receiving ≥1 RBC unit during anesthesia care,  9.5% (25/264)
receive their first (or only) unit ≤-1 hr before or >4 hr after delivery

.
.
.
. *** Table 3, patients with Hgb initially and after delivery, eighth paragraph results  ***************************
. use "Temp_Table S4.dta", clear

.
. describe, fullnames

Contains data from Temp_Table S4.dta
 Observations:         4,817
    Variables:            26                  17 Feb 2026 17:23
-----------------------------------------------------------------------------------------------------------------------------------
Variable      Storage   Display    Value
    name         type    format    label      Variable label
-----------------------------------------------------------------------------------------------------------------------------------
PAT_MRN_ID      long    %14.0g                Medical record number
Hosp_Adm        double  %tc                   Hospital admission date time
Hosp_DC         double  %tc                   Hospital discharge date time
Delivery        double  %tc                   Delivery date/ time
MaternalAge     byte    %10.0g                Maternal age (years)
Parity          byte    %10.0g                Parity
QuanBloodLoss   long    %12.0g                Quantitative blood loss (ml)
EstBloodLoss    long    %12.0g                Estimated blood loss (ml)
AnesHCPCS       long    %14.0f                CPT for billing anesthesia care
bAnesDeliv      int     %10.0f                Anesthesia care for delivery, 0=No anesthesia clinician
Hgb_DC          double  %10.1f                Hemoglobin g/dl
AnesStartBeforeAtHgb
                double  %tc                   Case Start Datetime, but only if before/at time of Hgb check
AnesEndPlus2Hr  double  %tc                   Case Stop Datetime + 2 hr; Dexter F 18 page 1655 last paragraph
Hgb_Adm         double  %10.1f                Hemoglobin g/dl
HrsDifference   double  %10.0g
N_units         int     %8.0g                 RBC units transfused among patients with Hgb measured
N_units_Anes    int     %8.0g                 RBC units transfused during anesthesia care among patients with Hgb measured
Blood_loss      long    %12.0g                Quantitative else estimated blood loss at delivery, mL
Any_RBC_units   byte    %8.0g                 0 or 1 = RBC
Any_RBC_Anes    byte    %8.0g                 0 or 1 = RBC started during anesthesia care
RBC_GE2         byte    %8.0g                 0 or 1 = 2+ units RBC
RBC_GE2_Anes    byte    %8.0g                 0 or 1 = 2+ units RBC started during anesthesia care
Hgb_Decile_DC   byte    %8.0g                 10 quantiles of Hgb_DC
MedianHgb       double  %10.0g                Median Hgb g/dl at discharge of decile
Weight_kg       double  %6.1f                 Weight (kg) most recent before delivery
AllowBloodLoss  double  %10.0g                Allowable Blood Loss (mL) - Russo Formula
-----------------------------------------------------------------------------------------------------------------------------------
Sorted by: PAT_MRN_ID  Hosp_Adm

.
. clonevar Any_RBC_Not_Anes = Any_RBC_units

. assert   N_units - N_units_Anes >= 0

. replace  Any_RBC_Not_Anes = 0 if N_units - N_units_Anes == 0
(130 real changes made)

.
. generate byte bHgb_DC_LT_9 = 0

. replace       bHgb_DC_LT_9 = 1 if Hgb_DC <9
(862 real changes made)

.
. summarize Hgb_Adm      if Any_RBC_Anes == 0 & Any_RBC_Not_Anes == 0, detail

                       Hemoglobin g/dl
-------------------------------------------------------------
      Percentiles      Smallest
 1%          8.9            7.4
 5%          9.9            7.7
10%         10.4            7.8       Obs               3,809
25%         11.2            7.9       Sum of wgt.       3,809

50%           12                      Mean           11.92762
                        Largest       Std. dev.      1.224152
75%         12.7           16.5
90%         13.4           17.1       Variance       1.498549
95%         13.8             18       Skewness        .105001
99%         14.7           22.6       Kurtosis       4.828236
```

```
. summarize Hgb_DC       if Any_RBC_Anes == 0 & Any_RBC_Not_Anes == 0, detail

                       Hemoglobin g/dl
-------------------------------------------------------------
      Percentiles      Smallest
 1%          7.7            5.7
 5%          8.6            5.9
10%            9            6.1       Obs               3,809
25%          9.7            6.4       Sum of wgt.       3,809

50%         10.6                      Mean           10.61433
                        Largest       Std. dev.      1.310033
75%         11.5           15.1
90%         12.3           15.2       Variance       1.716186
95%         12.8           15.8       Skewness       .2525901
99%         13.7           20.6       Kurtosis       3.851457

. tabulate  bHgb_DC_LT_9 if Any_RBC_Anes == 0 & Any_RBC_Not_Anes == 0

bHgb_DC_LT_ |
          9 |      Freq.     Percent        Cum.
------------+-----------------------------------
          0 |      3,455       90.71       90.71
          1 |        354        9.29      100.00
------------+-----------------------------------
      Total |      3,809      100.00

. summarize Blood_loss   if Any_RBC_Anes == 0 & Any_RBC_Not_Anes == 0, detail

          Quantitative else estimated blood loss at
                        delivery, mL
-------------------------------------------------------------
      Percentiles      Smallest
 1%           81              5
 5%          200              5
10%          294             12       Obs               3,704
25%          511             19       Sum of wgt.       3,704

50%          900                      Mean           941.3116
                        Largest       Std. dev.      526.3518
75%         1284           3238
90%         1637           3352       Variance       277046.2
95%         1857           3423       Skewness       .5971881
99%         2347           3716       Kurtosis       3.396029

.
. summarize Hgb_Adm      if Any_RBC_Anes == 0 & Any_RBC_Not_Anes == 1, detail

                       Hemoglobin g/dl
-------------------------------------------------------------
      Percentiles      Smallest
 1%          7.5            6.9
 5%          8.4            6.9
10%          9.1            6.9       Obs                 751
25%         10.1            6.9       Sum of wgt.         751

50%         11.1                      Mean           10.98043
                        Largest       Std. dev.      1.463795
75%           12           14.8
90%         12.6           14.9       Variance       2.142696
95%         13.1           15.2       Skewness       -.154329
99%         14.4           17.1       Kurtosis       3.265186

. summarize Hgb_DC       if Any_RBC_Anes == 0 & Any_RBC_Not_Anes == 1, detail

                       Hemoglobin g/dl
-------------------------------------------------------------
      Percentiles      Smallest
 1%          6.1            4.2
 5%          6.9            4.5
10%          7.4            5.9       Obs                 751
25%            8            5.9       Sum of wgt.         751

50%          8.7                      Mean           8.879893
                        Largest       Std. dev.       1.33293
75%          9.7           13.2
90%         10.7           13.2       Variance       1.776702
95%         11.1           13.3       Skewness       .3736978
99%         12.4           13.4       Kurtosis       3.461537

. tabulate  bHgb_DC_LT_9 if Any_RBC_Anes == 0 & Any_RBC_Not_Anes == 1

bHgb_DC_LT_ |
          9 |      Freq.     Percent        Cum.
------------+-----------------------------------
          0 |        333       44.34       44.34
          1 |        418       55.66      100.00
------------+-----------------------------------
      Total |        751      100.00

. summarize Blood_loss   if Any_RBC_Anes == 0 & Any_RBC_Not_Anes == 1, detail

          Quantitative else estimated blood loss at
                        delivery, mL
-------------------------------------------------------------
      Percentiles      Smallest
 1%          114             45
 5%          475             46
10%          700             50       Obs                 694
25%         1160             75       Sum of wgt.         694

50%       1601.5                      Mean           1737.965
                        Largest       Std. dev.      1586.336
75%         2069           8121
90%         2578           9755       Variance        2516463
95%         3025          10000       Skewness       13.92709
99%         5486          35000       Kurtosis        282.181
```

```
.
. summarize Hgb_Adm      if Any_RBC_Anes == 1 & Any_RBC_Not_Anes == 1, detail

                       Hemoglobin g/dl
-------------------------------------------------------------
      Percentiles      Smallest
 1%            7            5.5
 5%          7.8              7
10%          8.3              7       Obs                 127
25%          9.7            7.4       Sum of wgt.         127

50%         11.2                      Mean           10.94173
                        Largest       Std. dev.      1.800372
75%         12.2           13.9
90%         13.1           14.6       Variance        3.24134
95%         13.4           14.6       Skewness      -.4143432
99%         14.6           14.8       Kurtosis        2.85547

. summarize Hgb_DC       if Any_RBC_Anes == 1 & Any_RBC_Not_Anes == 1, detail

                       Hemoglobin g/dl
-------------------------------------------------------------
      Percentiles      Smallest
 1%          6.1            5.1
 5%          6.9            6.1
10%          7.2            6.3       Obs                 127
25%          7.9            6.4       Sum of wgt.         127

50%          8.8                      Mean           8.825984
                        Largest       Std. dev.       1.28756
75%          9.7           11.3
90%         10.7           11.5       Variance       1.657812
95%           11           11.5       Skewness       .0374992
99%         11.5           11.9       Kurtosis       2.799349

. tabulate  bHgb_DC_LT_9 if Any_RBC_Anes == 1 & Any_RBC_Not_Anes == 1

bHgb_DC_LT_ |
          9 |      Freq.     Percent        Cum.
------------+-----------------------------------
          0 |         56       44.09       44.09
          1 |         71       55.91      100.00
------------+-----------------------------------
      Total |        127      100.00

. summarize Blood_loss   if Any_RBC_Anes == 1 & Any_RBC_Not_Anes == 1, detail

          Quantitative else estimated blood loss at
                        delivery, mL
-------------------------------------------------------------
      Percentiles      Smallest
 1%          559            400
 5%         1149            559
10%         1365            565       Obs                 127
25%         2021            787       Sum of wgt.         127

50%         2500                      Mean           2735.945
                        Largest       Std. dev.      1219.588
75%         3200           5717
90%         4461           6655       Variance        1487396
95%         5051           6836       Skewness        1.13945
99%         6836           7169       Kurtosis        5.00447

.
. summarize Hgb_Adm      if Any_RBC_Anes == 1 & Any_RBC_Not_Anes == 0, detail

                       Hemoglobin g/dl
-------------------------------------------------------------
      Percentiles      Smallest
 1%          7.6            7.4
 5%            8            7.6
10%          8.7            7.7       Obs                 130
25%         10.3              8       Sum of wgt.         130

50%        11.55                      Mean           11.27385
                        Largest       Std. dev.      1.570837
75%         12.3           13.6
90%         12.9           14.1       Variance       2.467528
95%         13.4           14.4       Skewness      -.5765097
99%         14.4             15       Kurtosis       2.940231

. summarize Hgb_DC       if Any_RBC_Anes == 1 & Any_RBC_Not_Anes == 0, detail

                       Hemoglobin g/dl
-------------------------------------------------------------
      Percentiles      Smallest
 1%          7.4            4.4
 5%          8.1            7.4
10%         8.55            7.7       Obs                 130
25%          9.2            7.7       Sum of wgt.         130

50%         10.2                      Mean           10.16077
                        Largest       Std. dev.       1.34916
75%         11.1           12.7
90%         11.8           12.7       Variance       1.820232
95%         12.5           12.9       Skewness      -.4063304
99%         12.9           13.3       Kurtosis       4.466817
```

```
. tabulate  bHgb_DC_LT_9 if Any_RBC_Anes == 1 & Any_RBC_Not_Anes == 0

bHgb_DC_LT_ |
          9 |      Freq.     Percent        Cum.
------------+-----------------------------------
          0 |        111       85.38       85.38
          1 |         19       14.62      100.00
------------+-----------------------------------
      Total |        130      100.00

. summarize Blood_loss   if Any_RBC_Anes == 1 & Any_RBC_Not_Anes == 0, detail

          Quantitative else estimated blood loss at
                        delivery, mL
-------------------------------------------------------------
      Percentiles      Smallest
 1%          212             94
 5%          850            212
10%         1171            667       Obs                 122
25%         1618            738       Sum of wgt.         122

50%         2135                      Mean           2196.721
                        Largest       Std. dev.      1128.117
75%         2500           3617
90%         3138           6125       Variance        1272649
95%         3447           6400       Skewness       3.342286
99%         6400          10000       Kurtosis       22.40251

.
. display "Sum of denominators 3809 + 751 + 127 + 130 = " 3809 + 751 + 127 + 130 " = 4817 = " _N // footnote a
Sum of denominators 3809 + 751 + 127 + 130 = 4817 = 4817 = 4817

.
. summarize Hgb_DC if Any_RBC_Not_Anes == 1 // footnote to compare with Hamm RF et al. 2021, Footnote b

    Variable |        Obs        Mean    Std. dev.       Min        Max
-------------+---------------------------------------------------------
      Hgb_DC |        878    8.872096    1.325879        4.2       13.4

.
. generate byte bHgb_DC_GE_9 = . // first sentence of results, eighth paragraph
(4,817 missing values generated)

. replace       bHgb_DC_GE_9 = 1 if Hgb_DC >= 9 & Hgb_DC != .
(3,955 real changes made)

. replace       bHgb_DC_GE_9 = 0 if Hgb_DC <  9 & Hgb_DC != .
(862 real changes made)

. tabulate Any_RBC_Not_Anes bHgb_DC_GE_9 if Any_RBC_Anes == 1, exact row

+----------------+
| Key            |
|----------------|
|   frequency    |
| row percentage |
+----------------+

  0 or 1 = |      bHgb_DC_GE_9
       RBC |         0          1 |     Total
-----------+----------------------+----------
         0 |        19        111 |       130 
           |     14.62      85.38 |    100.00 
-----------+----------------------+----------
         1 |        71         56 |       127 
           |     55.91      44.09 |    100.00 
-----------+----------------------+----------
     Total |        90        167 |       257 
           |     35.02      64.98 |    100.00 

           Fisher's exact =                 0.000
   1-sided Fisher's exact =                 0.000

.
. capture drop  bHgb_DC_GE_9

. generate byte bHgb_DC_GE_9 = . // Variable used in the Results section
(4,817 missing values generated)

. replace       bHgb_DC_GE_9 = 0 if Any_RBC_Anes == 1 & Any_RBC_Not_Anes == 0
(130 real changes made)

. replace       bHgb_DC_GE_9 = 1 if Any_RBC_Anes == 1 & Any_RBC_Not_Anes == 0 & Hgb_DC >= 9
(111 real changes made)

. tabulate      bHgb_DC_GE_9                           // results eighth paragraph

bHgb_DC_GE_ |
          9 |      Freq.     Percent        Cum.
------------+-----------------------------------
          0 |         19       14.62       14.62
          1 |        111       85.38      100.00
------------+-----------------------------------
      Total |        130      100.00

.
```

```
. ranksum  N_units_Anes, by(bHgb_DC_GE_9) porder

Two-sample Wilcoxon rank-sum (Mann-Whitney) test

bHgb_DC_GE_9 |      Obs    Rank sum    Expected
-------------+---------------------------------
           0 |       19      1305.5      1244.5
           1 |      111      7209.5      7270.5
-------------+---------------------------------
    Combined |      130        8515        8515

Unadjusted variance    23023.25
Adjustment for ties    -6499.61
                     ----------
Adjusted variance      16523.64

H0: N_uni~es(bHgb~E_9==0) = N_uni~es(bHgb~E_9==1)
         z =  0.475
Prob > |z| = 0.6351
Exact prob = 0.6219

P{N_uni~es(bHgb~E_9==0) > N_uni~es(bHgb~E_9==1)} = 0.529

. tabulate N_units_Anes if  bHgb_DC_GE_9 == 0

  RBC units |
 transfused |
     during |
 anesthesia |
 care among |
   patients |
   with Hgb |
   measured |      Freq.     Percent        Cum.
------------+-----------------------------------
          1 |         11       57.89       57.89
          2 |          7       36.84       94.74
          5 |          1        5.26      100.00
------------+-----------------------------------
      Total |         19      100.00

. tabulate N_units_Anes if  bHgb_DC_GE_9 == 1         // larger % single unit (i.e., cannot explain)

  RBC units |
 transfused |
     during |
 anesthesia |
 care among |
   patients |
   with Hgb |
   measured |      Freq.     Percent        Cum.
------------+-----------------------------------
          1 |         71       63.96       63.96
          2 |         34       30.63       94.59
          3 |          3        2.70       97.30
          4 |          1        0.90       98.20
          6 |          1        0.90       99.10
         14 |          1        0.90      100.00
------------+-----------------------------------
      Total |        111      100.00

.
. ranksum   Blood_loss, by (bHgb_DC_GE_9) porder

Two-sample Wilcoxon rank-sum (Mann-Whitney) test

bHgb_DC_GE_9 |      Obs    Rank sum    Expected
-------------+---------------------------------
           0 |       15       865.5       922.5
           1 |      107      6637.5      6580.5
-------------+---------------------------------
    Combined |      122        7503        7503

Unadjusted variance    16451.25
Adjustment for ties       -1.09
                     ----------
Adjusted variance      16450.16

H0: Blood_~s(bHgb~E_9==0) = Blood_~s(bHgb~E_9==1)
         z = -0.444
Prob > |z| = 0.6567
Exact prob = 0.6611

P{Blood_~s(bHgb~E_9==0) > Blood_~s(bHgb~E_9==1)} = 0.464

. summarize Blood_loss if   bHgb_DC_GE_9 == 0, detail // larger

          Quantitative else estimated blood loss at
                        delivery, mL
-------------------------------------------------------------
      Percentiles      Smallest
 1%           94             94
 5%           94            898
10%          898           1017       Obs                  15
25%         1171           1171       Sum of wgt.          15

50%         1870                      Mean               2154
                        Largest       Std. dev.       1448.49
75%         2650           2650
90%         3188           2932       Variance        2098123
95%         6400           3188       Skewness       1.571215
99%         6400           6400       Kurtosis       6.085933
```

```
. summarize Blood_loss if   bHgb_DC_GE_9 == 1, detail

          Quantitative else estimated blood loss at
                         delivery, mL
-------------------------------------------------------------
      Percentiles      Smallest
 1%          667            212
 5%          850            667
10%         1291            738       Obs                 107
25%         1653            791       Sum of wgt.         107

50%         2152                      Mean            2202.71
                        Largest       Std. dev.      1084.129
75%         2500           3487
90%         3138           3617       Variance        1175335
95%         3447           6125       Skewness       3.832197
99%         6125          10000       Kurtosis       27.59276

.
. ranksum   Hgb_Adm, by(bHgb_DC_GE_9) porder          // results eighth paragraph "the admission hemoglobin"

Two-sample Wilcoxon rank-sum (Mann-Whitney) test

bHgb_DC_GE_9 |      Obs    Rank sum    Expected
-------------+---------------------------------
           0 |       19         759      1244.5
           1 |      111        7756      7270.5
-------------+---------------------------------
    Combined |      130        8515        8515

Unadjusted variance    23023.25
Adjustment for ties      -23.14
                     ----------
Adjusted variance      23000.11

H0: Hgb_Adm(bHgb~E_9==0) = Hgb_Adm(bHgb~E_9==1)
         z = -3.201
Prob > |z| = 0.0014
Exact prob = 0.0011

P{Hgb_Adm(bHgb~E_9==0) > Hgb_Adm(bHgb~E_9==1)} = 0.270

. summarize Hgb_Adm if  bHgb_DC_GE_9 == 0, detail

                       Hemoglobin g/dl
-------------------------------------------------------------
      Percentiles      Smallest
 1%          7.6            7.6
 5%          7.6            7.7
10%          7.7              8       Obs                  19
25%          8.2              8       Sum of wgt.          19

50%         10.2                      Mean                 10
                        Largest       Std. dev.      1.875278
75%         11.8           12.2
90%         12.9           12.2       Variance       3.516667
95%         13.3           12.9       Skewness       .2619894
99%         13.3           13.3       Kurtosis       1.751583

. summarize Hgb_Adm if  bHgb_DC_GE_9 == 1, detail     // explains, and matters more becuase infusion clinic

                       Hemoglobin g/dl
-------------------------------------------------------------
      Percentiles      Smallest
 1%            8            7.4
 5%          8.7              8
10%          9.6              8       Obs                 111
25%         10.7              8       Sum of wgt.         111

50%         11.7                      Mean           11.49189
                        Largest       Std. dev.      1.410682
75%         12.3           13.6
90%         12.9           14.1       Variance       1.990025
95%         13.5           14.4       Skewness      -.5874547
99%         14.4             15       Kurtosis        3.48612

.
. generate bCesarean = 0

. replace  bCesarean = 1 if AnesHCPCS == 59510 | AnesHCPCS == 59514 | AnesHCPCS == 59515 | ///
>                          AnesHCPCS == 59525 | AnesHCPCS == 59620 | AnesHCPCS == 59622
(2,934 real changes made)

.
. tabulate bCesarean if Any_RBC_Anes == 1 & Any_RBC_Not_Anes == 0

  bCesarean |      Freq.     Percent        Cum.
------------+-----------------------------------
          0 |         37       28.46       28.46
          1 |         93       71.54      100.00
------------+-----------------------------------
      Total |        130      100.00

. tabulate bCesarean if Any_RBC_Anes == 1 & Any_RBC_Not_Anes == 0 & bHgb_DC_GE_9 == 1

  bCesarean |      Freq.     Percent        Cum.
------------+-----------------------------------
          0 |         31       27.93       27.93
          1 |         80       72.07      100.00
------------+-----------------------------------
      Total |        111      100.00
```

```
. tabulate bCesarean if Any_RBC_Anes == 1 & Any_RBC_Not_Anes == 0 & bHgb_DC_GE_9 == 1 & ///
>                       Blood_loss < 2500 & Blood_loss != . & N_units == 1 // eighth paragraph results

  bCesarean |      Freq.     Percent        Cum.
------------+-----------------------------------
          0 |         20       35.71       35.71
          1 |         36       64.29      100.00
------------+-----------------------------------
      Total |         56      100.00

. summarize N_units

    Variable |        Obs        Mean    Std. dev.       Min        Max
-------------+---------------------------------------------------------
     N_units |      4,817    .4286901    1.226418          0         28

. display r(mean)*r(N)
2065

. summarize N_units if bCesarean == 1 & Any_RBC_Anes == 1 & Any_RBC_Not_Anes == 0 & bHgb_DC_GE_9 == 1 & ///
>                      Blood_loss < 2500 & Blood_loss != . & N_units == 1

    Variable |        Obs        Mean    Std. dev.       Min        Max
-------------+---------------------------------------------------------
     N_units |         36           1           0          1          1

. display r(mean)*r(N)
36

. display %4.1f 100*36/2065 "% (36/2065) units"
 1.7% (36/2065) units

. 
. * Repeating among patients with discharge hemoglobin >= 11
. tabulate bCesarean if Any_RBC_Anes == 1 & Any_RBC_Not_Anes == 0 & Hgb_DC >= 11

  bCesarean |      Freq.     Percent        Cum.
------------+-----------------------------------
          0 |         13       34.21       34.21
          1 |         25       65.79      100.00
------------+-----------------------------------
      Total |         38      100.00

. tabulate bCesarean if Any_RBC_Anes == 1 & Any_RBC_Not_Anes == 0 & Hgb_DC >= 11 & ///
>                       Blood_loss < 2500 & Blood_loss != . & N_units == 1

  bCesarean |      Freq.     Percent        Cum.
------------+-----------------------------------
          0 |          8       42.11       42.11
          1 |         11       57.89      100.00
------------+-----------------------------------
      Total |         19      100.00

. 
. 
. 
. *** Ninth paragraph of Results, audit criteria *******************************************************************
. use "Temp_Table 1.dta" , clear

. tabulate bAnesDeliv // because audit analysis is proportion of cases

 Anesthesia |
   care for |
  delivery, |
       0=No |
 anesthesia |
  clinician |      Freq.     Percent        Cum.
------------+-----------------------------------
          0 |      9,273       30.75       30.75
          1 |     20,887       69.25      100.00
------------+-----------------------------------
      Total |     30,160      100.00

. 
. use "Temp_Table S4.dta", clear

. assert Hgb_Adm != . & Hgb_DC != .

. 
. tabulate bAnesDeliv if (N_units > 0) & (N_units_Anes == N_units) & (Hgb_DC >= 9)

 Anesthesia |
   care for |
  delivery, |
       0=No |
 anesthesia |
  clinician |      Freq.     Percent        Cum.
------------+-----------------------------------
          0 |          7        6.31        6.31
          1 |        104       93.69      100.00
------------+-----------------------------------
      Total |        111      100.00

. display %4.1f 100* 104/20887 "% (104/20,887)"
 0.5% (104/20,887)
```

```
.
. tabulate bAnesDeliv if (N_units > 0) & (N_units_Anes == N_units) & (Hgb_DC >= 9) & (Blood_loss < 2500)

 Anesthesia |
   care for |
  delivery, |
       0=No |
 anesthesia |
  clinician |      Freq.     Percent        Cum.
------------+-----------------------------------
          0 |          5        6.33        6.33
          1 |         74       93.67      100.00
------------+-----------------------------------
      Total |         79      100.00

. display %4.1f 100* 74/20887 "% (74/20,887)"
 0.4% (74/20,887)

. tabulate N_units    if (N_units > 0) & (N_units_Anes == N_units) & (Hgb_DC >= 9) & (Blood_loss < 2500)

  RBC units |
 transfused |
      among |
   patients |
   with Hgb |
   measured |      Freq.     Percent        Cum.
------------+-----------------------------------
          1 |         56       70.89       70.89
          2 |         21       26.58       97.47
          3 |          1        1.27       98.73
          4 |          1        1.27      100.00
------------+-----------------------------------
      Total |         79      100.00

.
.
.
. *** Figure 1 and legend ******************************************************************************************
. use "Temp_Table S3.dta", clear

.
. capture drop Perc - CI_high_perc

. generate double Perc = .         // percentage any transfusion
(27,114 missing values generated)

. generate double Logit = .
(27,114 missing values generated)

. generate double CI_low_perc  = .
(27,114 missing values generated)

. generate double CI_high_perc = .
(27,114 missing values generated)

. forvalues i = 1/10 {
  2.     quietly ci proportions Any_RBC_units if Hgb_Decile == `i', wilson level(99)
  3.     quietly replace Perc = 100*   r(proportion)  if Hgb_Decile == `i' & Hgb_Decile != Hgb_Decile[_n-1]
  4.     quietly replace Logit = logit(r(proportion)) if Hgb_Decile == `i' & Hgb_Decile != Hgb_Decile[_n-1]
  5.     quietly replace CI_low_perc  = 100*r(lb)     if Hgb_Decile == `i' & Hgb_Decile != Hgb_Decile[_n-1]
  6.     quietly replace CI_high_perc = 100*r(ub)     if Hgb_Decile == `i' & Hgb_Decile != Hgb_Decile[_n-1]
  7. }

.
. twoway (scatter Perc MedianHgb, msymbol(O) mcolor(blue) msize(large))                              ///
>        (rcap CI_low_perc CI_high_perc MedianHgb, lcolor(ltblue) lwidth(medthick))                 ///
>        (scatter Logit MedianHgb, msymbol(o) mfcolor(red%50) mlcolor(red) yaxis(2) msize(large)) ///
>        (lfit Logit MedianHgb, lcolor(red) lwidth(medium) yaxis(2)),                                ///
>        ylabel(0(2)18) xlabel(9.5(0.5)14.5, format(%4.1f)) legend(off)                              ///
>        ytitle("Percentage (%) of patients with RBC transfusion", color(blue))                      ///
>        xtitle("Median hemoglobin (g/dL) at admission for delivery")                                ///
>        ytitle("Logit of proportion of patients with RBC transfusion", color(red) axis(2))          ///
>        ylabel(-5.0(0.5)-1.5, format(%4.1f) axis(2))

. graph export  "02 OB anemia clinic Transfusion Log.pdf", as(pdf) name("Graph") replace // currently not in paper
file 02 OB anemia clinic Transfusion Log.pdf saved as PDF format

.
. capture log close
```

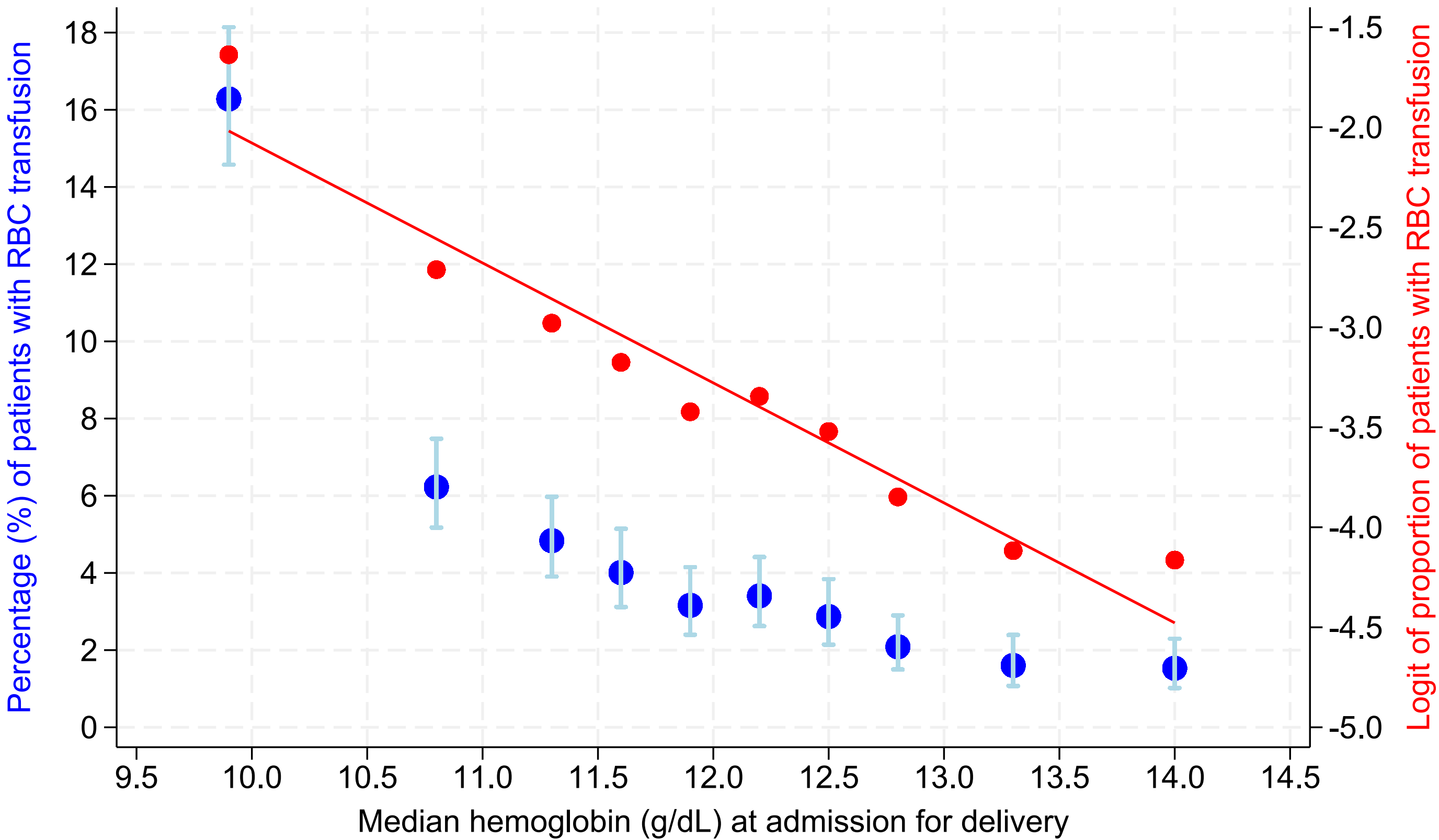

Percentage (%) of patients with RBC transfusion
0
2
4
6
8
10
12
14
16
18
Logit of proportion of patients with RBC transfusion
-1.5
-2.0
-2.5
-3.0
-3.5
-4.0
-4.5
-5.0
9.5
10.0
10.5
11.0
11.5
12.0
12.5
13.0
13.5
14.0
14.5
Median hemoglobin (g/dL) at admission for delivery

```
.
. * Graph that shows population percentage on left, as above, with % during anesthesia care on right
. capture drop Perc - CI_high_perc

. generate double Perc  = .
(27,114 missing values generated)

. generate double PercA = .
(27,114 missing values generated)

. generate double CI_low_perc   = .
(27,114 missing values generated)

. generate double CI_low_percA  = .
(27,114 missing values generated)

. generate double CI_high_perc  = .
(27,114 missing values generated)

. generate double CI_high_percA = .
(27,114 missing values generated)

. forvalues i = 1/10 {
  2.
.     quietly ci proportions Any_RBC_units if Hgb_Decile == `i', wilson level(99) // note Any_RBC_units
  3.     quietly replace Perc  = 100*   r(proportion)  if Hgb_Decile == `i' & Hgb_Decile != Hgb_Decile[_n-1]
  4.     quietly replace CI_low_perc   = 100*r(lb)     if Hgb_Decile == `i' & Hgb_Decile != Hgb_Decile[_n-1]
  5.     quietly replace CI_high_perc  = 100*r(ub)     if Hgb_Decile == `i' & Hgb_Decile != Hgb_Decile[_n-1]
  6.
.     quietly ci proportions Any_RBC_Anes  if Hgb_Decile == `i', wilson level(99) // note Any_RBC_Anes, anesthesia units
  7.     quietly replace PercA = 100*   r(proportion)  if Hgb_Decile == `i' & Hgb_Decile != Hgb_Decile[_n-1]
  8.     quietly replace CI_low_percA  = 100*r(lb)     if Hgb_Decile == `i' & Hgb_Decile != Hgb_Decile[_n-1]
  9.     quietly replace CI_high_percA = 100*r(ub)     if Hgb_Decile == `i' & Hgb_Decile != Hgb_Decile[_n-1]
 10. }

.
. clonevar MedianHgbA = MedianHgb

. replace  MedianHgbA = MedianHgb + 0.06 if MedianHgb > 13.0
(5,042 real changes made)

.
. twoway (scatter Perc MedianHgb, msymbol(O) mcolor(blue) msize(large))                              ///
>        (rcap CI_low_perc  CI_high_perc  MedianHgb, lcolor(ltblue) lwidth(medthick))                ///
>        (scatter PercA MedianHgbA, msymbol(o) mfcolor(red%50) mlcolor(red) yaxis(2) msize(vlarge))  ///
>        (rcap CI_low_percA CI_high_percA MedianHgbA, lcolor(red) yaxis(2) lwidth(medthick)),        ///
>        ylabel(0(2)18) xlabel(9.5(0.5)14.5, format(%4.1f)) legend(off)                              ///
>        ytitle("Percentage (%) of patients with RBC transfusion", color(blue))                      ///
>        xtitle("Median hemoglobin (g/dL) at admission for delivery")                                ///
>        ytitle("During anesthesia care" "Percentage (%) of patients with RBC transfusion",          ///
>               color(red) axis(2)) ylabel(0(2)18, axis(2))

. graph export "Out_Figure 1 v19.jpg"    , as(jpg) name("Graph") replace width(3600) height(2160) quality(100)
file Out_Figure 1 v19.jpg written in JPEG format

. graph export  "04 Figure 1 Export.pdf" , as(pdf) name("Graph") replace
file 04 Figure 1 Export.pdf saved as PDF format

.
. capture log close
```

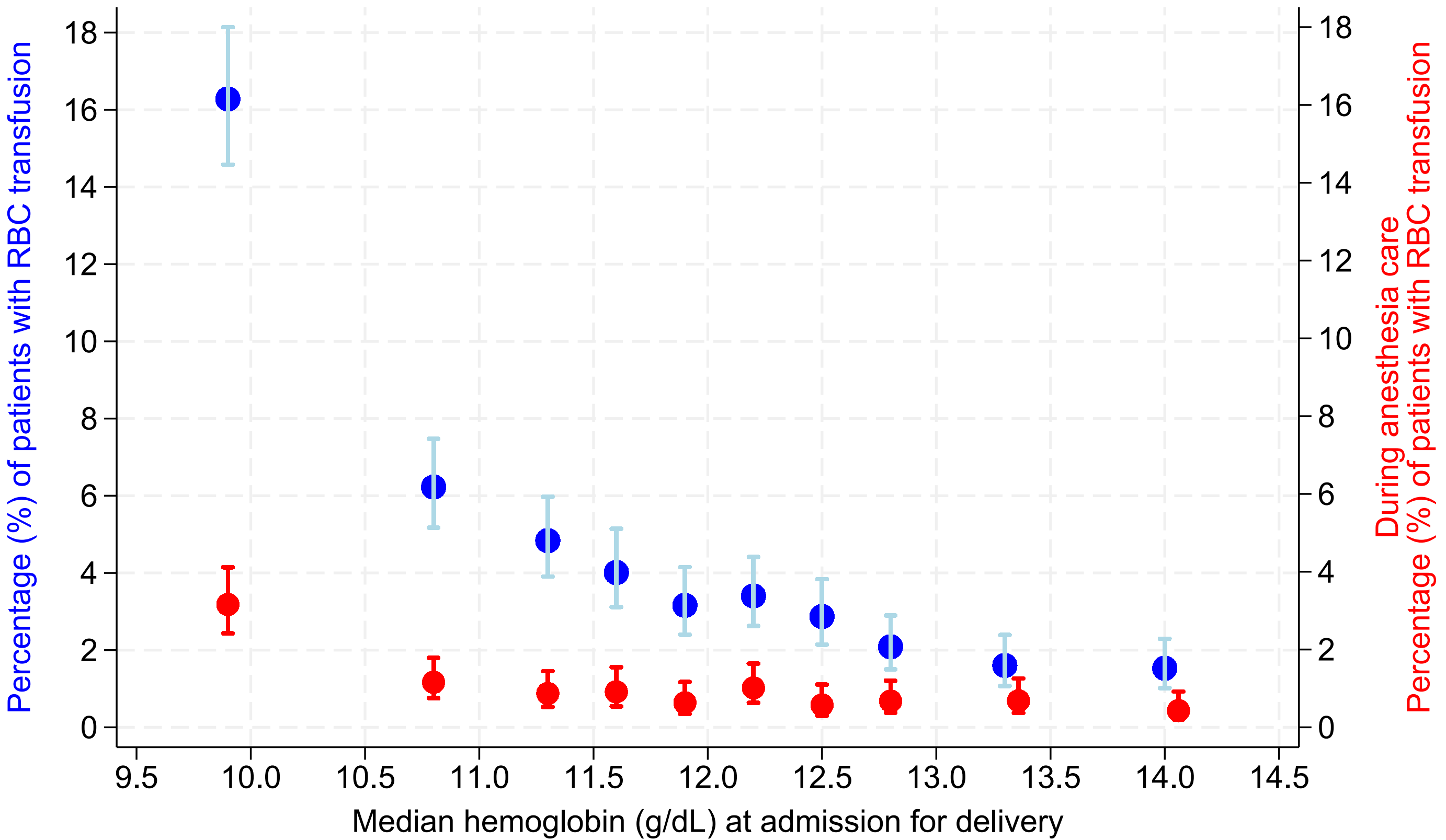

Percentage (%) of patients with RBC transfusion
During anesthesia care
Percentage (%) of patients with RBC transfusion
Median hemoglobin (g/dL) at admission for delivery
0
2
4
6
8
10
12
14
16
18
9.5
10.0
10.5
11.0
11.5
12.0
12.5
13.0
13.5
14.0
14.5

```
      name:  <unnamed>
       log:  C:\Frank\Ituk Toledo Epstein OB anemia clinic\05 OB anemia clinic Transfusion Log.smcl
  log type:  smcl
 opened on:  17 Feb 2026, 17:24:33

.
. capture drop Perc - CI_high_perc

. generate double Perc = .    // percentage transfusion 2+ units
(27,114 missing values generated)

. generate double Logit = .
(27,114 missing values generated)

. generate double CI_low_perc  = .
(27,114 missing values generated)

. generate double CI_high_perc = .
(27,114 missing values generated)

. forvalues i = 1/10 {
  2.     quietly ci proportions RBC_GE2 if Hgb_Decile == `i', wilson level(99)
  3.     quietly replace Perc = 100*   r(proportion)  if Hgb_Decile == `i' & Hgb_Decile != Hgb_Decile[_n-1]
  4.     quietly replace Logit = logit(r(proportion)) if Hgb_Decile == `i' & Hgb_Decile != Hgb_Decile[_n-1]
  5.     quietly replace CI_low_perc  = 100*r(lb)     if Hgb_Decile == `i' & Hgb_Decile != Hgb_Decile[_n-1]
  6.     quietly replace CI_high_perc = 100*r(ub)     if Hgb_Decile == `i' & Hgb_Decile != Hgb_Decile[_n-1]
  7. }

.
. twoway (scatter Perc MedianHgb, msymbol(O) mcolor(blue) msize(large))                                ///
>        (rcap CI_low_perc CI_high_perc MedianHgb, lcolor(ltblue) lwidth(medthick))                  ///
>        (scatter Logit MedianHgb, msymbol(o) mcolor(red) yaxis(2) msize(large))                      ///
>        (lfit Logit MedianHgb, lcolor(red) lwidth(medium) yaxis(2)),                                 ///
>        ylabel(0(2)14) xlabel(9.5(0.5)14.5, format(%4.1f)) legend(off)                               ///
>        ytitle("Percentage (%) of patients with {&ge}2 RBC transfusion", color(blue))                ///
>        xtitle("Median hemoglobin (g/dL) at admission for delivery")                                 ///
>        ytitle("Logit of proportion of patients with {&ge}2 RBC transfusion", color(red) axis(2))  ///
>        ylabel(-5.5(0.5)-2.0, format(%4.1f) axis(2))

. graph export  "06 OB anemia clinic Transfusion Log.pdf", as(pdf) name("Graph") replace // currently not in paper
file 06 OB anemia clinic Transfusion Log.pdf saved as PDF format

.
. capture log close
```

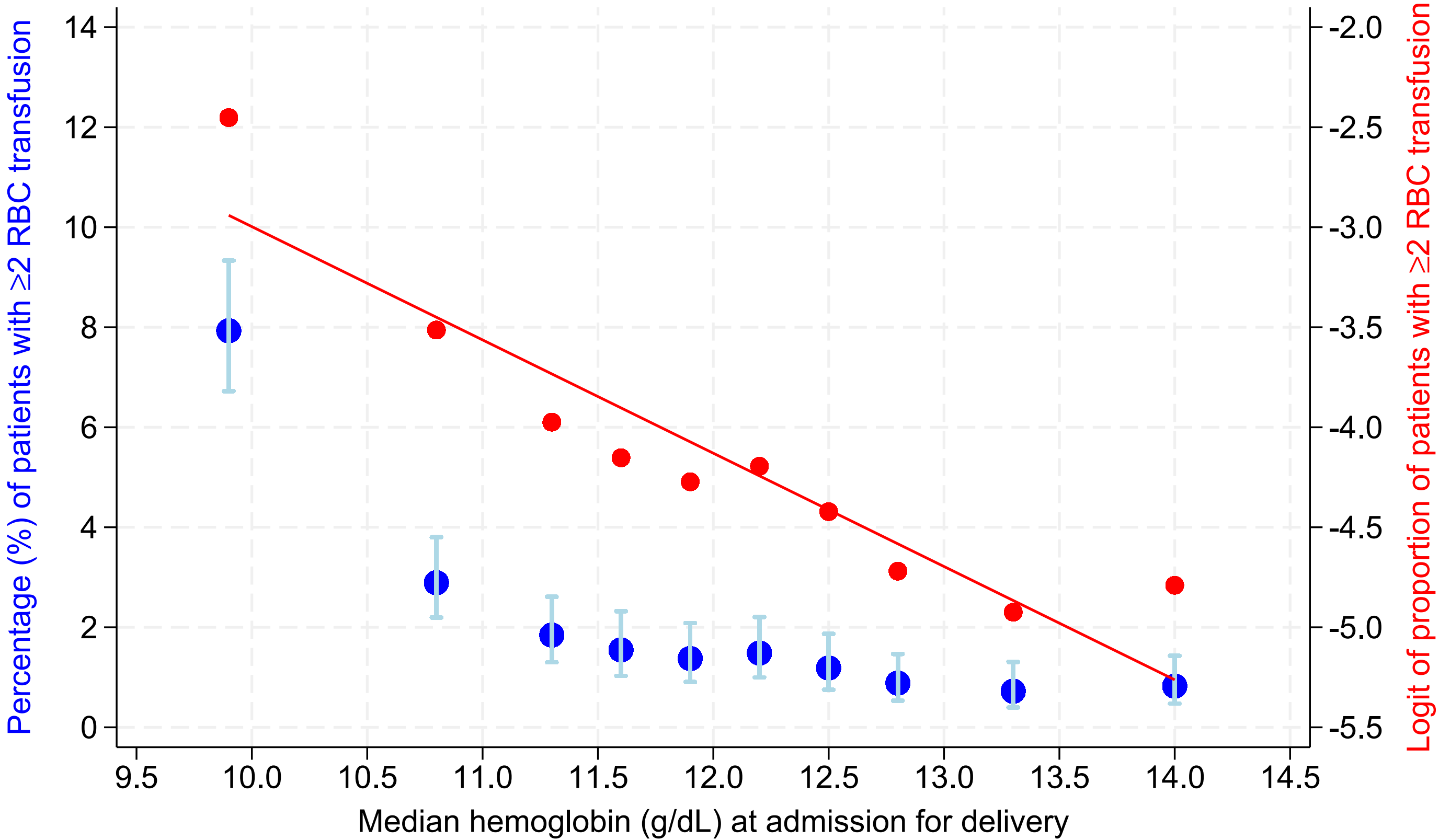

Percentage (%) of patients with ≥2 RBC transfusion
Logit of proportion of patients with ≥2 RBC transfusion
Median hemoglobin (g/dL) at admission for delivery
0
2
4
6
8
10
12
14
-2.0
-2.5
-3.0
-3.5
-4.0
-4.5
-5.0
-5.5
9.5
10.0
10.5
11.0
11.5
12.0
12.5
13.0
13.5
14.0
14.5

```
.
.
.
. *** Appendix S5 (Figure C), Initial antenatal Hgb associated with greater Prob(RBC transfusion during delivery) **
. use PAT_MRN_ID Hosp_Adm Hosp_DC Delivery using "Temp_Table 2.dta", clear

. display _N " records for later"
30591 records for later

.
. * merge with the Hgb file for every possible combination
. joinby PAT_MRN_ID using "Temp_Hemoglobins", unmatched(both) // using unmatched to get counts

.
. codebook _merge

-------------------------------------------------------------------------------
_merge                                                              (unlabeled)
-------------------------------------------------------------------------------

                  Type: Numeric (byte)
                 Label: __MERGE

                 Range: [1,3]                         Units: 1
         Unique values: 3                         Missing .: 0/185,062

            Tabulation: Freq.   Numeric  Label
                          625         1  only in master data
                            8         2  only in using data
                      184,429         3  both in master and using data

. drop if  _merge != 3
(633 observations deleted)

.
. generate double WkBeforeDeliv = (Delivery - HgbTime)/ (1000*60*60*24*7)

. label variable  WkBeforeDeliv "Weeks before delivery"

. drop if         WkBeforeDeliv < 0             // because prior delivery
(46,713 observations deleted)

. drop if         WkBeforeDeliv > 365*(9/12)/7 //  in data specification plan but was not applied
(32,014 observations deleted)

. gsort   PAT_MRN_ID                       Hosp_Adm -WkBeforeDeliv // saving earliest date (i.e., "Hgb" first antepartum)

. drop if PAT_MRN_ID == PAT_MRN_ID[_n-1] & Hosp_Adm == Hosp_Adm[_n-1]
(76,015 observations deleted)

.
. drop _merge Delivery HgbTime

.
. joinby PAT_MRN_ID using "Temp_Transfusions", unmatched(both)

. codebook _merge

-------------------------------------------------------------------------------
_merge                                                              (unlabeled)
-------------------------------------------------------------------------------

                  Type: Numeric (byte)
                 Label: __MERGE

                 Range: [1,3]                         Units: 1
         Unique values: 3                         Missing .: 0/31,417

            Tabulation: Freq.   Numeric  Label
                       27,957         1  only in master data
                           31         2  only in using data
                        3,429         3  both in master and using data

. * codebook       PAT_MRN_ID if _merge == 2                // RBC units transfused among patients withOUT Hgb measured
. quietly levelsof PAT_MRN_ID if _merge == 2, local(levels) // without displaying some

. local ndistinct : word count `levels'

. display "Distinct values in PAT_MRN_ID: `ndistinct'"
Distinct values in PAT_MRN_ID: 15

.
. drop if _merge == 2
(31 observations deleted)

.
. drop if (RBCtime < Hosp_Adm | RBCtime > Hosp_DC) & RBCtime != .
(878 observations deleted)

. sort            PAT_MRN_ID Hosp_Adm RBCtime
```

```
. by              PAT_MRN_ID Hosp_Adm, sort : egen int N_units = count(RBCtime)

. duplicates drop PAT_MRN_ID Hosp_Adm, force

Duplicates in terms of PAT_MRN_ID Hosp_Adm

(1,242 observations deleted)

.
. label variable N_units "RBC units transfused among patients with Hgb measured"

. assert         N_units != . & N_units >= 0

.
. generate byte Any_RBC_units = 0

. replace       Any_RBC_units = 1 if N_units > 0
(1,309 real changes made)

.
. generate byte RBC_GE2 = 0

. replace       RBC_GE2 = 1 if N_units > 1
(590 real changes made)

.
. ranksum Hgb, by(Any_RBC_units) porder // sixth paragraph Results first sentence

Two-sample Wilcoxon rank-sum (Mann-Whitney) test
```

Any_RBC_un~s	Obs	Rank sum	Expected
0	**27957**	**4.142e+08**	**4.091e+08**
1	**1309**	**14015998**	**19155252**
Combined	**29266**	**4.283e+08**	**4.283e+08**

```
Unadjusted variance   8.925e+10
Adjustment for ties   -66524664

Adjusted variance     8.919e+10

H0: Hgb(Any_RB~s==0) = Hgb(Any_RB~s==1)
         z = 17.209
Prob > |z| = 0.0000

P{Hgb(Any_RB~s==0) > Hgb(Any_RB~s==1)} = 0.640

.
. xtile Hgb_Decile = Hgb, nq(10)

. egen double MedianHgb = median(Hgb), by(Hgb_Decile)

. sort        MedianHgb N_units

.
. capture drop Perc - CI_high_perc

. generate double Perc = .
(29,266 missing values generated)

. generate double Logit = .
(29,266 missing values generated)

. generate double CI_low_perc  = .
(29,266 missing values generated)

. generate double CI_high_perc = .
(29,266 missing values generated)

. forvalues i = 1/10 {
  2.     quietly ci proportions Any_RBC_units if Hgb_Decile == `i', wilson level(99)
  3.     quietly replace Perc = 100*   r(proportion)  if Hgb_Decile == `i' & Hgb_Decile != Hgb_Decile[_n-1]
  4.     quietly replace Logit = logit(r(proportion)) if Hgb_Decile == `i' & Hgb_Decile != Hgb_Decile[_n-1]
  5.     quietly replace CI_low_perc  = 100*r(lb)     if Hgb_Decile == `i' & Hgb_Decile != Hgb_Decile[_n-1]
  6.     quietly replace CI_high_perc = 100*r(ub)     if Hgb_Decile == `i' & Hgb_Decile != Hgb_Decile[_n-1]
  7. }

.
. twoway (scatter Perc MedianHgb, msymbol(O) mcolor(blue) msize(large))                       ///
>        (rcap CI_low_perc CI_high_perc MedianHgb, lcolor(ltblue) lwidth(medthick))          ///
>        (scatter Logit MedianHgb, msymbol(o) mcolor(red) yaxis(2) msize(large))             ///
>        (lfit Logit MedianHgb, lcolor(red) lwidth(medium) yaxis(2)),                        ///
>        ylabel(0(2)14) xlabel(9.5(0.5)14.5, format(%4.1f)) legend(off)                      ///
>        ytitle("Percentage (%) of patients with RBC transfusion", color(blue))              ///
>        xtitle("Median hemoglobin (g/dL) at first antenatal visit")                         ///
>        ytitle("Logit of proportion of patients with RBC transfusion", color(red) axis(2)) ///
>        ylabel(-4.5(0.5)-1.0, format(%4.1f) axis(2))

. graph export "Out_Figure C v22.jpg"   , as(jpg) name("Graph") replace width(3600) height(2160) quality(100)
file Out_Figure C v22.jpg written in JPEG format

. graph export  "08 Figure C Export.pdf", as(pdf) name("Graph") replace
file 08 Figure C Export.pdf saved as PDF format

.
. capture log close
```

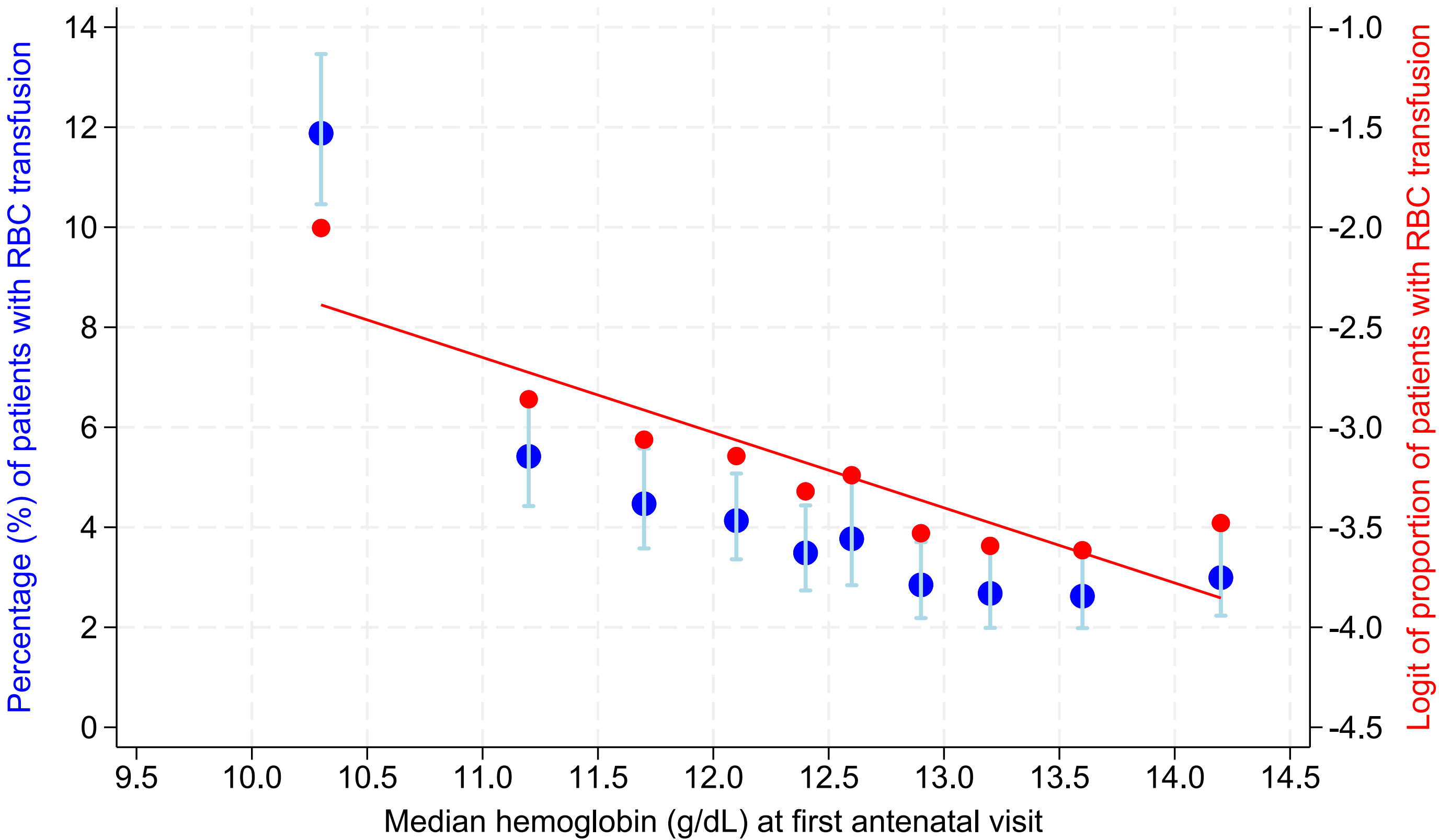

Percentage (%) of patients with RBC transfusion
Logit of proportion of patients with RBC transfusion
Median hemoglobin (g/dL) at first antenatal visit
0
2
4
6
8
10
12
14
-1.0
-1.5
-2.0
-2.5
-3.0
-3.5
-4.0
-4.5
9.5
10.0
10.5
11.0
11.5
12.0
12.5
13.0
13.5
14.0
14.5

```
.
. summarize WkBeforeDeliv, detail // Supplemental S5 legend, weeks before when first measure Hgb; not infuse, measure Hgb

                    Weeks before delivery
-------------------------------------------------------------
      Percentiles      Smallest
 1%     .0093254              0
 5%     .0580357       .0000992
10%     .2473677        .000172       Obs              29,266
25%     10.50581       .0001984       Sum of wgt.      29,266

50%     25.83993                      Mean           20.31446
                        Largest       Std. dev.      11.32218
75%     28.27133       39.06153
90%     31.31657       39.09286       Variance       128.1918
95%     33.00337       39.10278       Skewness       -.718479
99%     36.80968       39.10414       Kurtosis       2.045941

. spearman  WkBeforeDeliv Hgb     // Supplemental S5 legend comment that correlated

Number of observations = 29,266
        Spearman's rho = 0.3932

Test of H0: WkBeforeDeliv and Hgb are independent
                  Prob = 0.0000

.
. capture drop Perc - CI_high_perc // Additional figure not in the article

. generate double Perc = .
(29,266 missing values generated)

. generate double Logit = .
(29,266 missing values generated)

. generate double CI_low_perc  = .
(29,266 missing values generated)

. generate double CI_high_perc = .
(29,266 missing values generated)

. forvalues i = 1/10 {
  2.     quietly ci proportions RBC_GE2 if Hgb_Decile == `i', wilson level(99)
  3.     quietly replace Perc = 100*   r(proportion)  if Hgb_Decile == `i' & Hgb_Decile != Hgb_Decile[_n-1]
  4.     quietly replace Logit = logit(r(proportion)) if Hgb_Decile == `i' & Hgb_Decile != Hgb_Decile[_n-1]
  5.     quietly replace CI_low_perc  = 100*r(lb)     if Hgb_Decile == `i' & Hgb_Decile != Hgb_Decile[_n-1]
  6.     quietly replace CI_high_perc = 100*r(ub)     if Hgb_Decile == `i' & Hgb_Decile != Hgb_Decile[_n-1]
  7. }

.
. twoway (scatter Perc MedianHgb, msymbol(O) mcolor(blue) msize(large))                               ///
>        (rcap CI_low_perc CI_high_perc MedianHgb, lcolor(ltblue) lwidth(medthick))                   ///
>        (scatter Logit MedianHgb, msymbol(o) mcolor(red) yaxis(2) msize(large))                      ///
>        (lfit Logit MedianHgb, lcolor(red) lwidth(medium) yaxis(2)),                                 ///
>        ylabel(0(2)18) xlabel(9.5(0.5)14.5, format(%4.1f)) legend(off)                               ///
>        ytitle("Percentage (%) of patients with {&ge}2 RBC transfusion", color(blue))                ///
>        xtitle("Median hemoglobin (g/dL) at first antenatal visit")                                  ///
>        ytitle("Logit of proportion of patients with {&ge}2 RBC transfusion", color(red) axis(2)) ///
>        ylabel(-5.0(0.5)-1.5, format(%4.1f) axis(2))

. graph export  "10 OB anemia clinic Transfusion Log.pdf", as(pdf) name("Graph") replace // currently not in paper
file 10 OB anemia clinic Transfusion Log.pdf saved as PDF format

.
. capture log close
```

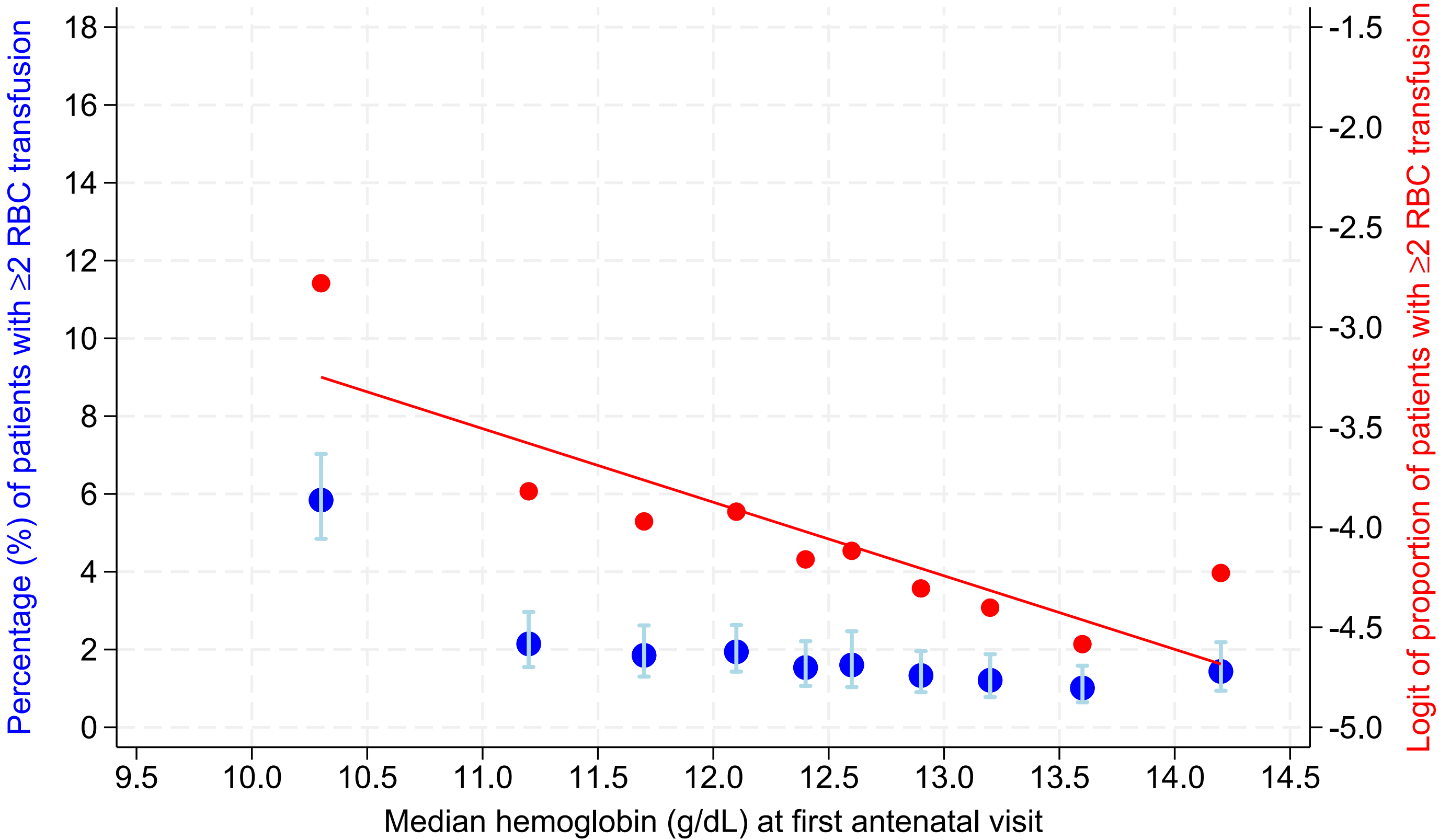

Percentage (%) of patients with ≥2 RBC transfusion
Logit of proportion of patients with ≥2 RBC transfusion
Median hemoglobin (g/dL) at first antenatal visit
0
2
4
6
8
10
12
14
16
18
-5.0
-4.5
-4.0
-3.5
-3.0
-2.5
-2.0
-1.5
9.5
10.0
10.5
11.0
11.5
12.0
12.5
13.0
13.5
14.0
14.5

```
.
.
.
. * Supplemental S6 (Figure D), Sufficient time for Rx at anemia clinic, since only need a few weeks ***************
. summarize WkBeforeDeliv, detail

                    Weeks before delivery
-------------------------------------------------------------
      Percentiles      Smallest
 1%     .0093254              0
 5%     .0580357       .0000992
10%     .2473677        .000172       Obs              29,266
25%     10.50581       .0001984       Sum of wgt.      29,266

50%     25.83993                      Mean           20.31446
                        Largest       Std. dev.      11.32218
75%     28.27133       39.06153
90%     31.31657       39.09286       Variance       128.1918
95%     33.00337       39.10278       Skewness       -.718479
99%     36.80968       39.10414       Kurtosis       2.045941

.
. use "Temp_Table S3.dta", clear

. joinby PAT_MRN_ID using "Temp_Anemia clinic", unmatched(both)

. codebook _merge

-------------------------------------------------------------------------------------------------------------------------------------
_merge                                                                                                                    (unlabeled)
-------------------------------------------------------------------------------------------------------------------------------------

                  Type: Numeric (byte)
                 Label: __MERGE

                 Range: [1,3]                         Units: 1
         Unique values: 3                         Missing .: 0/37,342

            Tabulation: Freq.   Numeric  Label
                       24,543         1  only in master data
                           96         2  only in using data
                       12,703         3  both in master and using data

. drop if  _merge != 3 // data in master and from anemia clinic
(24,639 observations deleted)

.
. generate double FeWkBeforeDeliv = (Delivery - FeTime)/(1000*60*60*24*7)

. label variable  FeWkBeforeDeliv "Weeks (double) from Fe infusion to delivery"

. drop if         FeWkBeforeDeliv > DeliveryGestAge/7 //  in data specification plan but was not applied
(1,712 observations deleted)

. drop if         FeWkBeforeDeliv < 0                 //  another pregnancy
(3,073 observations deleted)

. assert          FeWkBeforeDeliv != 0

.
. generate double FeDay = dofc(FeTime)

. format          FeDay %td

. sort PAT_MRN_ID Hosp_Adm FeDay // required sequence for next command

. by   PAT_MRN_ID Hosp_Adm FeDay: generate byte is_unique_Fe_day = (_n == 1)

. by PAT_MRN_ID Hosp_Adm, sort: egen n_visits = total(is_unique_Fe_day)

. label variable                     n_visits "Count of anemia clinic visits for IV Fe infusion"

.
. by PAT_MRN_ID Hosp_Adm, sort : egen int Total_ml_IV_Fe = total(IV_Fe_mL)

. label variable                          Total_ml_IV_Fe "Total ml IV Fe at anemia clinic"

.
. gsort PAT_MRN_ID Hosp_Adm -FeWkBeforeDeliv

. drop if PAT_MRN_ID == PAT_MRN_ID[_n-1] & Hosp_Adm == Hosp_Adm[_n-1]
(6,107 observations deleted)

.
. generate double FeStartWkPostConcep = DeliveryGestAge/7 - FeWkBeforeDeliv

. format %17.2f   FeStartWkPostConcep

. label variable  FeStartWkPostConcep "Fe infusion started these weeks post conception"
```

```
.
. drop _merge FeTime IV_Fe_mL DeliveryGestAge FeDay is_unique_Fe_day

.
. summarize n_visits FeWkBeforeDeliv FeStartWkPostConcep, detail // Legend S6 second sentence

      Count of anemia clinic visits for IV Fe infusion
-------------------------------------------------------------
      Percentiles      Smallest
 1%            1              1
 5%            1              1
10%            1              1       Obs               1,811
25%            1              1       Sum of wgt.       1,811

50%            1                      Mean           1.917725
                        Largest       Std. dev.      1.311391
75%            3              8
90%            4              8       Variance       1.719746
95%            4              8       Skewness       1.770767
99%            6             14       Kurtosis       8.426775

         Weeks (double) from Fe infusion to delivery
-------------------------------------------------------------
      Percentiles      Smallest
 1%     .1785615       .0598793
 5%     .6661706       .0804448
10%     1.217627       .1028191       Obs               1,811
25%     2.736485       .1040493       Sum of wgt.       1,811

50%     5.054762                      Mean            6.46793
                        Largest       Std. dev.      5.613586
75%     8.556824       37.05358
90%     12.76627         37.452       Variance       31.51234
95%     17.87143       37.50448       Skewness       2.082222
99%     27.81369       38.72548       Kurtosis       9.106542

       Fe infusion started these weeks post conception
-------------------------------------------------------------
      Percentiles      Smallest
 1%     8.748163       .2098082
 5%     21.01703       .6908565
10%     25.00197        .703087       Obs               1,811
25%     29.86278       .8526389       Sum of wgt.       1,811

50%     33.19198                      Mean           31.90826
                        Largest       Std. dev.      5.698544
75%     35.61644       39.72225
90%     37.38437        39.8753       Variance        32.4734
95%     38.09506       40.26944       Skewness      -1.991343
99%     39.08664       40.34846       Kurtosis       8.911997

. * Manually checked the case with smallest week post conception and confirmed (FD 07/19/25)
.
. generate byte  HgbGE11 = 0

. replace        HgbGE11 = 1 if Hgb_Adm >= 11
(1,221 real changes made)

. label variable HgbGE11 "Hgb_Adm >=11 g/DL upon admission for delivery & patient Rx IV Fe infusion"

.
. xtile Wk_Quin = FeStartWkPostConcep, nq(5) // Quintile of week post-conception

. sort  Wk_Quin   FeStartWkPostConcep

. egen double MedianWkPost    = median(FeStartWkPostConcep), by(Wk_Quin)

. egen double MedianWkBeforeDeliv = median(FeWkBeforeDeliv), by(Wk_Quin)

. replace     MedianWkPost        = . if Wk_Quin == Wk_Quin[_n-1]
(1,806 real changes made, 1,806 to missing)

. replace     MedianWkPost        = round(MedianWkPost, 0.001) // for use in figure upper label below
(5 real changes made)

. replace     MedianWkBeforeDeliv = . if Wk_Quin == Wk_Quin[_n-1]
(1,806 real changes made, 1,806 to missing)

.
. capture drop Perc - CI_high_erc

. generate double Perc = .
(1,811 missing values generated)

. generate double Logit = .
(1,811 missing values generated)

. generate double CI_low_perc  = .
(1,811 missing values generated)

. generate double CI_high_perc = .
(1,811 missing values generated)

. forvalues i = 1/5 {
  2.     quietly ci proportions HgbGE11 if Wk_Quin == `i', wilson level(99)
  3.     quietly replace Perc = 100*   r(proportion)  if Wk_Quin == `i' & Wk_Quin != Wk_Quin[_n-1]
  4.     quietly replace Logit = logit(r(proportion)) if Wk_Quin == `i' & Wk_Quin != Wk_Quin[_n-1]
  5.     quietly replace CI_low_perc  = 100*r(lb)     if Wk_Quin == `i' & Wk_Quin != Wk_Quin[_n-1]
  6.     quietly replace CI_high_perc = 100*r(ub)     if Wk_Quin == `i' & Wk_Quin != Wk_Quin[_n-1]
  7. }
```

```
.
. preserve // to create temporary data file for the Supplemental S6, Figure D

. keep MedianWkPost-CI_high_perc

. drop if MedianWkPost == .
(1,806 observations deleted)

. save "Figure D.dta", replace
(file Figure D.dta not found)
file Figure D.dta saved

. restore

.
. * Figure version currently not used
. twoway (scatter Perc MedianWkPost, msymbol(O) mcolor(blue) msize(large))                          ///
>        (rcap CI_low_perc CI_high_perc MedianWkPost, lcolor(ltblue) lwidth(medthick))              ///
>        (scatter Logit MedianWkPost, msymbol(o) mcolor(red) yaxis(2) msize(large))                 ///
>        (lfit Logit MedianWkPost, lcolor(red) lwidth(medium) yaxis(2)),                            ///
>        ylabel(45(5)85) xscale(reverse) legend(off) ylabel(0(0.2)1.6, format(%4.1f) axis(2))       ///
>        ytitle("% patients Hgb {&ge}11 g/dL, upon admission", color(blue))                         ///
>        ytitle("Logit of proportion of patients Hgb {&ge}11 g/dL", color(red) axis(2))             ///
>        xtitle("First IV Iron infusion date as weeks post-conception" "(as weeks before delivery)") ///
>        xlabel(24 "24 (13.7)" 26 "26 (12.0)" 28 "28 (10.2)" 30 "30 (8.4)" 32 "32 (6.7)"            ///
>               34 "34 (4.9)" 36 "36 (3.1)" 38 "38 (1.3)") plotregion(margin(r+5))

.
. * Revised S6 Figure from RHE v20250824-1306, primary and secondary horizontal axes' different reverse sequence order
. twoway (scatter Perc MedianWkPost, msymbol(O) mcolor(blue) msize(large))              ///
>        (rcap CI_low_perc CI_high_perc MedianWkPost, lcolor(blue) lwidth(medium))      ///
>        (scatter Logit MedianWkPost, msymbol(o) mcolor(red) yaxis(2) msize(large))     ///
>        (lfit Logit MedianWkPost, lcolor(red) lwidth(medium) yaxis(2))                 ///
>        (pci 85 38 86 38, lcolor(black) lwidth(thin) lpattern(solid))                  ///
>        (pci 85 36 86 36, lcolor(black) lwidth(thin) lpattern(solid))                  ///
>        (pci 85 34 86 34, lcolor(black) lwidth(thin) lpattern(solid))                  ///
>        (pci 85 32 86 32, lcolor(black) lwidth(thin) lpattern(solid))                  ///
>        (pci 85 30 86 30, lcolor(black) lwidth(thin) lpattern(solid))                  ///
>        (pci 85 28 86 28, lcolor(black) lwidth(thin) lpattern(solid))                  ///
>        (pci 85 26 86 26, lcolor(black) lwidth(thin) lpattern(solid))                  ///
>        (pci 85 24 86 24, lcolor(black) lwidth(thin) lpattern(solid)),                 ///
>        ylabel(45"45" 50"50" 55"55" 60"60" 65"65" 70"70" 75"75" 80"80" 85"85" 90" ",   ///
>               labsize(small)) xscale(reverse) legend(off)                             ///
>        ylabel(0.0"0.0" 0.2"0.2" 0.4"0.4" 0.6"0.6" 0.8"0.8" 1.0"1.0" 1.2"1.2" 1.4"1.4" 1.6"1.6" 1.8" ", ///
>               labsize(small) format(%4.1f) axis(2))                                   ///
>        ytitle("% patients Hgb {&ge}11 g/dL, upon admission", color(blue))             ///
>        ytitle("Logit of proportion of patients Hgb {&ge}11 g/dL", color(red) axis(2))  ///
>        yline(85, lcolor(black) lwidth(thin) lpattern(solid) )                         ///
>        xlabel(38(-2)24, labcolor(black) labsize(small))                               ///
>        xtitle("First IV Iron infusion date as weeks post-conception", color(black) size(medium)) ///
>        yscale(range(45(5)85) noextend)                  ///
>        yscale(range(0(0.2)1.6) axis(2) noextend)        ///
>        text(88 37.95 "1.3" , color(black) size(small) ) ///
>        text(88 35.95 "3.1" , color(black) size(small) ) ///
>        text(88 33.95 "4.9" , color(black) size(small) ) ///
>        text(88 31.95 "6.7" , color(black) size(small) ) ///
>        text(88 29.95 "8.4" , color(black) size(small) ) ///
>        text(88 27.95 "10.2", color(black) size(small) ) ///
>        text(88 25.95 "12.0", color(black) size(small) ) ///
>        text(88 23.95 "13.7", color(black) size(small) ) ///
>        title("First IV Iron infusion date as weeks before delivery", color(black) size(medium)) ///
>        plotregion(margin(r+5))

. graph export "Out_Figure D v28.jpg"   , as(jpg) name("Graph") replace width(3600) height(2160) quality(100)
file Out_Figure D v28.jpg written in JPEG format

. graph export  "12 Figure D Export.pdf", as(pdf) name("Graph") replace
file 12 Figure D Export.pdf saved as PDF format

.
. capture log close
```

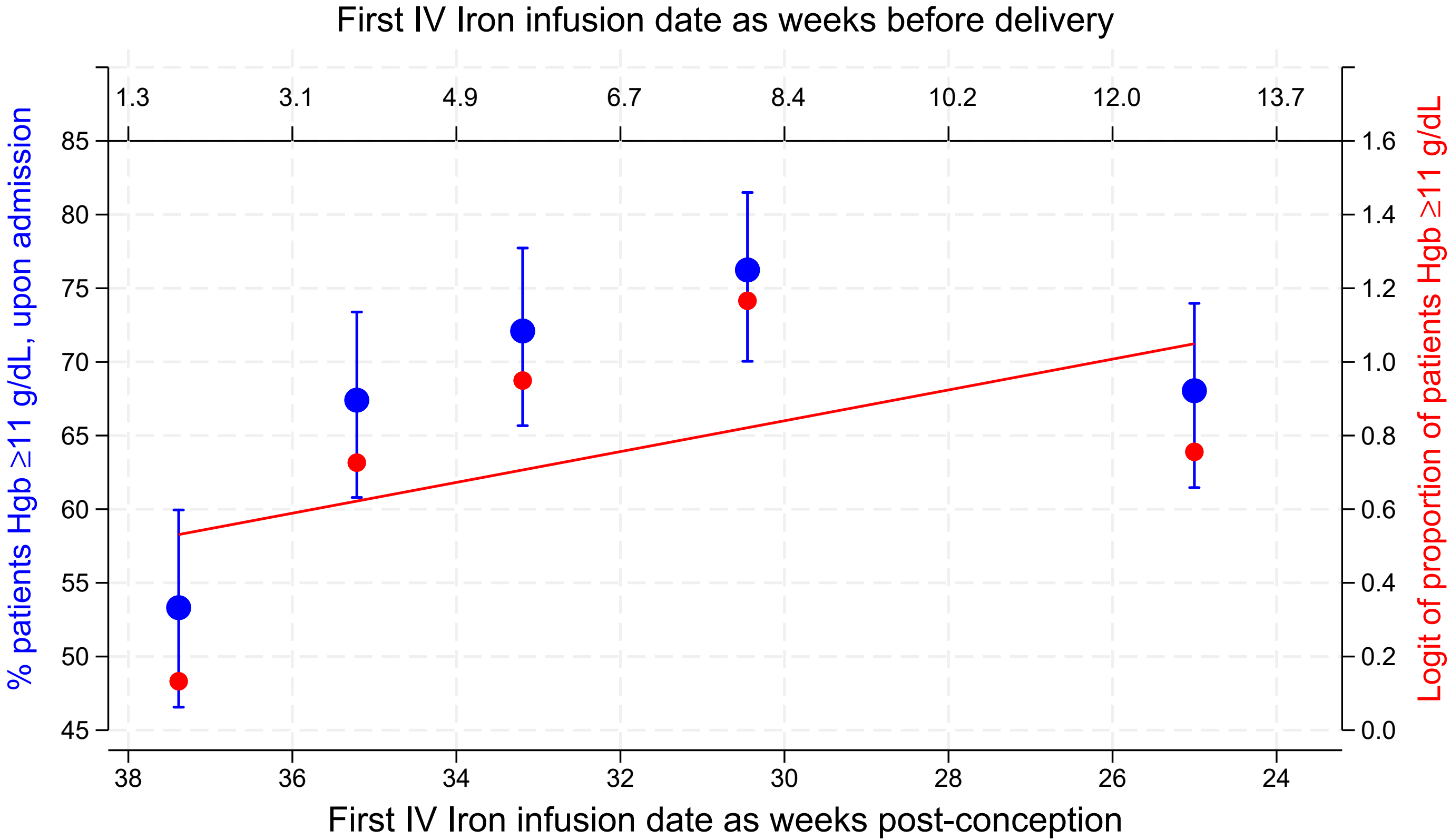

First IV Iron infusion date as weeks before delivery
1.3
3.1
4.9
6.7
8.4
10.2
12.0
13.7
% patients Hgb ≥11 g/dL, upon admission
85
80
75
70
65
60
55
50
45
Logit of proportion of patients Hgb ≥11 g/dL
1.6
1.4
1.2
1.0
0.8
0.6
0.4
0.2
0.0
38
36
34
32
30
28
26
24
First IV Iron infusion date as weeks post-conception

```
.
. spearman FeWkBeforeDeliv     Hgb_Adm // Supplemental S6 legend and sixth paragraph results second sentence

Number of observations =  1,811
        Spearman's rho = 0.4027

Test of H0: FeWkBeforeDeliv and Hgb_Adm are independent
                  Prob = 0.0000

. display "P = " %6.5f r(p)
P = 0.00000

. spearman FeStartWkPostConcep Hgb_Adm

Number of observations =   1,811
        Spearman's rho = -0.1799

Test of H0: FeStartWkPostConcep and Hgb_Adm are independent
                  Prob =  0.0000

. display "P = " %6.5f 4*r(p)
P = 0.00000

.
. ranksum FeWkBeforeDeliv,     by(HgbGE11) porder // Supplemental S6 legend

Two-sample Wilcoxon rank-sum (Mann-Whitney) test

     HgbGE11 |       Obs    Rank sum    Expected
-------------+---------------------------------
           0 |       590      383236      534540
           1 |      1221     1257530     1106226
-------------+---------------------------------
    Combined |      1811     1640766     1640766

Unadjusted variance   1.088e+08
Adjustment for ties  -.10988556
                     ----------
Adjusted variance     1.088e+08

H0: FeWkBe~v(HgbGE11==0) = FeWkBe~v(HgbGE11==1)
         z = -14.507
Prob > |z| =  0.0000

P{FeWkBe~v(HgbGE11==0) > FeWkBe~v(HgbGE11==1)} = 0.290

. display "P = " %6.5f 4*r(p)
P = 0.00000

. ranksum FeStartWkPostConcep, by(HgbGE11) porder

Two-sample Wilcoxon rank-sum (Mann-Whitney) test

     HgbGE11 |       Obs    Rank sum    Expected
-------------+---------------------------------
           0 |       590      589491      534540
           1 |      1221     1051275     1106226
-------------+---------------------------------
    Combined |      1811     1640766     1640766

Unadjusted variance   1.088e+08
Adjustment for ties           0
                     ----------
Adjusted variance     1.088e+08

H0: FeStar~p(HgbGE11==0) = FeStar~p(HgbGE11==1)
         z =   5.269
Prob > |z| = 0.0000

P{FeStar~p(HgbGE11==0) > FeStar~p(HgbGE11==1)} = 0.576

. display "P = " %6.5f 4*r(p)
P = 0.00000

.
. capture log close
```
