# Supplementary material for: Timing of intrapartum transfusion among obstetric patients, stratified by admission and discharge haemoglobin, relative to anaesthesia care: A retrospective cohort study
Source: Transfus Med. 2026 Apr 26;36(5):475–83. doi: 10.1111/tme.70082 (PMC13670405; doi:10.1111/tme.70082)
Supplement: Supplementary file 2 [file TME-36-475-s002.docx]

**S1. University of Iowa Obstetric hemorrhage management guide, version 2.0 updated October 2022**

| **Stage 2**  **1500-2499 mL** | **OBSTETRICAL PROVIDER:**   - Promptly address bleeding source - Utilize a device (Jada or Bakri) for uterine atony, if not already placed - Determine need for help from Gyn Oncology or other surgical teams - If DIC suspected (abruption, AFE), consider RiaSTAP | **NURSE to activate Stage II Hemorrhage Orders:**   - Announce **Bleed Time Out** at 1500 mL QBL - Tranexamic Acid (TXA) 1 g - Re-dose Cefazolin (2 or 3g) - Obtain 2u RBCs from Kiosk - Ask OB and Anesthesia if additional blood needed | **ANESTHESIA PROVIDER:**   - Administer Tranexamic Acid - Re-dose Cefazolin - Provide volume support   to maintain hemodynamic stability   - Transfuse 1-2u RBCs if hemodynamic instability and ongoing bleeding - Repeat PPH labs every 1L blood loss | |
| --- | --- | --- | --- | --- |
| *Blood loss above 2500mL is a* ***Massive Maternal Hemorrhage*** *– Activate* ***CODE HEMORRHAGE*** *to obtain additional help* | | | |  |
| **Stage 3**  **2500 mL and above** | **OBSTETRICAL PROVIDER:**   - Compress bleeding source and HOLD PRESSURE   - Bimanual uterine compression if atony   - Apply constant pressure to other bleeding sources with laparotomy sponges - Request Gyn Oncology assistance - Give SBAR to Code Hemorrhage Team - Resume efforts at bleeding control once patient is hemodynamically stable - Consider RiaSTAP if DIC suspected | **NURSE to activate Stage III Hemorrhage Orders:**   - Announce **Bleed Time Out** at 2500mL QBL and with new lab results - Activate CODE HEMORRHAGE - Order MTP - Obtain hysterectomy and vascular instrument trays - Refer to CODE HEMORRHAGE Guideline for additional tasks - Ask about RiaSTAP - Ask about ICU bed | **ANESTHESIA PROVIDER:**   - Transfuse 1u RBC empirically if ongoing bleeding - Don “Red Cap” and delegate tasks to Code Hemorrhage providers - Consider RiaSTAP if DIC suspected - Repeat PPH Labs every 1L blood loss |  |

**S2. PubMed search evaluating earlier studies on the timing of intrapartum RBC transfusion**

Search protocol

( "Erythrocyte Transfusion"[Mesh] OR "red blood cell transfusion"[TIAB] OR "erythrocyte transfusion"[TIAB] OR "RBC transfusion"[TIAB] OR "PRBC transfusion"[TIAB] OR "packed red blood cell*"[TIAB] OR "allogeneic blood"[TIAB] )

AND

( intrapartum[TIAB] OR "Peripartum Period"[Mesh] OR "Postpartum Period"[Mesh] OR "Delivery, Obstetric"[Mesh] OR "Postpartum Hemorrhage"[Mesh] OR "Labor, Obstetric"[Mesh] OR peripartum[TIAB] OR postpartum[TIAB] OR delivery[TIAB] OR obstetric*[TIAB] )

AND

( "timing transfusion"[TI:~2] OR "timing transfusions"[TI:~2] OR "timing RBC"[TI:~2] OR "timing PRBC"[TI:~2] OR "time transfusion"[TI:~2] OR "time transfusions"[TI:~2] OR "time RBC"[TI:~2] OR "time PRBC"[TI:~2] OR "when transfusion"[TI:~2] OR "when transfusions"[TI:~2] OR "when RBC"[TI:~2] OR "when PRBC"[TI:~2] OR "interval transfusion"[TI:~2] OR "interval transfusions"[TI:~2] OR "interval RBC"[TI:~2] OR "interval PRBC"[TI:~2] )

Among the seven results (on 23 December 2025), the one related study had older transfusion practices, including more RBC units:^10^ The following are quotations from the abstract of the cohort study of 127 patients with hemorrhage during cesarean delivery:

Of these subjects, 103/127 received blood because of intraoperative hemorrhage, a reduction in the hematocrit of more than 10 points, or because the postpartum hematocrit was < 24%. These subjects were compared with the remaining women (n = 24) who met the same criteria for hemorrhage but did not receive transfusion … The estimated blood loss (873 ± 484 ml) and the antepartum hematocrits (33.4% ± 6.4%) in the women who did not receive transfusion were not significantly different from those of patients who received packed red blood cells (854 ± 576 ml and 30.0% ± 5.4%, respectively) … Patients in the transfused group received a mean of 3.8 ± 4.9 RBC units … The mean equilibrated (stable) hematocrit after transfusion was 28.4% ± 5.4%.

**S3. Characteristics of studied deliveries, vaginal or cesarean delivery, with or without an anesthesiologist, among patients with *hemoglobin determinations on admission or within one week before admission***

| **Variable** | **% (n/N) or N** | **Median [25^th^, 75^th^ percentiles]** | **Mean (standard deviation)** |
| --- | --- | --- | --- |
| Cesarean birth | 37% (10,155/27,114) |  |  |
| Anesthesiologist (billed) care during hospitalization | 91% (24,794/27,114) |  |  |
| Anesthesiologist care at time of delivery | 73% (19,844/27,114) |  |  |
|  |  |  |  |
| Maternal age | 27,114 | 30 [26,33] | 29.7 (5.5) |
| Parity | 27.,048 | 2 [1,3] | 2.1 (1.3) |
| Admitted hours before delivery^a^ | 27,114 | 11.7 [5.3, 21.9] | 30.4 (98.2) |
| Discharge hours after delivery^a^ | 27,114 | 50.5 [40.6, 66.3] | 54.2 (26.7) |
| Any RBC transfused | 4.7% (1,279/27,114) |  |  |
| Any RBC transfused  during anesthesia care | 1.0% (279/27,114) |  |  |
| ≥2 units RBC transfused | 2.1% (576/27,114) |  |  |
| ≥2 units RBC transfused during anesthesia care | 0.4% (107/27,114) |  |  |
| RBC units transfused if any | 1279 | 1 [1,2] | 1.95 (1.93) |
| RBC units transfused, if any, during anesthesia care | 279 | 1 [1,2] | 1.62 (1.28) |
| Quantitative blood loss in mL | 18,167 | 449 [244, 794] | 604 (540) |
| Blood loss, quantitative and otherwise estimate^b^ | 25,890 | 427 [250, 800] | 589 (533) |
| Allowable blood loss (ml), from Russo et al.^12,c^ | 26,588 | 2,546 [2291, 2769] | 2,489 (396) |
| Hgb at admission (g/dL)^d^ | 27,114 | 12.0 [11.2, 12.8] | 12.0 (1.2) |
| Anemic at admission^b^ | 19% (5,203/27,114) |  |  |

RBC represents packed red blood cells; Hgb represents hemoglobin

^a^	If >1 births during a delivery (e.g., twins), the time used was the birth of the first baby.

^b^	The anemic patients had significantly greater blood loss than the non-anemic patients, 638 (579) versus 577 (546), P <0.0001 by Wilcoxon-Mann-Whitney test. This result demonstrates predictive validity, as it aligns with findings from an earlier meta-analysis using different populations.^21^

^c^	For the 9/26,588 observations with the last hemoglobin before delivery ≤7 g/dL, the negatively valued allowable blood loss^12^ was set equal to zero.

^d^	The hemoglobin measurement used was the latest before delivery, but no earlier than one week before. The median was 1.4 [1.0, 2.0] hours after admission, and the mean was 2.1 (14.5) hours. For 98.7% (26,753/27,114), this admission hemoglobin ≥ 9 g/dL, the previously validated departmental anesthesiologist audit criteria for RBC transfusion.^1^

**S4. Characteristics of studied deliveries, vaginal or cesarean delivery, with or without an anesthesiologist, among patients with *hemoglobin determinations both on admission and before discharge***

| **Variable** | **% (n/N) or N** | **Median [25^th^, 75^th^ percentiles]** | **Mean (standard deviation)** |
| --- | --- | --- | --- |
| Cesarean birth | 61% (2,934/4,817) |  |  |
| Anesthesiologist (billed) care during hospitalization | 92% (4,450/4,817) |  |  |
| Anesthesiologist care at time of delivery | 81% (3,910/4,817) |  |  |
|  |  |  |  |
| Maternal age | 4,817 | 30 [26, 34] | 30.3 (5.7) |
| Parity | 4,810 | 2 [1,3] | 2.1 (1.4) |
| Admitted hours before delivery^a^ | 4,817 | 16.3 [5.5, 33.8] | 47.6 (132.3) |
| Discharge hours after delivery^a^ | 4,817 | 67.0 [51.4, 78.0] | 69.1 (36.6) |
| Any RBC transfused | 20.9% (1,008/4,817) |  |  |
| Any RBC transfused during anesthesia care | 5.3% (257/4,817) |  |  |
| ≥2 units RBC transfused | 10.2% (490/4,817) |  |  |
| ≥2 units RBC transfused during anesthesia care | 2.1% (101/4,817) |  |  |
| RBC units transfused if any | 988 | 1 [1,2] | 2.05 (1.95) |
| RBC units transfused, if any, during anesthesia care | 257 | 1 [1,2] | 1.65 (1.32) |
| Quantitative blood loss in mL | 3,857 | 1077 [630, 1534] | 1167 (769) |
| Blood loss, quantitative and otherwise estimated | 4,647 | 1024 [600, 1500] | 1142 (924) |
| Allowable blood loss (ml), from Russo et al.^12,b^ | 4,680 | 2,485 [2186, 2716] | 2,409 (460) |
| Hgb at admission (g/dL)^c^ | 4,817 | 11.8 [10.9, 12.6] | 11.7 (1.3) |
| Anemic at admission, Hgb < 11 g/dL^d^ | 25% (1,225/4,817) |  |  |
| Hgb before discharge (g/dL) | 4,817 | 10.3 [9.3, 11.3] | 10.3 (1.5) |
| Hgb < 9 g/dL on discharge (i.e., moderate or severe anemia)^13,d^ | 18% (862/4,817) |  |  |
| Hgb < 8 g/dL on discharge (i.e., severe anemia)^14^ | 6% (286/4,817) |  |  |

RBC represents packed red blood cells; Hgb represents hemoglobin

^a^	If >1 births during a delivery (e.g., twins), the time used was the birth of the first baby.

^b^	For the 5/4,680 observations with the last hemoglobin before delivery ≤7 g/dL, the negatively valued allowable blood loss^12^ was set equal to zero.

^c^	The hemoglobin measurement used was the earliest after admission but before discharge. If not done, then the last hemoglobin before admission was used, but no more than one week before. The median was 1.4 [1.0, 2.0] hours after admission, and the mean was 2.1 (14.5) hours.

^d^	The patients who were anemic at admission had significantly greater odds of moderate or severe anemia upon discharge (estimated two group odds ratio 6.48, 99% confidence interval 5.27 to 7.97, Fisher’s exact two-sided P <0.0001). This result demonstrates predictive validity, as it aligns with findings from an earlier machine learning study of a different cohort of patients who did not receive RBC transfusions.^22^ Variable importance study with elastic net regression showed that pre-labor hemoglobin was by far the most important predictor of post-delivery hemoglobin.^22^ In addition, adjusting for 13 patient factors, prepartum hemoglobin 10 to 10.9 g/dL and <10 g/dL had odds ratios of 5.38 (95% lower confidence limit 4.89) and 30.6 (27.1) for postpartum hemoglobin <8 g/dL before discharge.^14^

**S5. First antenatal hemoglobin <11 g/dL was associated with greater probability of RBC transfusion during the hospitalization that included the delivery**

RBC stands for red blood cells. The estimated percentages are shown along with Wilson-method 99% two-sided confidence intervals, one per decile of hemoglobin. The median hemoglobin is plotted. The graphical relationships are the same when plotted for two or more RBC units transfused. The logit is plotted on the right to differentiate it from Figure 1 and to show that, generally, they were close to linear with all graphed proportions. The N=29,266 patients had first hemoglobin measured median 25.8 [10.5, 28.3], mean 20.3 (11.3) weeks before delivery. However, the relationship between weeks and transfusion is complicated because patients presenting earlier had significantly greater first antenatal hemoglobin, Spearman ρ = 0.39, P <0.0001.

**S6. Intravenous iron infusions starting even 4-6 weeks before delivery were associated with significantly reduced incidences of anemia at admission for delivery**

As explained in the S5 legend, the N=29,266 patients had their first antenatal hemoglobin measured at a median of 25.8 [10.5, 28.3] weeks before delivery. The N=1,811 who also had intravenous iron infusion before delivery, had median 1 [1,3] mean 1.9 (SD 1.3) visits to the anemia management clinic, the first a median of 5.1 [2.7, 8.6] mean 6.5 (5.6) weeks before delivery, at median 33.2 [29.9, 35.6] mean 31.9 (SD 5.7) weeks post-conception. The anemia management clinic had a nurse practitioner and a medical assistant (doi). Intravenous iron infusion(s) starting more weeks before delivery or earlier in weeks post-conception were associated with greater hemoglobin measured at admission for delivery (Spearman ρ = 0.40 and ‑0.18, respectively), both P <0.0001 after Bonferroni-adjustment for four comparisons. By Wilcoxon-Mann-Whitney tests, intravenous iron infusion(s) starting more weeks before delivery or earlier in weeks post-conception were associated with a lower probability of the patient being anemic (hemoglobin < 11 g/dL) at admission for delivery, both P < 0.0001 after Bonferroni adjustment for four comparisons.
